# Respiratory CO_2_ Combined With a Blend of Volatiles Emitted by Endophytic *Serendipita* Strains Strongly Stimulate Growth of *Arabidopsis* Implicating Auxin and Cytokinin Signaling

**DOI:** 10.3389/fpls.2020.544435

**Published:** 2020-09-02

**Authors:** Jolien Venneman, Lore Vandermeersch, Christophe Walgraeve, Kris Audenaert, Maarten Ameye, Jan Verwaeren, Kathy Steppe, Herman Van Langenhove, Geert Haesaert, Danny Vereecke

**Affiliations:** ^1^Laboratory of Applied Mycology and Phenomics, Department of Plants and Crops, Faculty of Bioscience Engineering, Ghent University, Ghent, Belgium; ^2^Research Group EnVOC, Department of Green Chemistry and Technology, Faculty of Bioscience Engineering, Ghent University, Ghent, Belgium; ^3^Department of Data Analysis and Mathematical Modelling, Faculty of Bioscience Engineering, Ghent University, Ghent, Belgium; ^4^Laboratory of Plant Ecology, Department of Plants and Crops, Faculty of Bioscience Engineering, Ghent University, Ghent, Belgium

**Keywords:** endophytic Sebacinales, fungal volatiles, phytohormone signaling, *Piriformospora*, plant growth and development, plant-microbe interactions

## Abstract

Rhizospheric microorganisms can alter plant physiology and morphology in many different ways including through the emission of volatile organic compounds (VOCs). Here we demonstrate that VOCs from beneficial root endophytic *Serendipita* spp. are able to improve the performance of *in vitro* grown *Arabidopsis* seedlings, with an up to 9.3-fold increase in plant biomass. Additional changes in VOC-exposed plants comprised petiole elongation, epidermal cell and leaf area expansion, extension of the lateral root system, enhanced maximum quantum efficiency of photosystem II (F_v_/F_m_), and accumulation of high levels of anthocyanin. Notwithstanding that the magnitude of the effects was highly dependent on the test system and cultivation medium, the volatile blends of each of the examined strains, including the references *S. indica* and *S. williamsii*, exhibited comparable plant growth-promoting activities. By combining different approaches, we provide strong evidence that not only fungal respiratory CO_2_ accumulating in the headspace, but also other volatile compounds contribute to the observed plant responses. Volatile profiling identified methyl benzoate as the most abundant fungal VOC, released especially by *Serendipita* cultures that elicit plant growth promotion. However, under our experimental conditions, application of methyl benzoate as a sole volatile did not affect plant performance, suggesting that other compounds are involved or that the mixture of VOCs, rather than single molecules, accounts for the strong plant responses. Using *Arabidopsis* mutant and reporter lines in some of the major plant hormone signal transduction pathways further revealed the involvement of auxin and cytokinin signaling in *Serendipita* VOC-induced plant growth modulation. Although we are still far from translating the current knowledge into the implementation of *Serendipita* VOCs as biofertilizers and phytostimulants, volatile production is a novel mechanism by which sebacinoid fungi can trigger and control biological processes in plants, which might offer opportunities to address agricultural and environmental problems in the future.

## Introduction

In search of sustainable alternatives for chemical fertilizers and plant protection products, the use of beneficial rhizosphere microorganisms and/or their bioactive compounds as potential biofertilizers, phytostimulants, and biocontrol agents has been widely explored (reviewed, e.g., in Ahmad et al., 2018). In this context, the endophyte *Serendipita indica* (formerly *Piriformospora indica*) and its close relatives within the Serendipitaceae (Sebacinales, Agaricomycetes, Basidiomycota) have attracted attention because of their broad host spectrum and positive influence on diverse aspects of plant development. They are believed to have great potential for applications in sustainable crop production systems (reviewed in Franken, 2012; Ray and Craven, 2016) and in small-scale, resource-limited agriculture in developing countries (Venneman et al., 2019). We have shown, for instance, that *ex vitro* inoculation of several crops with Congolese *Serendipita* strains improves the plants’ capacity to cope with adverse (a)biotic conditions (Venneman, 2020). Interestingly, based on *in vitro* assays with inoculated *Arabidopsis* seedlings, it was also established that the positive plant growth responses emerged prior to physical contact with the Congolese isolates or the reference strains *S. indica* and *S. williamsii* (Venneman et al., 2017), suggesting the involvement of a bioactive diffusible compound. For *S. indica*, it has indeed been demonstrated that *Arabidopsis* root architecture is affected by a diffusible fungal factor whose effects can be mimicked by auxin (Peškan-Berghöfer et al., 2004; Sirrenberg et al., 2007). However, although *S. indica* is able to produce indole-3-acetic acid in liquid culture (Sirrenberg et al., 2007), the improved plant growth appeared to be triggered by a different (unknown) component in the exudates of the fungal hyphae (Lee et al., 2011). Furthermore, Hilbert et al. (2012) showed that the mycelium-synthesized auxin is not required for growth promotion but for biotrophic colonization of barley roots. Similarly, although the *cis*-zeatin- and isopentenyladenine-type cytokinins that are produced by *S. indica* may play an important role in the beneficial interaction with *Arabidopsis*, they are not the elusive bioactive compounds responsible for the observed plant growth effects (Vadassery et al., 2008).

Recently, it has become apparent that diffusible metabolites are not the only microbial products to be considered. Since Ryu and co-workers (2003) provided the first evidence that volatile organic compounds (VOCs) of microbial origin can modulate plant physiological processes as well, these previously unexplored metabolites and their biological functions have gained increasing interest in the field of bioprospecting. Indeed, within an agricultural and horticultural context, these microbial molecules with a low molecular weight, high vapor pressure, low boiling point and a lipophilic nature do not only serve as ideal infochemicals mediating intra- and interspecies communication, but could also be exploited as biocontrol or plant growth-modulating agents (reviewed e.g., in Kanchiswamy et al., 2015; Schulz-Bohm et al., 2017; Garbeva and Weisskopf, 2020). Despite that some microbial VOCs elicit neutral or inhibitory effects on plants (stunted growth, chlorosis, senescence; Lee et al., 2019; Garbeva and Weisskopf, 2020), those VOCs that cause positive physiological changes might hold great promise for future applications as sustainable bioproducts. Although the extent of the observed plant responses depends on the organism under study, typical effects recorded for different plants exposed to bacterial and fungal VOCs comprise cell expansion, increase in shoot biomass, expansion of the root system, improved photosynthetic activity associated with a rise in leaf chlorophyll content and quantum yield of photosystem II (PSII) photochemistry, accumulation of starch and anthocyanin, enhanced nutrient availability from soil, induced systemic resistance, and increased tolerance to abiotic stress (e.g., Ryu et al., 2003; Kishimoto et al., 2007; Zhang et al., 2007; Zhang et al., 2008a; Zhang et al., 2008b; Ezquer et al., 2010; Minerdi et al., 2011; Naznin et al., 2013; Naznin et al., 2014; Bhattacharyya et al., 2015; Bitas et al., 2015; Sánchez-López et al., 2016a). These VOC-induced alterations in plant development usually depend on changes in the transcriptome, metabolome, and/or proteome and are closely linked to the modulation of specific phytohormone signaling pathways (e.g., Kwon et al., 2010; Hao et al., 2016). The occurrence of such conserved plant responses has often been reported for beneficial organisms, such as bacterial species belonging to the genera *Bacillus* (Ryu et al., 2003; Gutiérrez-Luna et al., 2010), *Burkholderia* (Blom et al., 2011; Groenhagen et al., 2013) and *Pseudomonas* (Han et al., 2006; Park et al., 2015), and some fungal species including *Cladosporium cladosporioides* CL-1 (Paul and Park, 2013), *Laccaria bicolor* (Ditengou et al., 2015), *Phoma* sp. GS8-3 (Naznin et al., 2013), *Talaromyces wortmannii *FS2 (Yamagiwa et al., 2011), *Trichoderma atroviride* (Garnica-Vergara et al., 2016), and non-pathogenic *Fusarium oxysporum* MSA 35 (Minerdi et al., 2011). However, it was Sánchez-López et al. (2016a) who truly demonstrated the generality of this microbial capacity to change plant performance through VOC emissions by testing 13 bacterial and 17 fungal phylogenetically diverse isolates, encompassing saprotrophs, beneficials and phytopathogens as well as microbes that normally do not interact with plants. In line with this finding, Camarena-Pozos et al. (2019) recently revealed that approximately 90% of 40 agave- and cactus-associated bacterial strains promoted growth and development of *Arabidopsis* and *Nicotiana benthamiana via* the emission of volatiles.

Our primary observations indicated that plant shoots and roots react to *Serendipita* inoculations well in advance of the establishment of physical contact. Therefore, in the current study, using different experimental setups, we examined the *in vitro* production of VOCs by our Congolese *Serendipita* isolates and compared their effect on morphological and physiological traits of *Arabidopsis* with that of the two reference strains. We evaluated the impact of nutrient availability and hence of fungal metabolism on these plant responses and assessed the relative contribution of fungal respiratory CO_2_ and other volatile compounds to the observed plant growth promotion. We additionally analyzed the composition of the volatile blends of *Serendipita* isolates grown under different conditions in an attempt to identify compounds implicated in the positive plant responses. Finally, using mutants and reporter lines, we assessed the putative role of the main plant hormone pathways in the observed VOC-mediated shoot and root modifications. To conclude, we combined our data with those from other VOC studies to propose a model on this novel mechanism employed by *Serendipita* isolates to alter the development of their host.

## Materials and Methods

### Plant Materials and Growth Conditions

Wild-type *Arabidopsis thaliana* (ecotype Columbia-0 and Wassilewskija), procured from NASC (University of Nottingham, UK), was used in the experiments, unless stated otherwise. An overview of the evaluated mutant and transgenic lines is given in **Supplementary Table S1**.

*Arabidopsis* seeds were surface sterilized (3 min in 70% (v/v) ethanol with 0.05% (v/v) Triton X-100, 10 min in 100% ethanol, and dried under a sterile airflow) and arranged on sucrose‐free half-strength (½) Murashige and Skoog (MS) salts medium including vitamins (M0222; Duchefa Biochemie, Haarlem, The Netherlands), supplemented with 0.5 g L^−1^ MES monohydrate, 0.1 g L^−1^ myo-inositol and 7.0 g L^−1^ Phyto agar (P1003; Duchefa) (pH 5.7). The choice to use sucrose-free MS medium in most of our assays was supported by Sánchez-López et al. (2016a), who pointed out that addition of exogenous sucrose possibly inhibits expression of photosynthetic genes and may induce developmental arrest and leaf senescence in plants. Only in case of the chloroplast mutants, 1% (w/v) sucrose was added to the ½ MS medium. The seeds were vernalized for 3 days at 4°C in the dark and subsequently transferred to a growth chamber at 22°C under a 16/8-h light/dark photoperiod (45 µmol photons m^−2^ s^−1^, 3350 lumen from 36 W cool white fluorescent tungsten tubes). Plates destined for use in experiments studying the aboveground VOC-mediated effects were incubated horizontally (21 seeds per 90-mm diameter Petri dish) and those for analyzing the root effects were placed vertically (3 rows of 7 seeds per 120 mm × 120 mm Petri dish).

### Fungal Strains and Growth Conditions

The Congolese *Serendipita* isolates were previously obtained from sudangrass roots growing in soil-based trap systems (Venneman et al., 2017). The reference isolates *S. indica* and *S. williamsii* were kindly provided by Dr. Karl-Heinz Kogel (Institute of Phytopathology, Justus-Liebig-Universität, Gießen, Germany). The saprophytic fungus *Aspergillus fumigatus* and the pathogenic *Cochliobolus sativus* were part of the laboratory collection.

All fungal isolates were stored as spore suspensions in 15% (v/v) (*Serendipita* spp.) or 20% (v/v) (*A. fumigatus*, *C. sativus*) glycerol at −80°C. Approximately 10 days prior to the start of the volatile assays, the strains were revitalized by inoculation on potato dextrose agar (PDA) and incubated at 25°C (pathogenic/saprophytic fungi) or 30°C (*Serendipita* spp.).

In addition to PDA, the following media were used: plant nutrition medium (PNM), ½ MS without sucrose, ½ MS + 1% (w/v) sucrose, malt yeast peptone (MYP), malt extract agar (MEA) and complex medium (CM) (**Supplementary Table S2**).

### Petri-Dish-in-Box Bioassays

Two 55-mm diameter Petri dishes containing either a fungal or a plant culture were placed side by side without lids in a closed sterilized polypropylene box with airtight cover (540 ml; Eco2 NV, Ophasselt, Belgium) (**Supplementary Figure S1A**). Prior to the start of the co-cultivation, four 12-day-old (including vernalization) *Arabidopsis* seedlings of similar size and growth stage, were transferred to a Petri dish containing 10 ml ½ MS solid medium. Depending on the experiment, the MS medium was supplemented with 0%, 1% or 3% (w/v) sucrose as indicated. Four (*Serendipita* spp., *C. sativus*) or two (*A. fumigatus*) days before the seedling transfer, fungal cultures were initiated by inoculating one plug of a 10-day-old fungal culture (5 mm diameter) on 10 ml PDA or another fungal medium in a separate Petri dish. The control treatment consisted of *Arabidopsis* plants growing adjacent to plates containing only sterile fungal culture medium. Unless otherwise mentioned, at least three replicates were included for each tested condition. The seedlings were exposed to the fungal volatiles for 10 days. The boxes were arranged in a completely randomized design inside the growth chamber with conditions as described above.

At the end of the co-cultivation, images were taken and spectral and chlorophyll fluorescence data were collected using a multispectral plant phenotyping platform. Additionally, the fresh weight (FW) of individual shoots was determined.

### Split-Plate Bioassays

*Serendipita* isolates and *Arabidopsis* seedlings were grown in two different compartments of a round polystyrene Petri dish with a center partition (I‐plates, 90 mm x 14.2 mm; Plastiques Gosselin, Borre, France), which was sealed with plastic foil, unless otherwise specified (**Supplementary Figure S1B**). Co‐cultivation was initiated in a similar way and performed under the same conditions as described for the Petri-dish-in-box assays. Except for the chloroplast mutants that were grown on ½ MS with 1% (w/v) sucrose, all plants were transferred to 10 ml ½ MS without sucrose, while fungi were inoculated on 10 ml PDA or another fungal medium as indicated.

After 10 days (14 days for the chloroplast mutants), pictures were taken and shoot FW determined. For some experiments, leaf series of one representative plant per plate were prepared on 1% (w/v) agar medium and leaf areas were measured using the ImageJ Software (v1.51j8; National Institutes of Health, Bethesda, MD, USA; Schneider et al., 2012). Simultaneously, the fourth leaf of the remaining plants per plate was dissected, cleared in 100% ethanol for 2 days and stored in lactic acid prior to analysis of the epidermal pavement cell size. Abaxial epidermal cells located at 25% and 75% from the leaf base, halfway between the midrib and the leaf margin, were compared between control and VOC-treated leaves on an Olympus IX81 inverted microscope (Olympus, Tokyo, Japan) equipped with optical components for phase contrast microscopy.

### Vertical-Plate Assays

Four 9-day-old (including vernalization), vertically pregerminated *Arabidopsis* seedlings were transferred to a 120 mm x 120 mm square Petri dish filled with 40 ml of sucrose-free ½ MS medium in which a round Petri dish (35 mm diameter, 10 mm high, easy grip; SPL Life Sciences, Pocheon-si, Korea) had been fixed aseptically against the right border of the plate (**Supplementary Figure S1C**). Plants were placed 1.5 cm apart at the left side of the plate. The round plate contained a *Serendipita* culture inoculated on 3 ml of PDA medium, which had been incubated for 3 days at 30°C prior to the start of the experiment. The square plates were sealed with plastic foil and were placed upright and in random order inside a growth chamber under the same conditions as described above. After 4 and 8 days of VOC exposure, shoots and roots were photographed and shoot FW was determined. An in-house developed software tool for automated root phenotyping was used to facilitate the measurement of primary root length, total lateral root length, and lateral root density (Venneman et al., 2017). Three biological replicates for both VOC exposure times were prepared simultaneously. Regarding the 8-day exposure, two additional experiments were conducted with one biological replicate each.

### Quantification of Fungal Respiratory CO_2_

Respiratory CO_2_ produced by *Serendipita* on PDA and on ½ MS without or with sucrose (1%, w/v) and by *A. fumigatus* and *C. sativus* cultures grown on PDA was quantified within a 4-L glass system (**Supplementary Figure S1D**). Two 6-day-old fungal cultures in 55‐mm round Petri dishes were placed inside the reaction vessel. The entire volatile system was placed in a growth chamber with temperature and light conditions identical to those used during the co-cultivation experiments. The in- and outlet of the vessel was blocked for 5 days by means of a perfluoroalkoxy alkane (PFA) plug valve (Swagelok, Solon, OH, USA), after which CO_2_ levels were determined by connecting a MultiRAE Lite multi-gas monitor with pump (RAE Systems, Inc., Sunnyvale, CA, USA) to the opened outlet for 20 s. Four independent measurements were performed for *Serendipita* isolate 30 inoculated on PDA. For isolate 30 cultured on ½ MS without or with sugar and for *A. fumigatus* and *C. sativus* grown on PDA, a single measurement was taken.

### CO_2_ Entrapment and Gas Exchange Experiments

A trapping experiment with round tripartite Petri dishes (Y‐plates, 94 mm x 15 mm; Greiner Bio-One International GmbH, Kremsmünster, Austria; Kai and Piechulla, 2009) was set up according to the above-described split-plate assays with three biological replicates per treatment. Five Col-0 plants were transferred to a compartment filled with 7 ml ½ MS without sucrose and *Serendipita* isolate 30 was grown on either 7 ml PDA or 7 ml ½ MS supplemented with 1% (w/v) sucrose. The third compartment was filled with 7 ml 0.1 M Ba(OH)_2_, which reacts with CO_2_ to form a BaCO_3_ precipitate. After 8 days, shoot FW was determined.

Alternatively, split plates with two compartments were sealed with different types of tape: plastic foil blocking all gas exchange, a polyurethane-based adhesive strip allowing exchange of O_2_ and CO_2_ (Breathe-Easy film, Diversified Biotech, Inc., Boston, MA, USA), and a completely air permeable surgical tape (Micropore, 3M, St. Paul, MN, USA). Per isolate-sealing tape combination, three replicates were included and after 10 days of VOC exposure the Col-0 shoot FW was measured.

### CO_2_ Fertilization

For CO_2_ fertilization, an elevated CO_2_ level was created inside a 4-L glass system (**Supplementary Figure S1D**). In this context, CO_2_-response curves of photosynthesis and biomass production usually reach a point at which further increase in CO_2_ is no longer accompanied by a higher leaf net photosynthetic rate or plant growth. For C3 plants, including *Arabidopsis*, the maximal plant response is typically obtained at a CO_2_ level around 1000 ppm (Xu, 2015; Zheng et al., 2018). With this saturation point in mind and based on CO_2_-fertilization applications in horticulture, 1500 ppm CO_2_ was added to the 4-L glass reaction vessel. The latter contained three 35‐mm round Petri dishes filled with 4 ml ½ MS without sucrose and three 12‐day-old (including vernalization) *Arabidopsis* seedlings. CO_2_ was supplied one to three times a day by connecting a compressed air cylinder containing a 99.85/0.15% N_2_/CO_2_ gas mixture (Air Liquide, Paris, France) to the system until 1500 ppm was reached (**Supplementary Figure S2A**). Subsequently, the external supply was shut off and a closed circuit was formed by attaching the tubing of the glass units to a circulator pump (**Supplementary Figure S2B**). The circulating air passed through a CARBOCAP CO_2_ probe (GMP343; Vaisala, Helsinki, Finland) that was connected to a CR6 data logger (Campbell Scientific, Inc., Logan, UT, USA), recording the sensor signals at 10-s intervals and averaging them every 60 s. All logged data were immediately transmitted to a computer server for real-time monitoring of the CO_2_ levels using the LoggerNet support software (Campbell Scientific).

The control treatment consisted of a closed circuit without the addition of external CO_2_. Furthermore, both a static control and a static *Serendipita* VOC treatment, i.e., without internal circulation through the reaction vessel, were set up. For the latter, two 4-day-old PDA cultures of isolate 30 were placed adjacent to the seedlings. The four vessels were kept in a controlled environment at a constant temperature of 25°C under a 16/8-h light/dark regime with illumination from the top (1570 lumen from two 18 W cool white fluorescent tungsten tubes) and from the sides (1521 lumen from 13 W warm white LED bulbs). After 7 days of treatment, anthocyanin (AriIdx) and chlorophyll content (ChlIdx), as well as the maximum quantum efficiency of PSII (F_v_/F_m_), were measured in the shoots using a multispectral plant phenotyping platform. Thereafter, shoot FW was determined.

### Volatile Profiling by PTR-TOF-MS Headspace Analysis

For the identification of putative plant growth-promoting volatiles, the headspace composition of *Serendipita* cultures in 4-L glass vessels (**Supplementary Figure S1D**) was analyzed. The in- and outlet, positioned on opposite sides of the system, were fitted to PFA tubing capped with PFA unions and plugs (Swagelok) to close or open the system. To take into account that plant-derived VOCs might affect fungal VOC emission, *Arabidopsis* seedlings were placed adjacent to the fungal cultures (two plates of each). Controls consisted of two fungal plates combined with two plates containing sterile plant medium (fungal control) and two plant cultures placed beside two plates with non-inoculated fungal growth medium (plant control). For *Serendipita*, 4-day-old cultures grown on 12 ml PDA (isolate 30, *S. indica* and *S. williamsii*) or 12 ml ½ MS without sucrose (isolate 30) were used. For *Arabidopsis*, 12‐day-old (including vernalization) Col-0 seedlings were transferred to 7 ml sucrose-free ½ MS medium (four plants per plate). The closed glass vessels were placed in a controlled growth chamber at 22°C under a 16/8-h light/dark cycle. After 4 days of co-cultivation, headspace sampling and analysis was performed using both Proton Transfer Reaction-Time of Flight Mass Spectrometry (PTR-TOF MS) and Thermal Desorption-Gas Chromatography Mass Spectrometry (TD-GC MS) (**Supplementary Materials and Methods**). Three independent measurements were carried out for isolate 30 (on PDA), whereas *S. indica* and *S. williamsii* (and isolate 30 on ½ MS) were evaluated in a single experiment.

Each PTR measurement lasted 45 min (2,700 spectra/cycles per sample) and was preceded by a 3-min analysis of the N_2_ stream for background level recording. Between measurements, tubing and transfer line were flushed with N_2_ gas to avoid carry over. All PTR-TOF MS raw data with ion signal intensities expressed in counts per second were recorded by the TofDAQ Viewer v1.2.99 (Tofwerk AG, Thun, Switzerland) and the resulting data files were post-processed using PTR-MS Viewer v3.2.8 (IONICON Analytik GmbH, Innsbruck, Austria) (**Supplementary Materials and Methods**). For every compound with a mass-to-charge (m/z) ratio between 15.993 and 300.066, the retrieved concentrations in parts per billion (ppb) from the first 60 cycles of the sample signal (excluding a short stabilization period of 20 cycles immediately after the start of the measurements) were averaged, followed by the subtraction of the background signal, which was obtained by averaging the concentrations from the last 60 cycles of the N_2_ gas analysis. Then, compounds with net negative concentrations were removed from the data set, as well as primary ions, PerMaSCal gas molecules, known compounds with a proton affinity smaller than that of water (such as CO_2_, NO_2_, etc.), and constituents with concentrations below the limit of quantification, defined as ten times the standard deviation of the background noise (Deuscher et al., 2019) which was calculated for the last 60 s of VOC-free N_2_ supply.

To evaluate differences between the volatile profiles, a principal component analysis (PCA) was conducted after applying mean-centering and unit variance scaling to the data of the retained volatile compounds, using the *dudi.pca* function in the *ade4* package of R (Dray et al., 2007). The PCA output was visualized with the ggplot2-based functions *fviz_pca_ind*, *fviz_pca_var*, and *fviz_pca_biplot*, available within the *factoextra* package (Kassambara and Mundt, 2017). To determine which compounds were most strongly associated with the fungal treatments, those with a cos2 (square cosine, squared coordinates) value below 0.25 on dimension 1 and 2 were discarded as a first variable-reducing step. The closer the sum of the cos2 on dimension 1 and 2 is to one, the better a variable is represented by these two principal components. Based on their orientation in the reduced X/Y coordinate system, only those compounds that seemed to be linked to the fungal treatments were retained and examined more closely with respect to their average signal. Volatiles showing a steady rise in concentration or maintaining a constant level, instead of displaying a decay after a certain period of time, were not considered.

The VOCs that seemed most promising after this multivariate analysis were tentatively identified by inspecting their measured protonated mass and their isotopic distribution. For a final identification of these compounds, their MS spectra obtained from the TD-GC-MS analysis were compared with the NIST Mass Spectral Library (Gaithersburg, MD, USA) and their retention times were compared with those of reference standards that were run under the same conditions as the samples.

### Methyl Benzoate Treatment

Methyl benzoate (Acros Organics, Geel, Belgium) was added to water in 540-ml polypropylene boxes and 500-ml Duran glass bottles with a wide neck (GLS 80; Duran, DWK Life Sciences, Mainz, Germany) (**Supplementary Figure S1E**). In these setups, a plate containing 5 ml sterile distilled water was spiked with either 10 µL or 100 µL of a freshly prepared 0.4-mM methyl benzoate stock solution in HPLC-grade water and placed beside three 35‐mm round Petri dishes filled with 5 ml of ½ MS medium without sucrose or supplemented with 1% or 3% (w/v) sucrose, with each three 12-day-old *Arabidopsis* seedlings (including vernalization). In the control containers, plants were grown adjacent to plates containing untreated sterile water. Each condition was examined once, in a growth chamber at 22°C under a 16/8-h light/dark regime. The methyl benzoate level over the time course of the experiment was monitored daily in a separate Duran flask (without plants), which was fitted with a PTFE GLS 80 bottle multi-dispenser (DWK Life Sciences), facilitating the injection of headspace air into the PTR-QiTOF instrument.

In a second assay, a gas-generation system was developed to supply a 4-L glass vessel containing *Arabidopsis* plants with a continuous flow of methyl-benzoate-enriched air. A separate trajectory was installed for the control, in which the flow passing by the plants did not carry methyl benzoate (**Supplementary Figure S3**). Plants were grown on ½ MS without and with 3% (w/v) sucrose and three plates of each were placed in the same vessel. The experiment, which was carried out once, was installed in a controlled room at 22°C with illumination from the top and the sides under a 16/8-h light/dark regime. Prior to the start of the volatile treatment, the methyl benzoate concentration was stabilized at a concentration of ca. 20 ppb by means of a regulatory valve inserted inside the system.

After 7 days, the shoot FW was measured.

### Multispectral Plant Phenotyping

For several experiments, standardized spectral measurements of anthocyanin and chlorophyll content in the *Arabidopsis* shoots were performed (indices AriIdx and ChlIdx, respectively), and the chlorophyll fluorescence parameter F_v_/F_m_, related to the maximum quantum efficiency of PSII photochemistry, was determined after dark adaptation. The AriIdx was calculated according to the formula of the modified anthocyanin reflectance index (mARI) (Gitelson et al., 2006). All data were generated on an automated plant phenotyping platform, installed within a controlled laboratory environment. The platform is a customized WIWAM XY system (SMO, Eeklo, Belgium) that is equipped with a 6-Mp 16-bit 3CCD top-view camera (PhenoVation Life Sciences, Wageningen, The Netherlands) mounted on a Cartesian coordinate robot for high-throughput and high-resolution RGB, fluorescence and multispectral imaging. The AriIdx, ChlIdx and F_v_/F_m_ parameters were assessed with respect to the distribution of the recorded pixels across different classes having custom-defined value ranges using the accompanying CropReporter Data Analysis software of PhenoVation. Per analyzed sample and per parameter, the software calculated how many pixels were assigned to each class (expressed relatively).

### Histochemical Analysis of Reporter Lines

Exposed and control seedlings of the *CYCB1;1::GUS*, *DR5::GUS*, and *ARR5::GUS* reporter lines were analyzed for *in planta* GUS (β-glucuronidase) activity by histochemical staining (Jefferson et al., 1987). Seedlings were fixed in 90% (v/v) ice-cold acetone for 30 min, washed twice in 100 mM sodium phosphate buffer (pH 7.0), and immersed in GUS solution (1 mM X-gluc (5-bromo-4-chloro-3-indolyl-ß-d-glucuronic acid; Carl Roth, Karlsruhe, Germany), 100 mM sodium phosphate buffer (pH 7.0; Carl Roth), 0.1 mM K_4_Fe(CN)_6_ (Sigma-Aldrich, St. Louis, MO, USA), 0.1 mM K_3_Fe(CN)_6_ (Sigma-Aldrich), 0.1% (v/v) Triton X-100 (Sigma-Aldrich)) for 2 h at 37°C in the dark. After the 2‐h incubation period, the chlorophyll of the seedlings was extracted with 70% (v/v) ethanol, after which they were preserved at 4°C in 70% ethanol until examination. GUS expression patterns were observed and recorded using an Olympus SZX16 stereomicroscope equipped with an Olympus XC50 digital camera. The number of established lateral roots and lateral root initiation sites per cm primary root of *CYCB1;1::GUS* seedlings was determined for representative plants of the control and the VOC treatment with *Serendipita* isolate 30.

### Statistical Analyses

All statistical tests (α = 0.05) were done in RStudio, R version 3.5.1 (R Core Team, 2019). Graphs were constructed in either RStudio or SigmaPlot v13 (Systat Software, Inc., San Jose, CA, USA). The data of each experiment were first checked for normality of the response variable (Shapiro-Wilk test in package *RVAideMemoire*; Hervé, 2018) and homoscedasticity of variances (Levene’s test in *car*; Fox and Weisberg, 2011). If the normality and homoscedasticity assumptions were met, a Student’s t-test or a one-way analysis of variance (ANOVA) was performed (in *stats*; R Core Team, 2019). In case significant differences were detected, the latter was followed by a Tukey’s HSD test for multiple comparisons (in *agricolae*; de Mendiburu, 2019) or by a Dunnett’s test for comparing all treatments specifically with the control (in *DescTools*; Signorell et al., 2019). The used non-parametric alternative to ANOVA and Tukey’s *post hoc* analysis consisted of a Kruskal-Wallis test (in *stats*), followed by a Dunn’s test adjusted for multiple comparisons through a Bonferroni correction (in *FSA*; Ogle et al., 2018).

## Results

### Volatile Compounds Produced by *Serendipita* Isolates Strongly Enhance *Arabidopsis* Shoot and Root Growth

**Figure 1:**
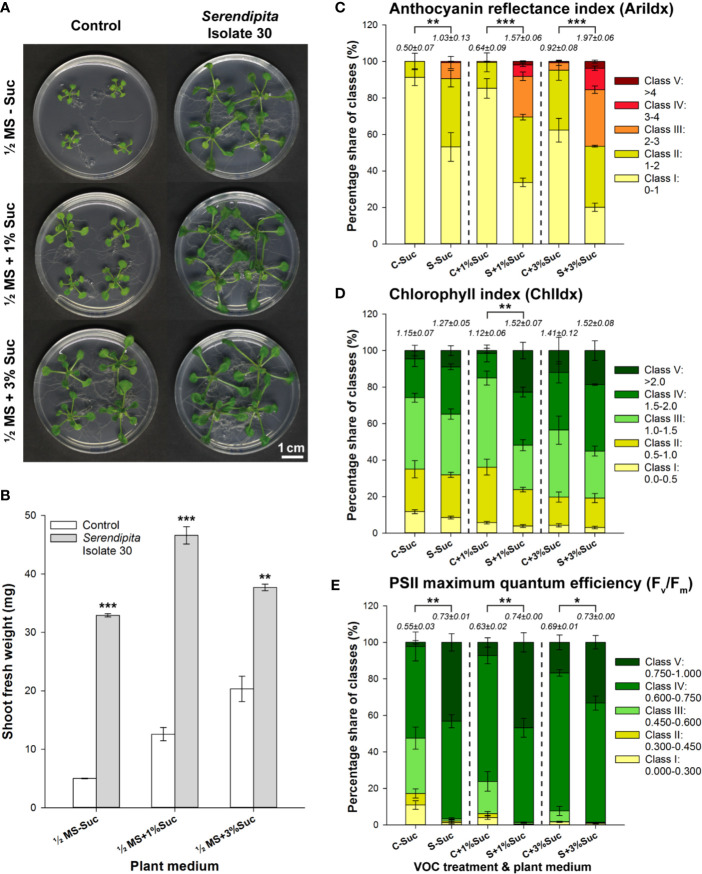
Effect of volatile production by PDA-grown *Serendipita* isolate 30 on *A. thaliana* grown on ½ MS with 0%, 1% and 3% sucrose, in a Petri-dish-in-box-assay. **(A) **Plant growth promotion after 10 days of co-cultivation. **(B) **Increase in mean shoot fresh weight. **(C–E) **Distribution of pixels across different classes of the spectral parameters anthocyanin (AriIdx, **C**) and chlorophyll (ChlIdx, **D**) content, and of the chlorophyll fluorescence parameter F_v_/F_m_ **(E)**. Average total values (± standard error) of the different spectral and fluorescence parameters for each of the tested conditions are shown above the respective stacked bars. For a visualization of the pixel distribution across the different classes in the shoot see **Supplementary Figure S4**. Error bars in **(B–E)** represent standard errors on the mean of four biological replicates with each replicate consisting of four plants. Asterisks indicate significant differences compared with the control values according to one-sided t-tests (* < 0.05, ** < 0.01, *** < 0.001). C, Control; MS, Murashige and Skoog; S, *Serendipita*; Suc, sucrose.

To assess the impact of *Serendipita* volatile production on *Arabidopsis*, a Petri-dish-in-box co‐cultivation assay was established (**Supplementary Figure S1A**) in which the fungus was cultured on PDA while the seedlings were grown on ½ MS medium supplemented with sucrose (0%, 1% and 3%). Isolate 30 was chosen as test organism because of its high growth rate and its strong plant growth-promoting (PGP) abilities in previous direct contact assays (Venneman et al., 2017). After 10 days of co-cultivation, a clear induction of shoot and root growth was observed in the VOC-treated plants, but the response relative to the control without fungus became less pronounced as the sucrose level in the plant medium increased (**Figure 1A**). A 6.6-, 3.7-, and 1.9-fold rise in shoot FW was recorded on MS medium containing 0%, 1% and 3% sucrose, respectively (**Figure 1B**), constituting a significant decrease in the extent of the growth promotion with improved plant nutrient availability (t‐tests 1% versus 0% and 3% versus 1%, P < 0.001), mainly due to a sugar-driven stimulatory effect in the controls (t‐tests, P < 0.05). Additionally, the petioles of VOC-treated plants were longer and their leaves appeared more robust (i.e., thicker/stiffer) and darker (**Figure 1A**). Multispectral imaging revealed that this darker color was attributed to anthocyanin pigment accumulation (**Figure 1C**), rather than to an increased chlorophyll content (**Figure 1D**). In the VOC-exposed plants, more pixels were assigned to the higher classes of the modified anthocyanin reflectance index (AriIdx parameter), resulting in total values that were at least two times higher than those measured in the controls. The spatial pattern of anthocyanin accumulation revealed a higher concentration in both mature and younger leaves (**Supplementary Figure S4**). Although chlorophyll index values (ChlIdx) were not strongly affected by the fungal treatments, the maximum quantum efficiency of PSII photochemistry, measured in dark-adapted plants *via* the chlorophyll fluorescence parameter F_v_/F_m_, was significantly improved by the fungal VOCs (**Figure 1E**). Imaging of F_v_/F_m_ showed that the elevated levels in exposed plants could at least partly be explained by a higher maximum efficiency of PSII in fully expanded, older leaves (**Supplementary Figure S4**). *Serendipita* VOCs increased the total F_v_/F_m_ values by 33%, 17% and 6% when seedlings were grown on ½ MS supplemented with 0%, 1% and 3% sucrose, respectively. Considering the overall average value of 0.73 to 0.74 across all fungal treatments, this significant sucrose-dependent decrease (t‐tests, P < 0.01) was mainly driven by a nutrient source-linked rise in F_v_/F_m_ values in the controls (t‐tests, P < 0.05). Since the largest plant effects were observed on medium without sucrose, and supported by the findings of Sánchez-López et al. (2016a), this was chosen as the standard plant medium in subsequent experiments.

**Figure 2:**
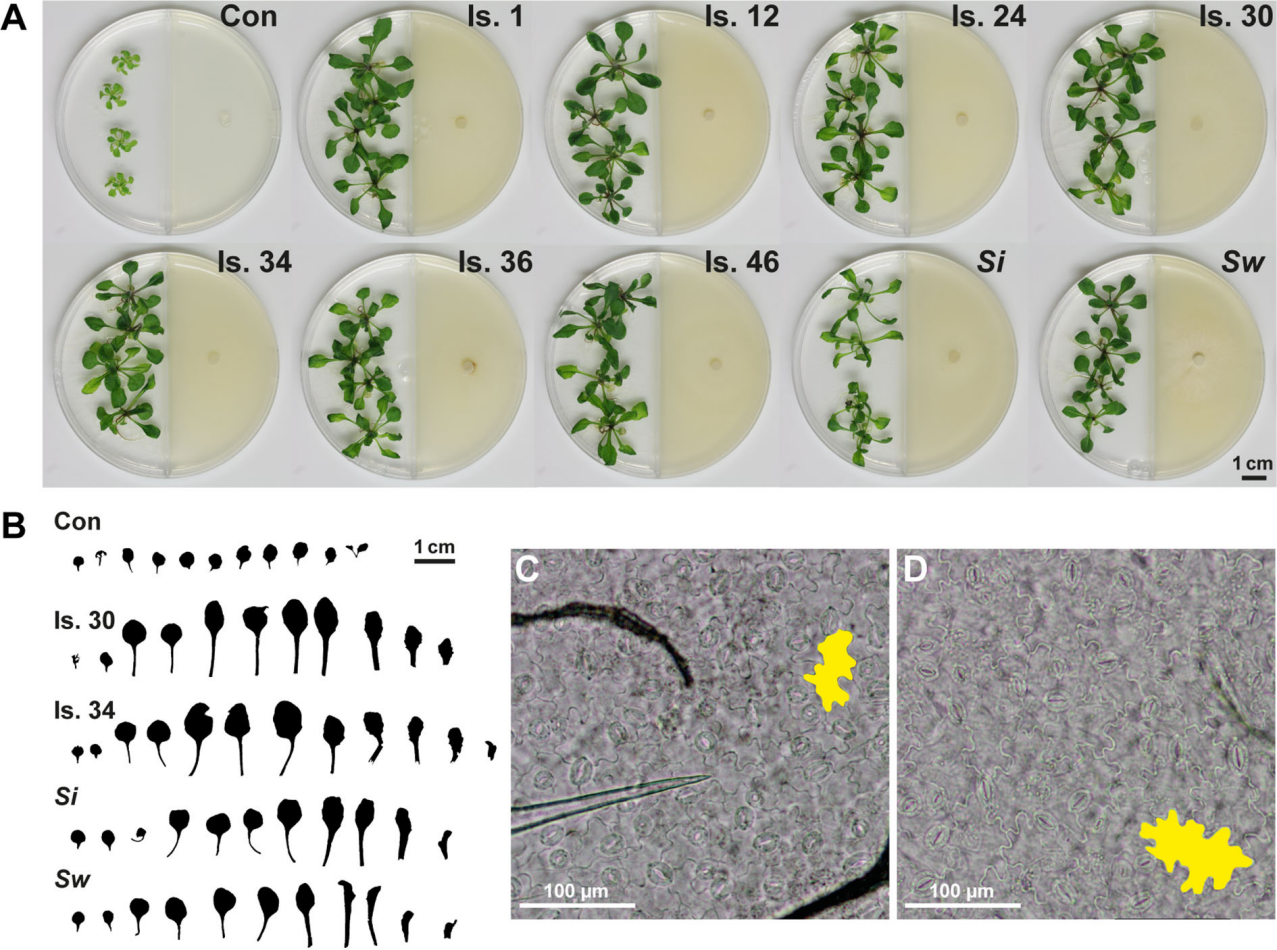
VOC-mediated effects of *Serendipita* on *A. thaliana* growth in split-plate assays. **(A)** VOC-driven plant growth effects (on ½ MS without sucrose) for a representative subset of PDA-grown *Serendipita* isolates after 10 days of co-cultivation. **(B)** Leaf series visualizing differences in leaf surface area and petiole length between control and VOC-treated plants. **(C)** Epidermal pavement cells as observed by light microscopy on the abaxial side of the fourth leaf of a control plant. **(D)** Same as **(C)** but with the leaf originating from a VOC-exposed plant. Data on the impact of *Serendipita* VOCs on *Arabidopsis* shoot biomass are presented in **Supplementary Figures S5** and **S9**. Con, Control; Is., Isolate; *Si*, *Serendipita indica*; *Sw*, *Serendipita williamsii*.

We next investigated whether other *Serendipita* isolates might provoke different plant growth responses. Therefore, the collection of 51 Congolese isolates (Venneman et al., 2017) and the reference strains *S. indica* and *S. williamsii* were screened in I‐plate assays. Compared with the control treatment, all *Serendipita* isolates induced an increase in *Arabidopsis* shoot biomass, varying between 5‐ and 9.3‐fold along a continuous range (**Supplementary Figure S5**). Then, leaf series were prepared for individual plants exposed to VOCs from *S. indica*, *S. williamsii* and one representative member of each of the seven previously defined “genetic groups” within the Congolese collection (**Figure 2A**) that were used in our reported direct-contact experiments (Venneman et al., 2017). As shown in **Figure 2B**, the increase in total shoot biomass was the combined result of petiole elongation and leaf area expansion (up to 3.7-fold). The abaxial epidermal pavement cells in VOC-exposed plants were at least two times larger compared with those in untreated plants (**Figures 2C, D**), implying that the leaf area expansion can largely be ascribed to cell enlargement rather than to an increased cell number. Additionally, during the preparation of the leaves for this analysis, involving clearing with ethanol and lactic acid, it became apparent that the VOC-treated plants were much darker due to a high concentration of granules, most likely representing starch (**Supplementary Figure S6**).

The impact of the *Serendipita* VOC blends on *Arabidopsis* growth and AriIdx, ChlIdx and F_v_/F_m_ was validated in a Petri-dish-in-box setup (**Supplementary Figure S7**). Of the three examined parameters, the F_v_/F_m_ ratio was most strongly influenced signifying that VOCs from all isolates significantly enhanced the maximum quantum efficiency of PSII (**Supplementary Figure S7B**). Total average values in *Serendipita-*treated plants ranged between 0.71 and 0.74, as compared to 0.61 for the control, with no significant differences among the isolates. Anthocyanin accumulated in plants co-cultivated with isolates 24, 30, 34, 36, 46 and *S. indica* (**Supplementary Figure S7C**). Overall, no significant differences were observed among the *Serendipita* isolates, with indices ranging between 1.5 and 1.9 times the control value. Furthermore, except for isolates 34 and 36, *Serendipita* VOC treatments did not lead to a higher chlorophyll index than the value recorded in the control (**Supplementary Figure S7D**).

**Figure 3:**
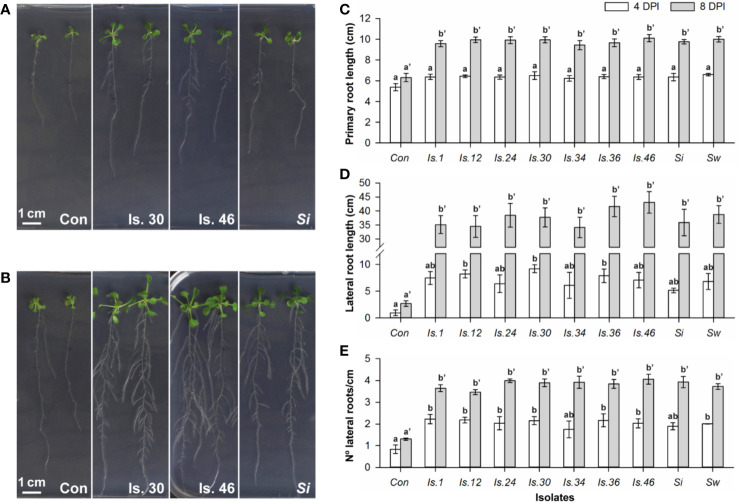
VOC-mediated effects on *Arabidopsis* root development, examined in a vertical setup using a selection of *Serendipita* isolates. **(A, B) **Root and shoot growth stimulation of plants grown on ½ MS without sucrose after four **(A)** and eight **(B)** days of co-cultivation with isolates 30, 46 and *S. indica* cultured on PDA. Photographs of the entire plates for all tested isolates are included in **Supplementary Figure S8**. **(C–E)** Average primary root length **(C)**, total lateral root length **(D)**, and lateral root density (number of lateral roots per cm of primary root; **E**), measured per plant after 4 and 8 days of VOC exposure. Error bars indicate standard errors on the mean of the different treatments, based on three (4 DPI) or five (8 DPI) replicates. For each parameter and for both evaluation moments, values were compared across the different treatments using a Tukey HSD *post hoc* test (α = 0.05); treatments sharing the same letter above their respective bars do not exhibit statistically significant differences. The corresponding data on total root length (primary plus lateral roots) and shoot fresh weight are given in **Supplementary Figures S9** and **S10**. Con, Control; DPI, days post “inoculation” (start of co-cultivation); Is., Isolate; N°, Number; *Si*, *Serendipita indica*; *Sw*, *Serendipita williamsii*.

To view the modulation of root architecture, a vertical plate setup was used (**Supplementary Figure S1C**). Evaluation was done after 4 and 8 days of co-cultivation (**Figures 3A, B**; **Supplementary Figure S8**). After 4 days, compared to the untreated controls, the *Serendipita* VOCs caused a maximum increase of 1.2‐fold (*S. williamsii*) in primary root length (**Figure 3C**), 9.6‐fold (isolate 30) in lateral root length (**Figure 3D**), and 2.7‐fold (isolate 1) in lateral root density (**Figure 3E**), resulting in an up to 2.4-fold increase in total root length (isolate 30; **Supplementary Figure S9**). At the end of the experiment, in the treated plants compared to the controls, the primary, lateral, and total root lengths were, respectively, up to 1.6, 16.1, and 5.7 times longer, and the lateral root density was 3.1 times higher (isolate 46). No significant differences were detected among the tested strains and the variation in the extent of the positive root effect across isolates was comparable to the trend in shoot biomass enhancement (**Figures 3C–E**; **Supplementary Figure S9**).

Altogether, these results show that exposure of *Arabidopsis* to *Serendipita* VOCs induces robust plant responses across different experimental setups, although quantitative differences were observed, illustrating that the output is condition-dependent (**Figure S10**).

### Alterations in the *Serendipita* Metabolism Have an Impact on the Extent of VOC-Induced Growth Promotion

**Figure 4:**
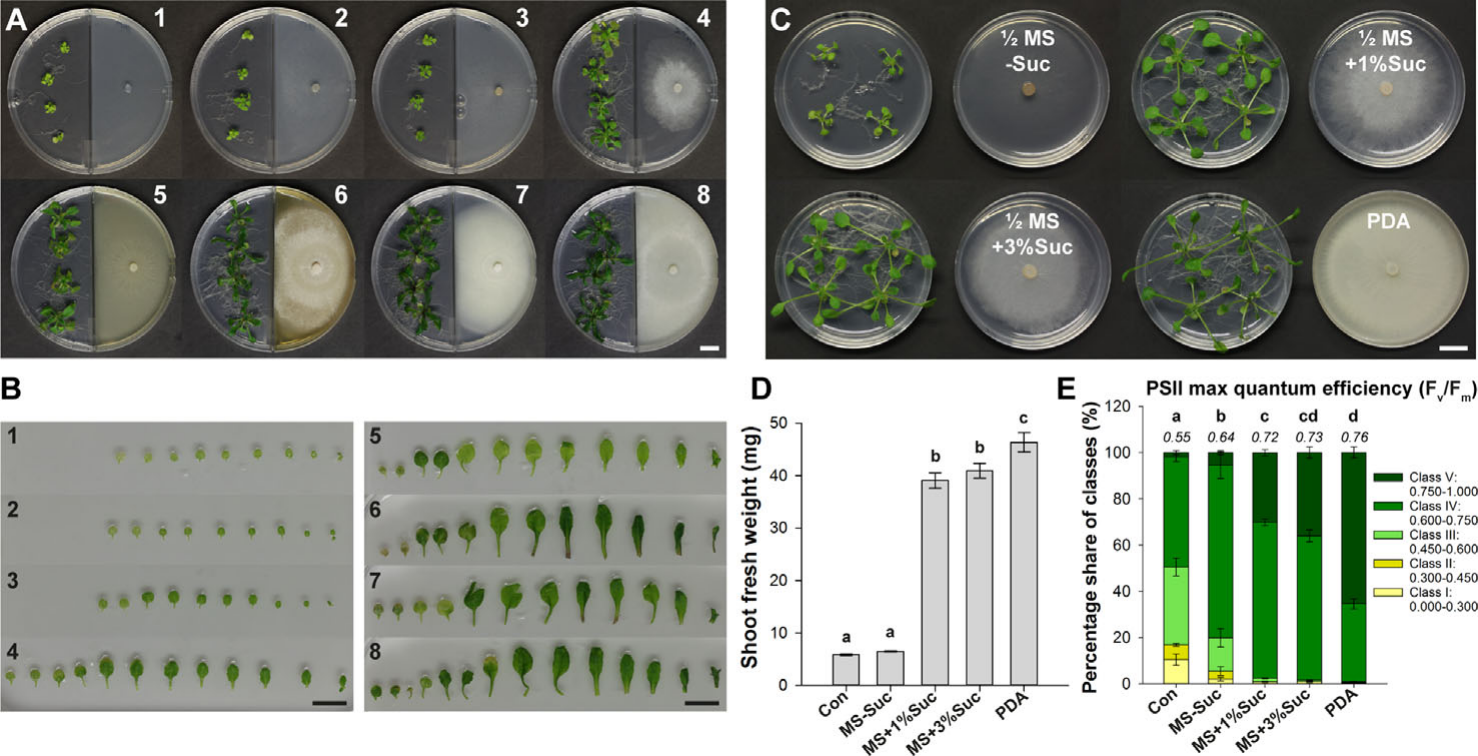
The degree of VOC-mediated plant growth promotion in *Arabidopsis* depends on the *Serendipita* culture medium. **(A)** *Arabidopsis* plant growth (on ½ MS without sucrose) after 10 days of co-cultivation with *Serendipita* isolate 30, grown on different nutrient sources in the right compartment of I‐plates: 1. ½ MS + 1% sucrose (control plate), 2. plant nutrition medium (PNM), 3. ½ MS without sucrose, 4. ½ MS + 1% sucrose, 5. malt yeast peptone (MYP), 6. malt extract agar (MEA), 7. complex medium (CM), 8. potato dextrose agar (PDA). Plates 2–8 are inoculated with *Serendipita* whereas plate 1 is not. **(B)** Leaf series of a representative plant from each of the different growth conditions as presented in **(A)**. **(C)** Plant growth effects caused by a 10-day exposure to *Serendipita* volatiles in a Petri-dish-in-box assay using isolate 30 grown on PDA and on ½ MS medium with 0%, 1% and 3% sucrose. **(D)** Mean shoot fresh weight of plants from **(C)**. **(E)** Pixel distribution across five F_v_/F_m_ classes based on chlorophyll fluorescence imaging of plant shoots from the Petri-dish-in-box assay **(C)**. Average total values of F_v_/F_m_ for each of the tested fungal media are shown above the respective stacked bars (standard errors are very low, ≤ 0.02). The results of the spectral measurements of anthocyanin and chlorophyll content are included in **Supplementary Figure S11**. Error bars in **(D, E)** indicate standard errors on the mean of four biological replicates; treatments sharing the same letter do not show statistically significant differences according to Tukey HSD *post hoc* tests (α = 0.05). Con, Control; MS, ½ Murashige and Skoog medium; Suc, Sucrose; PDA, potato dextrose agar. Scale bars = 1 cm.

Next, we evaluated the effect of VOC blends emitted by representative *Serendipita* isolate 30 cultured on seven different media in I‐split‐plate assays to establish whether the metabolic activity of the fungus affects the outcome of the co-cultivation. Four fungal media (MYP, MEA, CM, PDA) and three plant media containing less nutrients used in previous studies (Johnson et al., 2011; Venneman et al., 2017; PNM, ½ MS without sucrose, ½ MS supplemented with 1% sucrose) were tested. After 10 days of VOC exposure, the strongest impact on shoot and root growth was recorded when the fungus was cultured on MEA, CM or PDA. Additionally, with these media, more robust and darker green plants were obtained (**Figure 4A**). Fungal growth on PNM or ½ MS without sucrose hardly modulated plant development, whereas growth on ½ MS with 1% sucrose or MYP induced an intermediate response (**Figure 4A**). Leaf series from representative plants exposed to the volatiles of MEA, CM and PDA cultures showed dark leaves with long petioles and a large leaf surface area (**Figure 4B**). Although treatment with volatiles generated on ½ MS with 1% sucrose or on MYP resulted in pale-green leaves with intermediate petioles and surface areas approximately 1.25‐ to 1.5‐fold smaller than those on the richer media, on both these media extra leaves developed compared to the control. Finally, growth on PNM or ½ MS without sucrose led to pale, short-petioled, small leaves that were not different from the control leaves (**Figure 4B**).

These observations indicated that volatile emission and the concomitant plant growth promotion was determined by nutrient availability and thus by fungal metabolic activity. Indeed, the extent of the PGP effect was positively correlated with the extent of fungal growth: the mycelial mats produced on the richest media (MEA, CM, PDA), causing the strongest effects, were more dense than those growing on MYP and ½ MS with 1% sucrose, yielding an intermediate effect, and several times thicker than those on the nutrient-poor media (PNM, ½ MS without sucrose), which did not induce any response (**Figure 4A**).

Comparable results were obtained in a Petri-dish-in-box experimental setup when isolate 30 was grown on ½ MS without or with 1% or 3% sucrose, or on PDA (**Figure 4C**). After 10 days of co-cultivation, a 1.1-, 6.6-, 7.0-, and 7.9-fold increase in shoot biomass was measured, respectively (**Figure 4D**). Moreover, the F_v_/F_m_ ratios of all treatments were higher than the control values, but for the treatments with the three richest media, ratios were higher than those recorded for fungal growth on ½ MS without sucrose (**Figure 4E**). Similarly, anthocyanin and chlorophyll contents were higher in plants co-cultivated with isolate 30 grown on PDA and ½ MS containing sucrose (1% and 3%) compared to fungal growth on sugar-free medium and the untreated controls (**Supplementary Figures S11A, B**).

### *Serendipita*-Induced Plant Growth Promotion Is the Combined Result of Fungal Respiratory CO_2_ and Other Volatile Compounds

Given the above results, we assessed whether fungal respiratory CO_2_ accumulated in the closed experimental systems, and if so, what was its contribution to the observed plant growth promotion. The CO_2_ level was determined in the headspace of cultures of isolate 30 grown on PDA and ½ MS without or with 1% sucrose, placed inside a closed 4‐L glass vessel (**Supplementary Figure S1D**) and incubated in a growth chamber under the same conditions as used during the co-cultivations. After 5 days, a MultiRAE Lite multi-gas monitor was connected to the opened outlet of the system and headspace air was pumped into the CO_2_ sensor for quantification. The concentrations measured in the vessel containing cultures grown on ½ MS without sucrose did not exceed 400 ppm, indicating that normal atmospheric levels were retained. On the other hand, CO_2_ concentrations ranging between 7000 and 13 000 ppm were recorded in the vessels with PDA cultures (an average of 10 300 ppm across four independent measurements), signifying a 17.5- to 32.5-fold increase of standard CO_2_ levels. When cultured on ½ MS with 1% sucrose, an intermediate CO_2_ level of 1300 ppm was reached.

**Figure 5:**
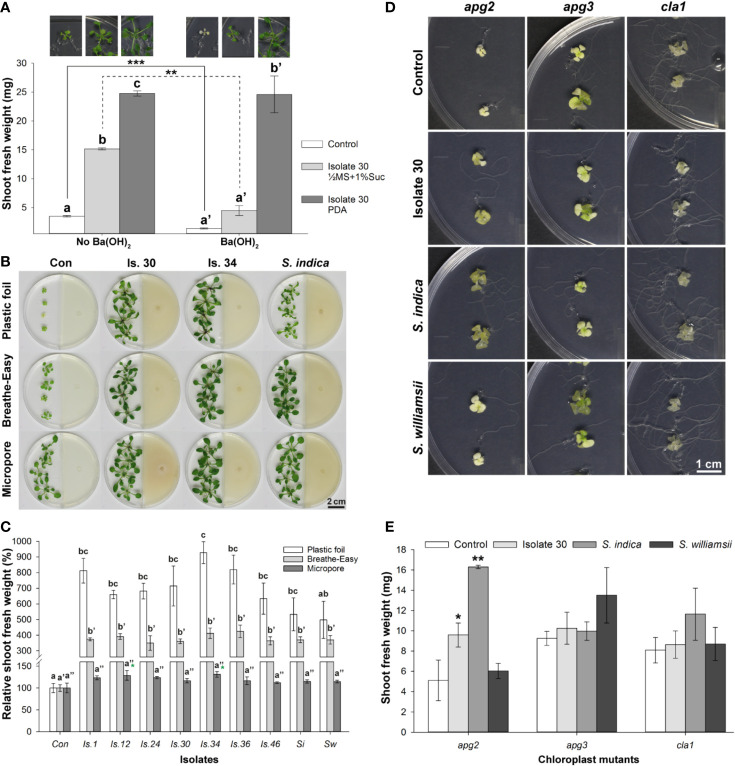
Volatile assays with *Arabidopsis* to assess the contribution of *Serendipita* respiratory CO_2_ to plant growth stimulation and morphology. **(A)** Plant growth and mean shoot fresh weight of Col-0 plants grown on ½ MS without sucrose after 8 days of co-cultivation with *Serendipita* isolate 30 cultured on PDA and on ½ MS with 1% sucrose in Y-plates in the presence and absence of 7 ml 0.1 M Ba(OH)_2_ as a CO_2_ trap. Full images of the different tested conditions can be found in **Supplementary Figures S12A, B**. **(B) ***Arabidopsis* plant growth (on ½ MS without sucrose) as observed after 10 days of co-cultivation with *Serendipita* isolates 30, 34, and *S. indica* (on PDA) in plates sealed with plastic foil, Breathe-Easy strips and Micropore tape. **(C) **Comparison of the average relative shoot fresh weight of *Arabidopsis* plants from plates closed with the sealing tapes from **(B)**. The plastic foil data are derived from four independent experiments with one biological replicate each (see also **Supplementary Figure S10**), while the Breathe-Easy and Micropore results are based on a single experiment with three biological replicates. **(D)** Plant growth responses of the chloroplast mutants *apg2*, *apg3*, and *cla1* grown on ½ MS + 1% sucrose after 14 days of exposure to volatiles from PDA-grown *Serendipita* isolate 30, *S. indica* and *S. williamsii* in I‐plates. Note that not only shoots but also roots in *apg2* mutants are better developed under influence of *Serendipita* volatiles. Photographs of the entire plates are shown in **Supplementary Figure S12C**. **(E)** Mean shoot fresh weights corresponding to the treatments presented in **(D)**. Error bars in **(A, C, E)** indicate standard errors on the mean of three replicates (four in case of plastic foil sealing in **C**). Asterisks in **(A, E)** point to significant differences between indicated treatments **(A)** or compared with the control **(E)** according to one-sided t-tests (* < 0.05, ** < 0.01, *** < 0.001). Treatments sharing the same letter above their respective bars in **(A, C)** are not significantly different according to Tukey HSD *post hoc* tests (α = 0.05). Note that although this test did not detect differences among the isolates in the assay with Micropore tape **(C)**, a one-sided Dunnett’s test on the same data, comparing each treatment specifically with the control, revealed that exposure to VOCs from isolates 12 and 34 did lead to a significantly higher shoot biomass than that recorded for the corresponding control (see green asterisks; * < 0.05). Con, Control; Is., Isolate; MS, Murashige and Skoog medium; PDA, potato dextrose agar; *Si*, *Serendipita indica*; Suc, Sucrose; *Sw*, *Serendipita williamsii*.

Then, three different approaches were taken to evaluate the contribution of *Serendipita* respiratory CO_2_ to the enhanced shoot growth. First, in Y‐plate assays with isolate 30, Ba(OH)_2_ was used to remove headspace CO_2_ through the precipitation of BaCO_3_. After 8 days, untreated *Arabidopsis* plants grown on sucrose‐free ½ MS in the presence of Ba(OH)_2_ had yellow to white leaves (**Supplementary Figure S12A**) and showed a 2.5-fold reduction in average shoot FW compared with control seedlings grown in the absence of the CO_2_ trap (1.4 mg versus 3.5 mg) (**Figure 5A**). Cocultivation of *Arabidopsis* with isolate 30 grown on PDA resulted in a 7.1-fold increase in shoot biomass (24.8 mg versus control weight of 3.5 mg), which was not reduced by adding Ba(OH)_2_ (24.6 mg versus 1.4 mg control weight). Although this result might suggest that respiratory CO_2_ is not implicated in the VOC-mediated plant growth stimulation, given the extremely high CO_2_ levels produced by *Serendipita* on PDA, we considered the possibility that the Ba(OH)_2_ solution could become saturated enabling CO_2_ accumulation in the headspace after all. To test this hypothesis, isolate 30 was grown on ½ MS with 1% sucrose on which it emits five times less CO_2_. In the absence of Ba(OH)_2_, a significant growth promotion was obtained with these cultures, which was less pronounced than with PDA cultures (4.3‐fold versus 7.1‐fold increase in biomass) (**Figure 5A**; **Supplementary Figure S12A**). Importantly, under CO_2_-deprived conditions, a 3.2-fold shoot biomass enhancement could still be discerned under influence of the fungal volatiles (4.5 mg versus 1.4 mg control weight), but the recorded FW was 3.4 times lower compared to VOC exposure under conditions without Ba(OH)_2_ (4.5 mg versus 15.2 mg without CO_2_ trap). These findings indicate that besides respiratory CO_2_, which has a major impact, also other molecules in the volatile blends contribute to the observed plant growth effects.

Secondly, the impact of gas exchange on plant development was examined in an I‐plate-setup in which the plastic sealing foil used in previous assays was replaced by the more permeable Breathe-Easy strip or Micropore tape. Breathe-Easy strips allow an even exchange of O_2_ and CO_2_, while Micropore ensures the free passage of all volatiles, thus preventing CO_2_ accumulation. For the untreated controls, plastic foil had a clear negative impact on shoot and root growth compared to the other two seals (**Figure 5B**). Notwithstanding an improved growth of control plants when using Breathe-Easy and Micropore, exposure to fungal VOCs still yielded larger, darker and more robust plants with an increased biomass, though the magnitude of the response was lower than with plastic foil sealing (**Figure 5B**): the increase in shoot FW was approximately halved when plates were closed with Breathe-Easy and at least five times smaller with Micropore (**Figure 5C**). Altogether, these data reveal that the extent of the VOC-mediated plant effects decreases with improved gas exchange and thus with reduced volatile concentrations in the headspace. Importantly, since positive plant responses were recorded in the absence of accumulating CO_2_ and any other volatile (Micropore sealing), even transient exposure to low concentrations of the *Serendipita* VOCs appears to be sufficient to modulate plant development.

Thirdly, PDA-grown isolate 30, *S. indica* and *S. williamsii* were co-cultivated with two albino *Arabidopsis* mutants (*apg2* and *cla1*) and one pale-green mutant (*apg3*) in split-plate assays sealed with Breathe-Easy (**Figures 5D, E**; **Supplementary Figure S12C**). The mutations in all three lines interfere with the early development of chloroplasts, leading to defective internal membrane structures and hampered photosynthesis (**Supplementary Table S1**). Consequently, they are insensitive to elevated CO_2_ levels and can be considered as biosensors to study the effects of volatile compounds other than CO_2_. To compensate for their photosynthesis defect, mutant seedlings were grown on ½ MS medium with 1% sucrose, but plant growth was still very poor. No significant VOC-mediated effects were recorded for *apg3* and *cla1*. However, after 14 days of co-cultivation, shoot growth in the *apg2* mutant was stimulated under the influence of VOCs from isolate 30 and *S. indica* (**Figures 5D, E**; **Supplementary Figure S12C**). These data support our previous finding that the tested *Serendipita* isolates do produce PGP volatiles besides CO_2_.

Then, we assessed whether CO_2_ concentrations produced by different fungi, irrespective of their lifestyle, positively correlated with the observed growth responses. Therefore, we evaluated the impact of the VOC blends emitted by the phytopathogen *Cochliobolus sativus* and the saprophyte *Aspergillus fumigatus* on *Arabidopsis* in Petri-dish-in-box assays (**Supplementary Figure S13A**) and quantified the CO_2_ levels generated by these fungi. Plants responded to the VOCs of *C. sativus* with a 1.9-fold enhancement of shoot FW, indicating that they may not be able to discriminate pathogenic from non-pathogenic fungi upon perception of volatiles (Moisan et al., 2019). This result also shows that fungal VOC-driven growth promotion, speculated to be a preparatory step towards hosting the microorganism, is controlled by conserved regulatory mechanisms, which in case of phytopathogenic strains could ensure proper continuation into the pathogenic phase (Sánchez-López et al., 2016a). Exposure to *A. fumigatus* volatiles resulted in a 4.5-fold higher FW, comparable to the PGP activity of *Serendipita* (**Supplementary Figure S13B**). Accordingly, whereas the F_v_/F_m_ ratio and the anthocyanin levels triggered by *A. fumigatus* were similar to those of the *Serendipita* isolates, the increases caused by the *C. sativus* VOCs were smaller (**Supplementary Figures S13C, D**). Furthermore, the VOCs released by the pathogen reduced the ChlIdx by 32.5%, whereas the saprophyte provoked a chlorophyll response similar to that of the *Serendipita* cultures (**Supplementary Figure S13E**). Interestingly, in contrast to its rather weak VOC-based PGP abilities, *C. sativus* generated 8500 ppm CO_2_ when grown on PDA, which is close to the levels recorded for the *Serendipita* isolates. On the other hand, as opposed to its strong VOC-induced plant growth effects, *A. fumigatus* produced only 4000 ppm CO_2_. Thus, although it cannot be excluded that the pathogen releases VOCs with an adverse impact on plant development, the comparable increase in plant biomass obtained with *Serendipita* and *Aspergillus* suggests that the PGP effects cannot solely be attributed to respiratory CO_2_.

**Figure 6:**
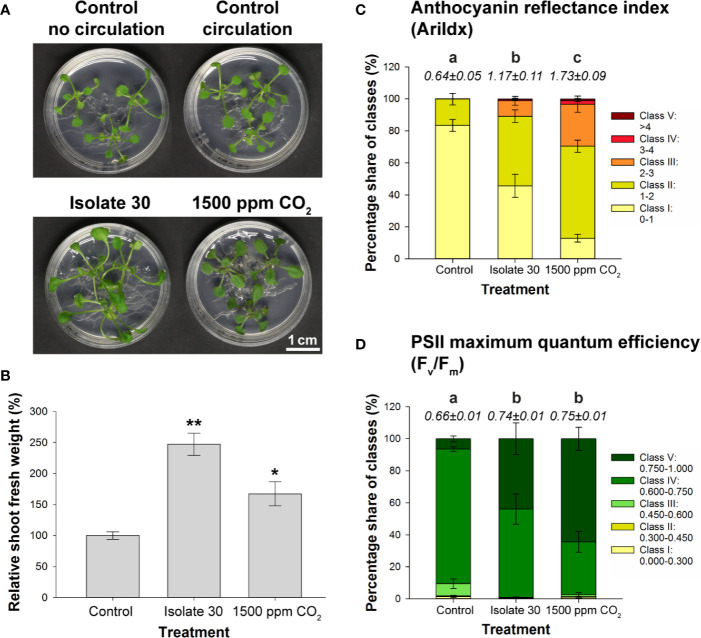
Comparison of the effects of exposure to 1500 ppm CO_2_ and *Serendipita* VOCs on *Arabidopsis* in 4‐L glass reaction vessels. **(A)** Growth responses in shoots and roots of plants grown on ½ MS without sucrose after a 7-day treatment with either volatiles from PDA-grown isolate 30 or 1500 ppm CO_2_, as compared to the two control treatments. **(B)** Average shoot fresh weight of *Arabidopsis* plants subjected to the above treatments, expressed relative to the control. Asterisks indicate statistically significant differences with the control according to one-sided t-tests (* < 0.05, ** < 0.01). **(C, D)** Distribution of pixels across different classes of the spectral parameter anthocyanin (AriIdx, **C**) and of the chlorophyll fluorescence parameter F_v_/F_m_ **(D)**. Average total values of the two parameters (± standard errors) for each of the evaluated treatments are shown above the respective stacked bars. Treatments sharing the same letter do not exhibit statistically significant differences according to Tukey HSD *post hoc* tests (α = 0.05). For a visualization of the pixel distribution across the different classes in the shoots, see **Supplementary Figure S14B**. Graphical data for chlorophyll content (ChlIdx) are presented in **Supplementary Figure S14C**. Error bars in all displayed graphs indicate standard errors on the mean of three replicates with each replicate representing three plants (each vessel contained three Petri plates, which were considered as internal biological replicates). When we refer to “Control,” the data from the control vessel with air circulation are shown (does not differ from the control without circulation; see **A**).

Finally, a CO_2_‐fertilization experiment was performed to determine which responses can be attributed to elevated CO_2_ levels. By adding a 99.85/0.15% N_2_/CO_2_ gas mixture to a 4-L glass vessel containing *Arabidopsis* seedlings, a physiologically relevant concentration of 1500 ppm CO_2_ was created (**Supplementary Figure S2**). As indicated by the fluctuating CO_2_ levels, the plants quickly consumed the provided CO_2_ (**Supplementary Figure S14A**). Therefore, the target level of 1500 ppm had to be restored once a day (first 3 days) up to two/three times a day (last 4 days). Controls consisted of a closed circuit without external CO_2_ supply and static setups without or with VOC treatment (PDA-grown isolate 30). Also in this setup, exposure to the *Serendipita* VOCs resulted in larger shoots with longer petioles and bigger, curved leaves, and an extension of the root system (**Figure 6A**). CO_2_ fertilization, on the other hand, influenced plant growth in a different way: the shoots were not bigger but appeared more robust and much darker; also the roots were thicker and more robust, but the root system did not expand (**Figure 6A**). Although both treatments affected shoot FW compared to the untreated controls, fungal VOC-stimulated plant biomass was 48% higher than that obtained with CO_2_ fertilization (**Figure 6B**). Anthocyanin levels were three times higher in shoots of CO_2_-exposed plants compared to control seedlings, whereas a twofold increase was measured in shoots subjected to fungal VOCs (**Figure 6C**; **Supplementary Figure S14B**), explaining the leaf color difference between these plants. Furthermore, while the chlorophyll content of the shoots was not altered by CO_2_ fertilization or VOC exposure (**Supplementary Figures S14B, C**), both treatments significantly and comparably increased the PSII maximum quantum efficiency (**Figure 6D**; **Supplementary Figure S14B**).

Altogether, these data indicate that the plethora of plant responses obtained by *Serendipita* volatile exposure are the combined result of CO_2_ fertilization originating from fungal respiration and other volatiles that trigger particular morphological modifications.

### Methyl Benzoate as One of the Most Abundant Compounds in *Serendipita* Volatilomes Does Not Affect Plant Development

**Figure 7:**
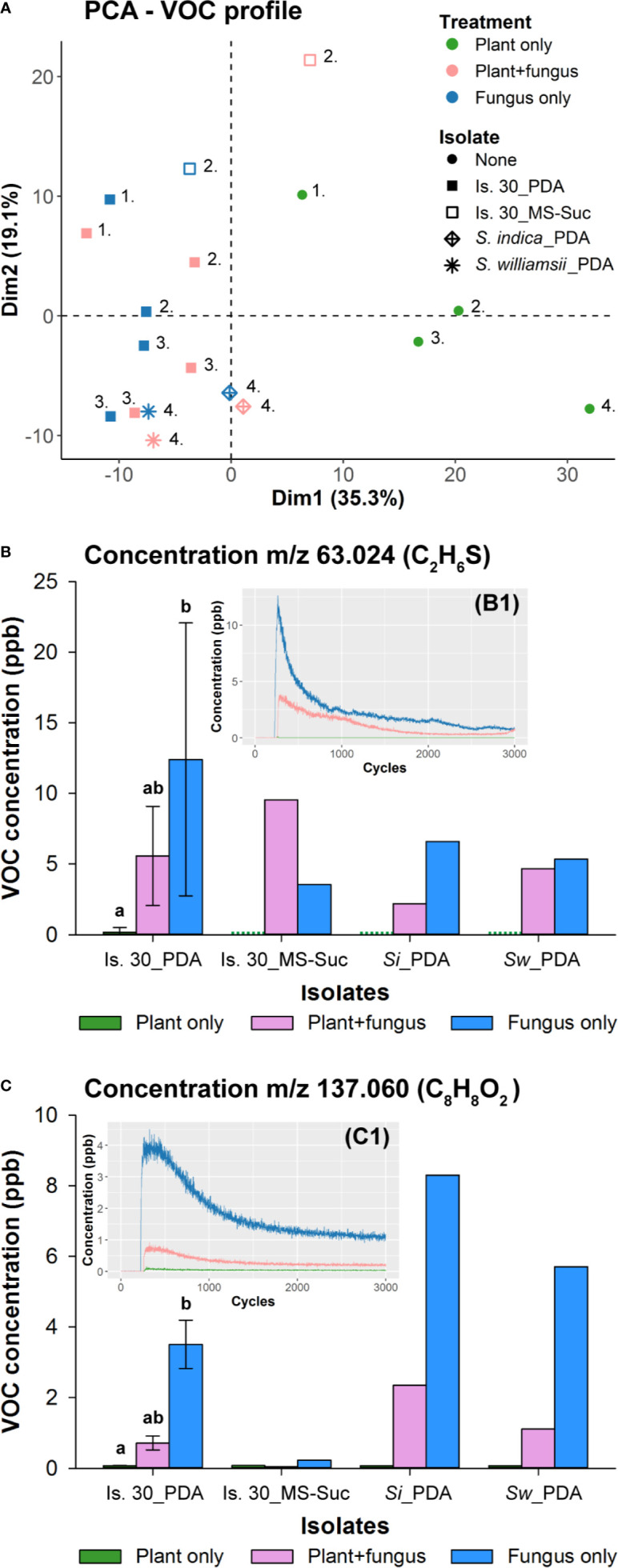
Determination of *Serendipita* volatile profiles by direct headspace analysis using PTR-TOF-MS. **(A)** PCA plot visualizing general differences in the samples’ volatile profile as measured after 4 days of (co-)cultivation *via* PTR-TOF-MS. Color codes indicate whether the vessel contained either *Arabidopsis* cultures (green; grown on ½ MS without sucrose), fungal cultures (blue; isolate 30 cultured on either PDA or ½ MS without sucrose, *S. indica* on PDA, or *S. williamsii* on PDA) or both of them adjacent to each other (pink). Symbols refer to the different fungal strains and medium conditions tested. Numbers signify which samples were analyzed on the same day. The position of the samples in the reduced ordination space reveals that neither the two fungal conditions evaluated per isolate (co-cultivated with *Arabidopsis* or not) nor the three *Serendipita* isolates studied here can be distinguished based on their volatile profiles. The only exception to this general observation is isolate 30 cultured on ½ MS without sucrose: the VOC composition of this culture is different from that of the PDA cultures and appears to be modified in the presence of plants. **(B, C)** Concentrations of the two compounds that were most abundant in the headspace of the *Serendipita* cultures. These VOCs were detected at m/z ratios 63.024 **(B)** and 137.060 **(C)** during PTR-TOF-MS analysis, corresponding to DMS (C_2_H_6_S) and methyl benzoate (C_8_H_8_O_2_), respectively. Color codes are the same as in **(A)**. The green dots for the plant controls in **(B)** stand for a concentration of 0 ppb. Experiments with isolate 30 on PDA were set up on three different days. Error bars represent standard deviations; treatments sharing the same letter above their respective bars are not significantly different according to a Dunn’s test adjusted for multiple comparisons (Bonferroni correction; α = 0.05). *S. indica* and *S. williamsii* (and isolate 30 on ½ MS) were evaluated in a single experiment (no error bars or statistics). The inset graphs (B1 and C1) show that the recorded concentration decreases over the time span of the sample measurement (2700 cycles or 45 min), resulting in a clear decay pattern. For each individual analysis, only the signals from 60 consecutive cycles at the beginning of the measurement were used to calculate an average concentration per compound. Is., Isolate; MS, ½ Murashige and Skoog medium; PDA, potato dextrose agar; *Si*, *Serendipita indica*; Suc, Sucrose; *Sw*, *Serendipita williamsii*.

To get insight into the nature of these volatile compounds, we analyzed the headspace composition of PDA-grown isolate 30, *S. indica* and *S. williamsii*, which were placed without or with plant cultures inside 4-L glass vessels, as well as that of isolate 30 cultured on ½ MS without sucrose. After 4 days of (co‐)cultivation, the vessels were flushed with N_2_ gas and the compounds in the VOC-enriched outlet flow were directly measured by PTR-TOF-MS, while the VOCs from a diverted side flux were pre-concentrated on Tenax TA/Carbotrap sorbent tubes for TD-GC-MS analysis. After evaluation of the PTR-TOF-MS results, a comprehensive data set of 399 VOCs was retained across all conditions and isolates. PCA analysis revealed that the VOC profiles of the plant controls and the fungal treatments were separated from each other along the plot’s first axis, which explained 35.3% of the original variance in the data set (**Figure 7A**), but both conditions evaluated per isolate (co-cultivated with *Arabidopsis* or not) or the three *Serendipita* isolates could not be distinguished based on their volatile profiles.

To facilitate the selection of VOCs linked to *Serendipita* growth, both the orientation and importance of the individual compounds (PCA variables) were assessed using their loadings (correlations with the principal components plotted as X/Y coordinates) and their cos2 values (squared loadings summed for both principal components), respectively. Of all 399 variables, only those with a cos2 value higher than 0.25 were retained, resulting in a reduced data set of 330 variables. Subsequently, compounds were considered to be associated with the fungal treatments if their loading on the first dimension (X‐coordinate) was below −0.20, leaving 30 compounds for further processing. As a last variable-reducing step, only those compounds displaying a decay in concentration over the time span of the sample measurement (2700 cycles or 45 min; e.g., **Figures 7B1, C1**) were subjected to identification. As such, the final subset of 12 fungal VOCs (indicated by their m/z ratio and tentative chemical formula) consisted of: m/z 62.026, m/z 63.024 ((C_2_H_6_S)H^+^), m/z 64.026 (^13^C isotope of (C_2_H_6_S)H^+^), m/z 75.042 ((C_3_H_6_O_2_)H^+^), m/z 89.058 ((C_4_H_8_O_2_)H^+^), m/z 90.062 (^13^C isotope of (C_4_H_8_O_2_)H^+^), m/z 93.035 ((C_6_H_4_O)H^+^), m/z 94.038 (^13^C isotope (C_6_H_4_O)H^+^), m/z 105.031 (C_7_H_5_O^+^, fragment ion of C_8_H_8_O_2_)H^+^), m/z 110.982, m/z 137.060 ((C_8_H_8_O_2_)H^+^), m/z 138.062 (^13^C isotope of C_8_H_8_O_2_)H^+^). The most abundant compounds, based on non-fragmented main (^12^C) isotope concentrations, were detected at m/z 63.024 ((C_2_H_6_S)H^+^) and m/z 137.060 ((C_8_H_8_O_2_)H^+^) (**Figures 7B, C**; **Supplementary Figure S15A**). The concentrations produced by the PDA cultures varied from 5.34 ppb (*S. williamsii*) to 26.59 ppb (isolate 30) for m/z 63.024, and from 2.53 ppb (isolate 30) to 8.30 ppb (*S. indica*) for m/z 137.060; the values of the plant controls did not exceed 0.70 ppb or 0.08 ppb, respectively, and those of the co-cultivation condition were situated in between (**Figures 7B, C**). Furthermore, in the headspace of isolate 30 grown on sucrose-free ½ MS, the level of m/z 137.060 was negligible, whereas for m/z 63.024 it was similar to that of the PDA cultures. When expressing volatile production relative to the dry weight of the fungal mycelium, the level of m/z 137.060 was comparable for both media, whereas in case of m/z 63.024 the released amounts were higher on ½ MS than on PDA (**Supplementary Figure S15B**).

As a final identification step, the VOCs collected on the sorbent tubes were analyzed by TD-GC-MS. Two compounds, identified as dimethyl sulfide (DMS; C_2_H_6_S) and methyl benzoate (C_8_H_8_O_2_) based on their MS spectra and the NIST mass spectral library, were more strongly represented in the fungal treatments than in the plant controls. These two VOCs corresponded to the compounds with respective m/z ratios of 63.024 and 137.060 detected by PTR-TOF-MS. The identity of these compounds was further confirmed by their retention time (and mass spectrum), which was identical to that of reference standards of DMS and methyl benzoate (**Supplementary Figure S16**).

Given that methyl benzoate (C_8_H_8_O_2_) was abundantly released by PDA cultures, which triggered strong plant responses, we investigated the plant response to this compound only, using two setups. First, three plates with *Arabidopsis* growing on ½ MS without or with 1% or 3% sucrose were placed adjacent to a water plate spiked with two different concentrations of methyl benzoate inside 500-ml polypropylene boxes and Duran glass bottles (**Supplementary Figure S1E**). For the lowest dose in the Duran system, the methyl benzoate level was monitored twice a day over the time course of the experiment in a separate bottle without plants connected to the PTR-QiTOF instrument. A fluctuating concentration between 25 and 50 ppb was recorded during the first 3 days, after which the methyl benzoate level decreased steadily to reach ca. 7 ppb at day 7 (data not shown), which is in the range of the *Serendipita*-produced level (**Figure 7C**). Under none of the conditions tested, plant responses or changes in plant morphology reminiscent of co‐cultivation with *Serendipita* were observed (**Supplementary Figures S17A, B**). Secondly, seedlings grown on ½ MS without and with 3% sucrose in a 4-L glass vessel were subjected to a continuous flow of methyl-benzoate-enriched air *via* an in-house developed gas-generation system, which ensured a stable concentration of ca. 20 ppb over time (**Supplementary Figure S3**). After 7 days, the phenotype of the treated plants was not different from the controls (data not shown). Nevertheless, for the plants grown on sucrose-free medium, the shoot biomass did increase under the influence of methyl benzoate (**Supplementary Figure S17C**).

Altogether, these data indicate that methyl benzoate applied as an individual molecule in our experimental systems cannot mimic the effects induced by *Serendipita* VOC mixtures, implying that this volatile is not implicated in the plant responses or that a combination of several volatile compounds is needed to affect plant performance and development.

### Screening of *Arabidopsis* Mutants and Reporter Lines Suggests the Involvement of Auxin and Cytokinin Signaling in *Serendipita* VOC-Mediated Plant Growth Promotion

**Figure 8:**
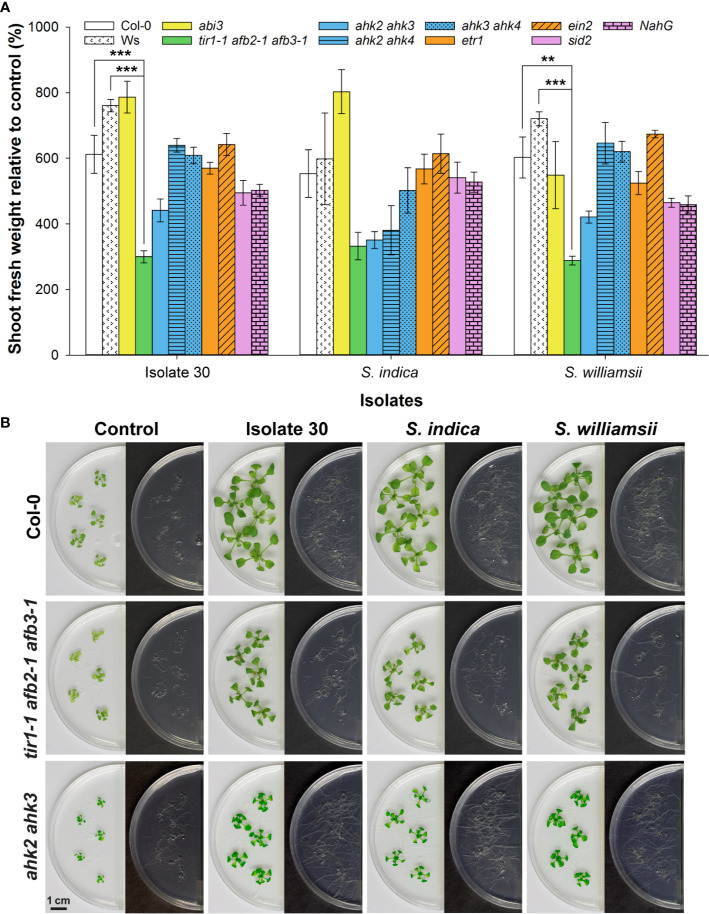
Split-plate assay with *Arabidopsis* hormone-related mutants to explore the mechanisms underlying *Serendipita* VOC-mediated growth promotion. **(A) **Average shoot fresh weight of *Arabidopsis* wild‐type and mutant plants grown on ½ MS without sucrose after being exposed for 10 days to volatiles of isolate 30, *S. indica* and *S. williamsii* cultured on PDA, expressed relative to the corresponding untreated control. The bar color indicates the hormone type studied: white = wild type (Col-0 and Ws), yellow = abscisic acid (*abi3*), green = auxin (*tir1-1 afb2-1 afb3-1*), blue = cytokinin (*ahk2 ahk3*, *ahk2 ahk4* and *ahk3 ahk4*), orange = ethylene (*etr1* and *ein2*), purple = salicylic acid (*sid2* and *NahG*). All lines are derived from the Col-0 ecotype, except for *tir1-1 afb2-1 afb3-1*, which has a mixed Col-0 (*tir1-1*)/Ws (*afb2-1 afb3-1*) background. Error bars represent standard errors on the mean of three biological replicates with each replicate consisting of five plants. For each of the three isolates tested, the magnitude of the growth response in the mutant lines was compared with that in the corresponding wild type by performing a Dunnett’s test; asterisks indicate statistically significant differences (** < 0.01, *** < 0.001). **(B)** Shoot and root growth in the Col-0 wild type and the two least responsive mutant lines after 10 days of co-cultivation with isolate 30, *S. indica* and *S. williamsii*. A complete overview showing the responses in all mutants is given in **Supplementary Figure S18**. Plates were sealed with Breathe-Easy strips.

To gain insight into the mechanisms by which *Serendipita* volatiles enhance plant growth, *A. thaliana* mutants defective in specific hormone signaling or biosynthesis pathways were exposed to VOCs from isolate 30, *S. indica* and *S. williamsii*, and the magnitude of their response was compared to that of wild-type plants (**Figures 8A, B**; **Supplementary Figure S18**). The ability to respond to *Serendipita* VOCs was not impaired in the *abi3* mutant, which has a defective transcription factor essential in mediating abscisic acid (ABA) action (Giraudat et al., 1992). Similarly, mutations in either the ethylene receptor ETR1 or the EIN2 protein that participates in the downstream signal transduction, both causing ethylene insensitivity (Alonso and Stepanova, 2004), did not result in a reduced responsiveness. Also for the salicylic acid (SA)-induction-deficient mutant *sid2*, which is unable to accumulate SA due to a defect in isochorismate synthase 1 (Wildermuth et al., 2001), and the transgenic line *NahG*, in which de SA-degrading enzyme salicylate hydroxylase is expressed (Friedrich et al., 1995), biomass stimulation was not compromised. These data suggest that ABA, ethylene, and SA signaling pathways are not involved in mediating growth enhancement upon *Serendipita* VOC exposure.

In contrast, the magnitude of plant growth promotion was approximately halved in the auxin perception triple mutant *tir1-1 afb2-1 afb3‐1* (**Figures 8A, B**) that has a reduced auxin-regulated gene expression because of mutations in the auxin receptors TIR1, AFB2, and AFB3 (Dharmasiri et al., 2005), indicating that the *Serendipita* volatiles depend on at least one of these three receptors to stimulate shoot and root development. Furthermore, especially when co-cultivated with *S. indica*, a reduced (statistically non-significant) shoot growth enhancement was recorded for the *ahk2 ahk3* mutant (**Figures 8A, B**). This observation implies that the VOC-evoked plant effects might also be regulated by cytokinins, with presumably a role for AHK2 and AHK3, which are both primarily expressed in the aerial part of the plant (Higuchi et al., 2004). Although no root quantifications are possible within this experimental system, mutations in the cytokinin receptors did not lead to an apparent impairment of VOC-induced root system extension (**Figures 8A, B**).

**Figure 9:**
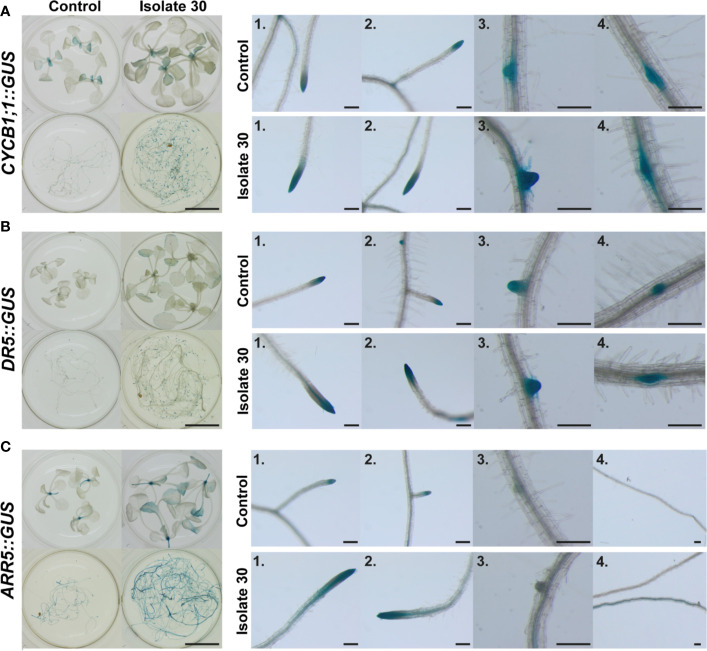
*CYCB1;1::GUS* **(A)**, *DR5::GUS* **(B)** and *ARR5::GUS* **(C)** expression in the shoots and roots of control and VOC-exposed *Arabidopsis* seedlings after 7 days of co-cultivation with *Serendipita* in split plates. Results are shown for *Serendipita* isolate 30; identical responses were recorded with *S. indica* and *S. williamsii*. The panels on the right display details on the GUS activity in lateral root tips (1 and 2) and in newly emerging lateral roots (3) or newly established lateral root primordia (4). Compared to the corresponding controls, GUS expression in the VOC-treated shoots was stronger and expanded only for *ARR5::GUS*, showing activity in the rosette leaves, aside from expression in the shoot apical meristem. With regard to the VOC-exposed root system, a higher and more extensive staining as compared to the control was observed in the root tips of all three reporter lines (see insets 1 and 2) and in the developing lateral roots/primordia of the *CYCB1;1::GUS* and *DR5::GUS* seedlings (see insets 3 and 4 in **A, B**). *ARR5::GUS* expression was weak or absent in lateral root initiation sites in treated (and non-treated) plants (see inset 3 in C), but large areas of the pericycle were activated in several parts of the root system upon VOC exposure (see inset 4 in **C**). Scale bars = 5 mm for the overview pictures on the left and 200 µm for the zooms on the right.

To corroborate the finding that mainly auxin and cytokinin signaling are involved in mediating *Serendipita* VOC-induced plant growth promotion, we evaluated the GUS expression patterns in marker lines for cell division activity (*CYCB1;1::GUS*), auxin responsiveness (*DR5::GUS*), and cytokinin signal transduction (*ARR5::GUS*) in split-plate assays with isolate 30, *S. indica*, and *S. williamsii*. Despite the fact that clear macroscopic signs of enhanced shoot growth were already visible after 2 days of co-cultivation, histochemical staining of 7-day-old seedlings revealed that *CYCB1;1::GU*S nor *DR5::GUS* expression was upregulated in the VOC-treated shoots (**Figures 9A, B**). Under both conditions, *CYCB1;1* was only expressed in the apical meristem and in young developing leaves (**Figure 9A**), supporting our previous finding that leaf area expansion during co-cultivation is attributed to cell size enlargement and not to accelerated cell proliferation. *DR5::GUS* activity in control and treated shoots was restricted to the hydathodes, although occasionally the expression zone was expanded along the rim of VOC-exposed leaves (**Figure 9B**). Expression of the A-type *Arabidopsis* response regulator gene *ARR5*, a cytokinin primary-response gene, was restricted to the apical meristem in control shoots, which is consistent with cytokinins having a role in the regulation of cell division (Hare and van Staden, 1997), whereas in case of the *Serendipita* VOC treatments, it was also detected in several rosette leaves, suggesting a moderate increase in cytokinin signaling under these conditions (**Figure 9C**).

In the root system, however, the staining patterns in the three reporter lines revealed that lateral root formation, i.e., the local activation of pericycle cells and the establishment of new meristems along the primary root (Laskowski et al., 1995), was strongly induced by exposure to *Serendipita* VOCs compared to control plants: a more intense and extensive GUS activity was detected in the lateral root tips and emerging lateral root primordia in the *CYCB1;1::GUS* and *DR5::GUS* plants (**Figures 9A, B**), whereas *ARR5::GUS* expression was stronger in the entire root system and particularly occurred in a larger area of the root tip (**Figure 9C**). Daily analysis of *CYCB1;1::GUS* seedlings co-cultivated with isolate 30 in split plates for 4 days illustrated the kinetics of this VOC-induced lateral root initiation. From day 2 onwards, a stronger *CYCB1;1::GUS* expression was detected in the apical meristems of the primary and the lateral roots and at newly established meristems in the pericycle (**Supplementary Figure S19**). Whereas there was no difference in lateral root and primordium density between control and exposed plants during the first 2 days of co-cultivation, at day 3 numerous lateral root initiation sites emerged in the apical part of the primary root. As such, under the influence of *Serendipita* VOCs, the combined density of initiation sites and established lateral roots had increased 1.5 times at day 3 and was doubled by day 4.

**Figure 10:**
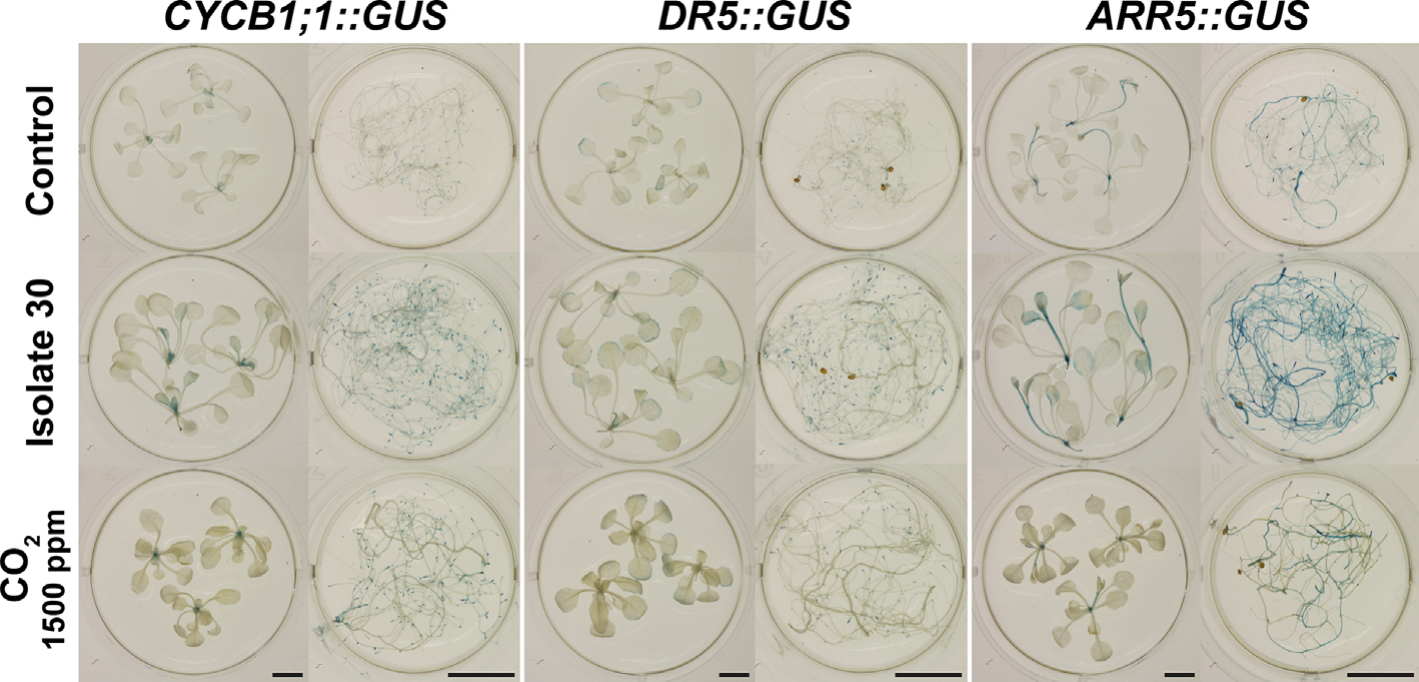
Effects of exposure to *Serendipita* VOCs compared to those caused by CO_2_ treatment (1500 ppm; see **Figure 6**) with respect to GUS activity. GUS expression patterns in the shoots and roots of the reporter lines *CYCB1;1::GUS*, *DR5::GUS* and *ARR5::GUS*, evaluated after 7 days of VOC/CO_2_ “fertilization,” are displayed. When we refer to “Control” without further specification, the images from the control vessel with air circulation are shown. Scale bars = 5 mm.

Finally, the expression patterns of the three reporter lines were also evaluated in a CO_2_ fertilization experiment. Overall, in the shoots of *CYCB1;1::GUS* and *DR5::GUS* reporter lines, CO_2_ treatment nor VOC exposure modified the GUS expression patterns observed in the controls, i.e., it was mainly located in the shoot apex and young leaves for *CYCB1;1::GUS* and in the hydathodes for *DR5::GUS* (**Figure 10**). In CO_2_-fertilized shoots, *ARR5::GUS* expression was also comparable to that in the controls, which is in contrast to the higher staining intensity recorded in VOC-exposed plants (**Figure 10**). Importantly, in the roots of CO_2_-exposed plants, only *CYCB1;1::GUS* staining in the root tips was stronger compared to the non-treated root apical meristems, while *DR5::GUS* and *ARR5::GUS* expression was similar to that in the control plants (**Figure 10**). These observations are in line with the lack of expansion of the root system upon CO_2_ fertilization and in contrast to the enlarged root system and concomitant enhanced GUS activity in the three reporter lines subjected to *Serendipita* VOCs (**Figure 10**). In conclusion, the differential responses between VOC and CO_2_-treated seedlings mainly signify an increased cytokinin signaling upon VOC exposure that is not caused by fungal respiratory CO_2_.

## Discussion

*Serendipita indica* has been praised for its positive effects on a wide variety of plants, including enhanced growth, biomass and yield of agricultural, horticultural and medicinal plants (Varma et al., 1999; Barazani et al., 2005; Achatz et al., 2010), increased resistance/tolerance to (a)biotic stresses (Waller et al., 2005; Sherameti et al., 2008; Rabiey et al., 2015; Zhang et al., 2018), stimulated adventitious root formation in cuttings (Druege et al., 2007), and improved hardening and acclimatization of micropropagated plantlets (Das et al., 2017). Plants in soil- and agar-based experimental systems have been reported to react to *Serendipita* inoculation already prior to the establishment of a symbiotic interaction. Although non-volatile diffusible compounds were thought to be the major chemical mediators of this early response (Vahabi et al., 2015), we demonstrated here that the Congolese *Serendipita* isolates as well as the reference strains *S. indica* and *S. williamsii* produce volatile compounds that strongly promote plant growth. Indeed, despite quantitative differences, across four experimental setups, compared to untreated controls, *Arabidopsis* plants growing on sucrose-free ½ MS exposed to VOCs of PDA-grown *Serendipita* cultures exhibited an up to 9.3 times higher shoot FW (**Figure S10**). Vertically grown plants subjected to fungal VOCs revealed significant increases in primary and lateral root length and lateral root density, contributing to the overall expansion of the root system. Additionally, plants treated with *Serendipita* VOCs manifested morphological and physiological changes, including an overall more robust appearance, darker leaves, elongated petioles, a larger leaf surface area, a higher level of anthocyanin and probably of starch, and an improved maximum quantum efficiency of PSII, but a rise in chlorophyll content was not detected. Except for the latter, these *Serendipita* VOC-mediated plant growth effects largely correspond to those recorded for other microbial volatiles (reviewed in e.g., Kanchiswamy et al., 2015).

Besides these plant responses, another commonality across studies on microbial VOCs is that depending on the experimental setup (Kai and Piechulla, 2009; Kai and Piechulla, 2010; Kai et al., 2016) and the growth conditions of the microbe (Blom et al., 2011; Asari et al., 2016; González-Pérez et al., 2018; Rath et al., 2018), the effect on the exposed plants can range from strongly positive to neutral and even deleterious. Also in this study, the extent of the elicited modifications depended on the experimental system (**Figure S10**) and conditions. For instance, significantly less pronounced or even no effects were obtained when gas exchange was improved by sealing the plates with Breathe-Easy or Micropore instead of plastic foil, indicating that the volatile concentration in the headspace is important. Additionally, the *Arabidopsis* nutrient source was a determining factor for the extent of plant growth promotion. More specifically, the magnitude of the PGP effects in response to *Serendipita* VOCs decreased with increasing levels of sucrose in the plant medium, but the exposed plants always outperformed control plants. Furthermore, also in the *Serendipita*-*Arabidopsis* system, the fungal growth medium and hence changes in fungal metabolic activity significantly impacted the outcome of VOC exposure. The most pronounced shoot and root responses were induced when *Serendipita* isolates were cultured on rich fungal media, whereas growth on poorer media provoked intermediate or no alterations in plant development. In support of these findings, the volatile profiling indicated that the growth conditions of *Serendipita* affected the VOC composition and that volatilomes were comparable amongst isolates grown under the same conditions. Even more, these data provide a tentative explanation for the conclusion of Vahabi et al. (2015) that diffusible compounds rather than VOCs from *S. indica* were responsible for the modulation of gene expression that was detected in *Arabidopsis* roots before the two partners were in physical contact. Indeed, because they grew the fungus on nutrient-poor medium, it would not have produced plant-modifying VOCs. Similarly, in our previous direct-contact experiments (Venneman et al., 2017), the observed shoot and root alterations occurring before the physical connection between *Arabidopsis* and *Serendipita* could not have been caused by fungal volatiles as both organisms were co-cultivated on sucrose-free ½ MS medium. Importantly, these findings signify that in natural settings both volatile and non-volatile compounds released by *Serendipita* species likely play a role as chemical mediators of early communication in sebacinoid symbioses.

A recurring enigma in studies examining plant responses under microbial VOC exposure is the relative importance of CO_2_ versus other volatiles emitted by the microorganisms. Whereas some studies provided strong evidence for the contribution of respiratory CO_2_ to the microbial VOC-elicited plant growth effects (Kai and Piechulla, 2009; Casarrubia et al., 2016), other reports have implied the involvement of volatile compounds other than CO_2_, for instance in lateral root stimulation induced by *L. bicolor* volatiles (Ditengou et al., 2015) or increased shoot FW triggered by VOCs of *Escherichia coli* (Bailly et al., 2014), *F. oxysporum* (Bitas et al., 2015), and *Ampelomyces* and *Phoma* strains (Naznin et al., 2013). The positive correlation between the mycelial biomass, the plant responsiveness, and the level of generated CO_2_ provided strong evidence for a major role of CO_2_ in the *Serendipita*-mediated growth enhancement of *Arabidopsis*. However, the finding that the provoked plant growth promotion by *Serendipita* isolates was much stronger than that triggered by the pathogenic fungus *C. sativus*, despite the fact that they released similar amounts of CO_2_, and comparable to that recorded with *A. fumigatus*, which emitted less CO_2_, suggested that CO_2_ is not the only driver of the observed plant effects. A similar lack of correlation between produced CO_2_ levels and plant responses across microbial species has been noted before (Naznin et al., 2013; Bitas et al., 2015; Moisan et al., 2019). Additionally, capturing headspace CO_2_ in our co-cultivation assays using Ba(OH)_2_ reduced but did not annihilate the increase in *Arabidopsis* shoot FW. Similarly, improvement of gas exchange, which lowered the CO_2_ levels in the closed systems, still led to bigger, darker and more robust VOC-treated plants. Even more, the VOC-responsiveness of the *apg2* chloroplast mutant which is not photoautotrophic can only be ascribed to volatile compounds other than CO_2_. The fact that the other two chloroplast mutants (*apg3* and *cla1*) did not respond to the fungal VOCs might be due to their distinct metabolome and transcriptome profiles (Satou et al., 2014). Furthermore, CO_2_ fertilization resulted in darker and more robust shoots with a stronger accumulation of anthocyanin and a higher PSII maximum quantum efficiency, which is in agreement with other studies (Crookshanks et al., 1998; Makino and Mae, 1999; Poorter and Navas, 2003; Tallis et al., 2010; Jauregui et al., 2015; van der Kooi et al., 2016; Thompson et al., 2017). Although the kinetics of CO_2_ provision upon *Serendipita* VOC exposure and CO_2_ fertilization are probably different, the typical phenotypic alterations caused by the fungal volatiles did not occur under the CO_2_-enriched conditions. In this respect, also Zhou et al. (2017) did not obtain larger rosettes under elevated CO_2_ levels and García-Gómez and co-workers (2019) showed that exposure to 2000 ppm CO_2_ did not provoke a strong increase in shoot biomass or distinct changes in root architecture. Finally, unlike *Serendipita* VOC treatment, neither *DR5::GUS* activity in the root system nor *ARR5::GUS* expression in the roots and shoots was upregulated after CO_2_ fertilization. Based on the combined results of these different approaches, which individually each have their limitations, we conclude that the observed plant growth promotion in the *Serendipita*-*Arabidopsis* system is strongly supported by respiratory CO_2_ but diverse aspects of the morphological and physiological changes are attributed to other, currently unidentified volatile compounds.

In search of plant growth-stimulating *Serendipita* volatiles, the two most abundant differential volatile compounds linked to the fungal metabolism were identified as DMS and methyl benzoate. When the fungal biomass was not taken into account, considerable levels of DMS were recorded across all fungal treatments regardless of the medium used, whereas methyl benzoate concentrations were lower when *Serendipita* was cultured on sucrose‐free ½ MS medium as compared to PDA. Therefore, we hypothesized that methyl benzoate might be responsible for the PGP effects of the *Serendipita* VOCs. However, hardly any plant response was obtained in diverse bioassays evaluating the impact of methyl benzoate application as a sole volatile, except for a moderate increase in shoot FW in the absence of sucrose in the plant medium under a continuous methyl benzoate flow. Although it cannot be ruled out that methyl benzoate has no plant growth-modifying activity, it is possible that this compound only contributes to the observed growth enhancement when it is combined with other molecules of the volatile blend, which might have different, complementary modes of action. The fact that mixtures of volatiles, rather than single molecules, account for microbial VOC-mediated effects has been reported before (Strobel et al., 2001; Naznin et al., 2013; Garbeva and Weisskopf, 2020). Despite the absence of PGP activity in our experiments, biological functions have been attributed to methyl benzoate. It would have potential as a green insecticide (Feng and Zhang, 2017) or as a fumigant for the control of gray mold (*Botrytis cinerea*) during postharvest storage of fruits (Archbold et al., 1997). Additionally, methyl benzoate as part of the *Cladosporium*-produced volatilome plays a pivotal role in eliciting induced systemic resistance in *A. thaliana* against *Pseudomonas syringae* pv. *tomato* DC3000 (Naznin et al., 2014). Finally, in contrast to the neutral effect observed in our study, Horiuchi et al. (2007) showed that treatment of *Arabidopsis* seedlings with 1.0 µmol methyl benzoate caused root growth inhibition. Further experimentation would be required to validate the function of methyl benzoate, if any, in the *Serendipita*-*Arabidopsis* interaction.

To get insight into the mechanisms underlying the *Serendipita* VOC-mediated plant responses, we treated reporter lines and mutants in some of the major plant hormone signal transduction pathways with *Serendipita* VOCs. With the *CYCB1;1::GUS* reporter line, as a marker for actively dividing cells, strong VOC-induced cell division activity was detected in the roots, which is consistent with the expansion of the root system *via* activation of lateral root formation. In agreement with the observed increase in the size rather than in the number of leaf epidermal cells, no modulation of *CYCB1;1* expression was recorded in the shoots of VOC-treated plants. In line with this finding, transcriptome studies on the molecular basis of microbial VOC-induced morphological and physiological changes in plants revealed that the expression of genes encoding proteins with cell-wall loosening activity, including expansins, pectate lyases and pectinases, was upregulated, which at least partly explained the observed leaf area expansion (Zhang et al., 2007; Minerdi et al., 2011; Sánchez-López et al., 2016a; Lee et al., 2019). A modulation of auxin and cytokinin homeostasis was believed to be involved in the regulation of genes related to cell wall composition, rigidity and extensibility. Based on the reduced responsiveness of the triple auxin receptor-deficient mutant *tir1‐1 afb2‐1 afb3‐1* and the double cytokinin receptor knockout mutant *ahk2 ahk3*, together with the increased expression of *DR5::GUS* and *ARR5::GUS* upon *Serendipita* VOC exposure, we also identified auxin and cytokinin signaling as the main hormone pathways implicated in the growth-stimulating effects. In fact, these two phytohormones are emerging as the central regulators in the plant growth-modulating mechanisms of many microbial VOCs. Indeed, by using diverse approaches, including screening *Arabidopsis* mutants and/or reporter lines and interfering with hormone biosynthesis or transport (Zhang et al., 2007; Ortíz-Castro et al., 2008; Bhattacharyya et al., 2015; Bitas et al., 2015; Garnica-Vergara et al., 2016; Sánchez-López et al., 2016a; Pérez-Flores et al., 2017; Li et al., 2018) as well as transcriptomics (Zhang et al., 2007; Bhattacharyya et al., 2015; Sánchez-López et al., 2016a) and proteomics (Kwon et al., 2010; Sánchez-López et al., 2016b), it was shown that mainly auxin and cytokinin production and signaling as well as auxin transport were affected by microbial VOCs, indicating that plants react to these volatiles through highly conserved regulatory mechanisms. Interestingly, plant growth promotion due to physical contact between *S. indica* and *Arabidopsis* also appeared to be linked to auxin signaling (Schäfer et al., 2009; Lee et al., 2011; Dong et al., 2013; Ye et al., 2014; Hua et al., 2017) and cytokinin perception (through CRE1/AHK2; Vadassery et al., 2008).

Based on proteome (Kwon et al., 2010; Sánchez-López et al., 2016b; Ameztoy et al., 2019; García-Gómez et al., 2019) and transcriptome (Sánchez-López et al., 2016a) analyses, molecular responses associated with the improved photosynthetic efficiency upon VOC exposure mainly involve the differential regulation of proteins related to the photosystem machinery, carbohydrate metabolism and oxidative stress. In this respect, the high number of starch granules and elevated anthocyanin levels observed in the presence of *Serendipita* VOCs are most likely linked to the necessity of storing excess glucose due to enhanced photosynthetic CO_2_ fixation and the need to protect the cells against photo-oxidative damage caused by a higher production of reactive oxygen species due to an increased photosynthetic electron transport rate (Wang et al., 1997; Neill and Gould, 2003), respectively. Interestingly, expression of anthocyanin biosynthesis genes is stimulated by the synergistic action of sucrose and cytokinins (Das et al., 2012; Sánchez-López et al., 2016a). As an indication of the impact of *Serendipita* volatiles on the extent of sucrose/cytokinin-induced anthocyanin accumulation, the AriIdx values measured in plants growing on ½ MS medium without sucrose and subjected to VOCs released by PDA cultures were the equivalent of those obtained in control plants growing on ½ MS medium with 3% sucrose. This result can be ascribed to a combination of augmented sucrose levels (resulting from the enhanced photosynthesis) and increased cytokinin signaling under influence of the *Serendipita* volatiles. Lastly, the direct positive impact of *Serendipita* respiratory CO_2_ on net photosynthetic rate *via* maximization of the carboxylation rates of ribulose-1,5-biphosphate (RuBP) by RuBP carboxylase/oxygenase (Rubisco) (Makino and Mae, 1999; Ainsworth and Rogers, 2007) and augmentation of phytohormone concentrations and crosstalk (Yong et al., 2000; Li et al., 2002; Teng et al., 2006; Thompson et al., 2017) should also be considered.

**Figure 11:**
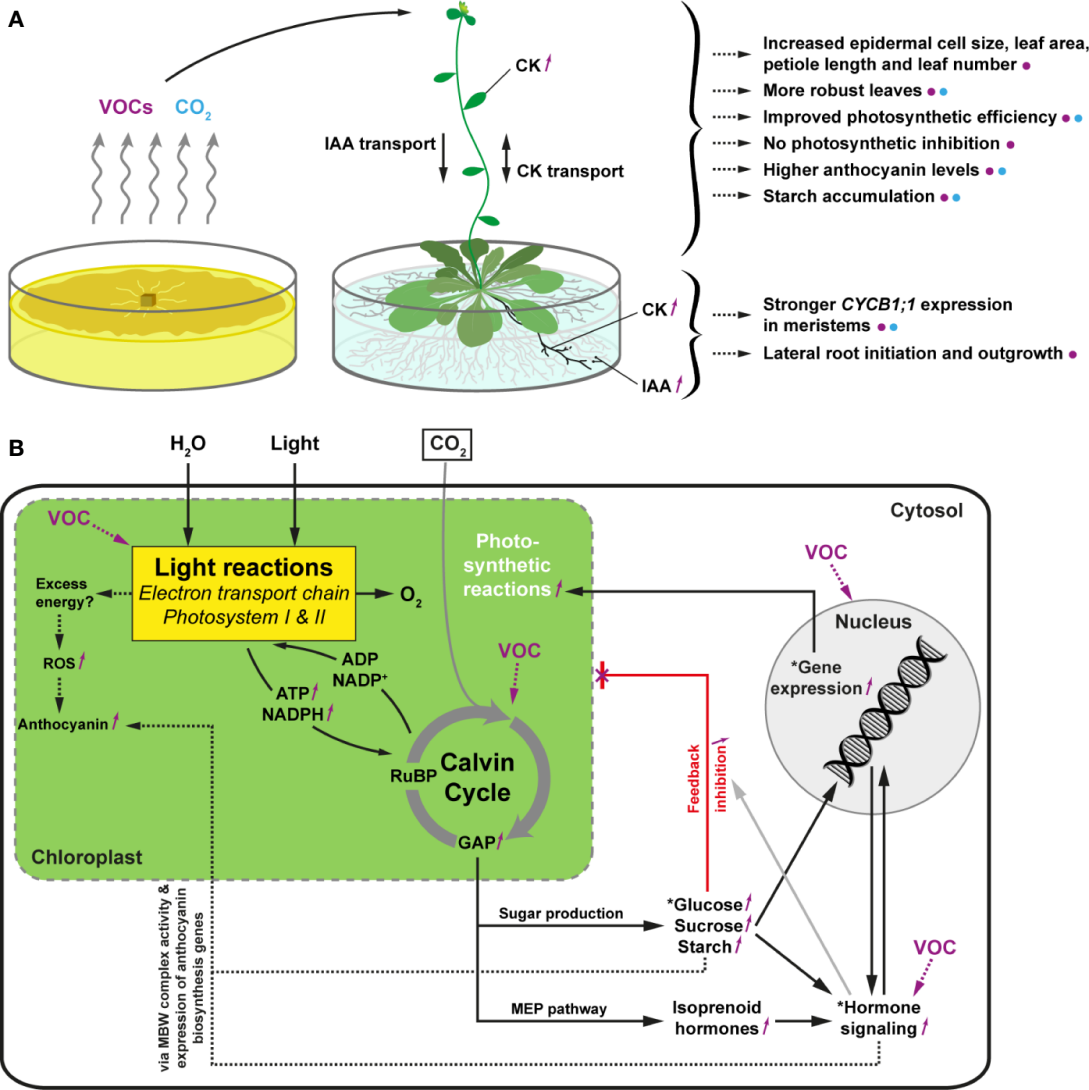
Overview of the responses observed in *Arabidopsis* seedlings exposed to *Serendipita* VOCs or 1500 ppm CO_2_ **(A)** and a tentative model explaining these observations **(B)**. **(A)** Morphological and physiological changes in roots (supposedly the primary target of rhizofungal VOCs) and shoots upon VOC (purple color) and CO_2_ (blue) treatment. The illustration of the *Arabidopsis* plant is modified from figshare.com. **(B)** Model summarizing the biological processes that might be affected in VOC-exposed plants, based on our own observations combined with transcriptome/proteome data from other studies (especially García-Gómez et al., 2019). *Serendipita* VOCs stimulate the photosynthetic reactions (both the light-dependent reactions (e.g., effect on photosystem proteins) and the Calvin Cycle), interfere with hormone signaling pathways, and induce gene expression (see purple markings). The resulting elevated sugar levels, altered hormonal crosstalk, and modified proteome (see asterisks) contribute to plant growth modulation in a direct or indirect way. Augmentation of photosynthetic activity induced by *Serendipita* VOCs and respiratory CO_2_ is associated with a rise in the production of the Calvin cycle intermediate glyceraldehyde 3‐phosphate (GAP). The increase in GAP leads to higher sugar levels and stimulates the accumulation of plastidial MEP pathway-derived isoprenoid compounds, including hormones (e.g., CK, ABA), resulting in changes in the expression of genes involved in many different processes. The impact of elevated CO_2_ is especially linked to an enhanced photosynthetic CO_2_ fixation through maximization of the carboxylation rates of RuBP by Rubisco. At the same time, the non-productive oxygenation side-reaction in which Rubisco uses O_2_ as a substrate to oxygenate RuBP is competitively inhibited, preventing the formation of 2‐phosphoglycollate, which is recycled in the energy-demanding photorespiratory pathway instead of the Calvin cycle (Ainsworth and Rogers, 2007). Feedback inhibition of photosynthesis due to elevated carbohydrate levels *via* a hexokinase-dependent mechanism of glucose sensing (Rolland et al., 2006) does not occur in the VOC-exposed plants, possibly implicating the downregulation of ABA signal transduction (Zhang et al., 2008b) and increased cytokinin levels (Sánchez-López et al., 2016b). The increased sucrose and cytokinin levels due to volatile treatment induce the expression of anthocyanin biosynthesis genes through the activation of specific transcription factors of the MYB-bHLH-WD40 (MBW) regulatory complexes, exemplifying the intimate interplay between photosynthesis, carbohydrate-phytohormone metabolism/signaling and gene expression. CK, cytokinin; IAA, indole-3-acetic acid; GAP, glyceraldehyde 3‐phosphate; MEP, methylerythritol 4‐phosphate; ROS, reactive oxygen species; RuBP, ribulose-1,5-biphosphate.

In conclusion, we have shown that respiratory CO_2_ combined with other volatile compounds emitted by *Serendipita* strains are able to promote shoot growth and modify root architecture. In **Figure 11**, our data are integrated into the model proposed by García-Gómez et al. (2019) to provide a tentative mode of action of the *Serendipita* VOCs. This model involves altered gene expression, enhanced photosynthetic efficiency and photoprotection (ROS scavengers, anthocyanin), and modulation of the intimately linked carbohydrate and phytohormone signaling pathways. More specifically, *Serendipita* VOCs and respiratory CO_2_ strongly stimulate the photosynthetic reactions resulting in elevated levels of the Calvin cycle intermediate glyceraldehyde 3‐phosphate (GAP), which leads to higher sugar concentrations and accumulation of methylerythritol 4‐phosphate (MEP) pathway-derived isoprenoid compounds, including cytokinin and ABA. These alterations in turn modulate phytohormone signaling and transcriptional regulation in the plant. The impact of elevated CO_2_ on photosynthetic activity is especially linked to an enhanced CO_2_ fixation through maximization of the carboxylation rates of RuBP by Rubisco (Ainsworth and Rogers, 2007). It is further assumed that feedback inhibition of photosynthesis *via* a hexokinase-dependent mechanism of glucose sensing (Rolland et al., 2006) does not take place, possibly as a result of the downregulation of ABA signal transduction (Zhang et al., 2008a) and increased cytokinin levels (Sánchez-López et al., 2016a). By extending the plethora of diffusible bioactive molecules with volatiles, we uncovered a new mechanism by which the ubiquitous Serendipitaceae, renowned for their beneficial interactions with a broad spectrum of plants, can modify plant development. Although we are still far from translating this knowledge into practice, it opens new perspectives on their implementation within a sustainable land management approach.

## Data Availability Statement

The raw data supporting the conclusions of this article will be made available by the authors upon request to any qualified researcher, without undue reservation.

## Author Contributions

JoV designed and performed the experiments and data analysis with contributions of HL, CW, and LV concerning volatile profiling and treatments. KA and MA assisted in evaluating plant physiological parameters *via* automated plant phenotyping. JaV collected the *Arabidopsis* root length data for the *in vitro* experiments in vertical plates by the use of a dedicated software tool written in Python. KS provided guidance and equipment for the CO_2_-fertilization experiment. JoV and DV wrote the initial draft of the manuscript with final contributions from all co-authors. DV and GH co-designed and directed the research.

## Funding

Part of this research was supported by the Hercules funding AUGE/15/08 (PTR-QiTOF instrument) and AUGE/15/17 (automated plant phenotyping platform).

## Conflict of Interest

The authors declare that the research was conducted in the absence of any commercial or financial relationships that could be construed as a potential conflict of interest.
