# Supplementary material for: Respiratory CO2 Combined With a Blend of Volatiles Emitted by Endophytic Serendipita Strains Strongly Stimulate Growth of Arabidopsis Implicating Auxin and Cytokinin Signaling
Source: Front Plant Sci. 2020 Sep 2;11:544435. doi: 10.3389/fpls.2020.544435 (PMC7492573; doi:10.3389/fpls.2020.544435)
Supplement: Supplementary file 1 [file DataSheet_1.pdf]

Supplementary Material

1 Supplementary Materials and Methods

1.1 Additional details on headspace sampling by PTR-TOF-MS and TD-GC-MS and on the processing of the acquired data

Before the start of the measurements, the in- and outlet of the glass system were connected, respectively, to an N_2-flow controlled by a mass flow controller, and to a High-Resolution Quadrupole inlet Proton Transfer Reaction-Time of Flight-Mass Spectrometer (PTR-QiTOF; IONICON Analytik GmbH, Innsbruck, Austria). The headspace of the vessel was flushed with N_2 at a constant flow rate of 300 mL min^{-1}. From the outlet stream, 160 mL min^{-1} entered the drift tube of the PTR-QiTOF apparatus via a heated (80°C) polyether ether ketone inlet and 100 mL min^{-1} was pumped (during 20 min) over a stainless steel sorbent enrichment tube, while the remaining stream was discarded to the fume hood. Each sorbent tube contained Tenax TA 35/60 and Carbotrap 20/40 with a total sorbent mass of 200 mg (volumetric ratio 50/50; Markes, Llantrisant, UK) and were used to pre-concentrate the VOCs prior to analysis by TD-GC-MS. Before sampling, the Tenax/Carbotrap tubes were conditioned for 1 h at 300°C under a helium flow. Desorption of the analytes pre-concentrated on the sorbent was carried out on a Unity Series 2 Thermal Desorption system (Markes) by heating the tubes at 260°C for 10 min. Subsequently, the volatiles were separated on a Focus GC (Thermo Fisher Scientific, Milan, Italy) equipped with a 30 m FactorFour VF-1 ms low bleed bounded phase capillary GC column (Agilent-Varian, Santa Clara, CA, USA; non-polar, 100% dimethylpolysiloxane, internal diameter 0.25 mm, film thickness 1 µm). The GC temperature program as described in Walgraeve et al. (2011) was followed. Masses from m/z 29 to 300 were recorded in full scan mode (200 ms per scan) on a DSQ II Single Quadrupole MS (Thermo Fisher Scientific, Austin, TX, USA) interfaced to the GC and operating at an electron impact energy of 70 eV. The GC-MS transfer line and the MS ion source were kept at 240°C and 220°C, respectively. Data processing was done using XCalibur v2.2 (Thermo Fisher Scientific). Regarding the PTR-TOF instrument, the operating parameters in the drift tube were set at a pressure of 3.70 mbar, a drift temperature of 80°C and a drift voltage of 1000 V, which resulted in an electric field strength (E) to gas number density (N) ratio of 144 Td (1 Td = 1 Townsend = 10^{-17} V cm^{-2}). Volatile compounds passing the drift tube, having a proton affinity higher than that of water (165.2 kcal mol^{-1} or 691 kJ mol^{-1}), were ionized via proton transfer reactions with H_3O^+ ions generated in the ion source of the PTR-TOF instrument. The resulting product ions were extracted from the drift tube and pulsed every 40 µs to the time-of-flight region, where they were separated according to their mass-to-charge (m/z) ratio (Jordan et al., 2009; Graus et al., 2010). All molecules within the acquired mass spectrum, ranging up to m/z 510, were typically detected at their protonated mass. The mass scale was calibrated using $H_3{}^{18}O^+$ (m/z 21.022), $C_6H_5I^+$ (m/z 203.943) and $C_6H_5I_2^+$ (m/z 330.848). The latter two ions originate from the 1,3-diiodobenzene internal standard, continuously supplied by the built-in PerMaSCal unit. The mass resolution was at least 5000 $m/\Delta(m)_{FWHM}$ for m/z > 30.

As for the processing of the raw data in the PTR-MS Viewer, peak areas were calculated by integrating all ion counts per peak for each compound of the custom-defined peak table having an m/z ratio between 15.993 and 300.066. The peak table was constructed by inspecting the mass

spectrum of all data files and manually setting the center and border positions of each detected spectral peak based on the average signal of the first 200 cycles (seconds) of the sample measurements. To separate overlapping neighboring peaks, two advanced modes for peak integration were applied according to the software's guidelines: *Gauss Mode* and *Multipeak Mode*. The raw data were further corrected for transmission, and peak areas of each of the 2700 retrieved cycles per sample were automatically converted into concentrations expressed in parts per billion by volume (ppbv). This automatic conversion was run according to the formula described by Lindinger et al. (1998), using the primary ions H_3O^+ and $(H_2O)_2H^+$ measured via their corresponding ^{18}O isotopologues at m/z 21.022 and m/z 39.033, respectively, and assuming a constant reaction rate coefficient k of 2×10^{-9} cm^3 s^{-1}.

2 Supplementary Tables

Table S1. Overview of the different *Arabidopsis* mutant and transgenic lines used in this study. All lines were derived from the Col-0 ecotype, except for *tir1-1 afb2-1 afb3-1* and the chlorophyll mutants, which had a mixed Col-0 (*tir1-1*)/Ws (*afb2-1 afb3-1*) and Nossen (*apg2*, *apg3*) or Ws background (*cla1*), respectively.

Signaling pathway/ developmental process	Mutant or transgenic line	Names of defective genes, promotors, and transgenes	Putative function of gene product/promotor	References
Abscisic acid (ABA)	ABA INSENSITIVE 3 (*abi3*) mutant	*ABI3*	Transcription factor essential in mediating ABA actions	Koornneef et al., 1984
Auxin	Triple *tir1/afb* mutant *tir1-1 afb2-1 afb3-1*	F-box protein encoding genes *TIR1*, *AFB2-1*, *AFB3-1*	Auxin receptors enabling the transcription of auxin-regulated genes by facilitating ubiquitination-based degradation of transcriptional repressors	Dharmasiri et al., 2005; Parry et al., 2009
	DR5::GUS reporter line	*DR5* promotor	Highly active synthetic auxin response element	Ulmasov et al., 1997
Cytokinin (CK)	*ahk* double mutants *ahk2 ahk3*, *ahk2 ahk4* and *ahk3 ahk4*	*AHK2-AHK3*, *AHK2-CRE1/AHK4* and *AHK3-CRE1/AHK4*	Histidine kinase receptors binding CK as the first step in the CK phosphorelay signaling system	Riefler et al., 2006
	ARR5::GUS reporter line	Promotor of the *Arabidopsis* response regulator gene *ARR5*	CK-inducible promotor involved in determining the CK signaling output and controlling the activity of the CK signaling pathway	D'Agostino et al., 2000
Ethylene (ET)	ET RESPONSE/ RESISTANT 1 (*etr1*) mutant	*ETR1*	ET receptor actively repressing the signaling response in the absence of ET (ET binding relieves blockage)	Chang et al., 1993
	ET INSENSITIVE 2 (*ein2*) mutant	*EIN2*	Positive regulator of the pathway (downstream of ETR1) enabling signal transduction to the down-stream components	Alonso et al., 1999

**Signaling pathway/ developmental process**	**Mutant or transgenic line**	**Names of defective genes, promotors, and transgenes**	**Putative function of gene product/promotor**	**References**
Salicylic acid (SA)	SA INDUCTION DEFICIENT (*sid2*) mutant	*SID2/ICS1*	Protein with isochorismate synthase activity required to accumulate SA (e.g., after pathogen infection)	Wildermuth et al., 2001
	SA insensitive *NahG* transgenic line	*nahG* transgene	Protein with salicylate hydroxylase activity, degrading SA to catechol	Friedrich et al., 1995
Cell division (mitotic activity)	*CYCB1;1::GUS* reporter line	Promotor of the cyclin gene *CYCB1;1*	Promotor involved in cell cycle progression (mitosis): peak activity during the G2/M phase transition	Colón-Carmona et al., 1999
Chloroplast development	*albino or pale green 2* (*apg2*)[1]	*APG2/cpTatC* (encoding 'chloroplast TatC', a TatC homologue of the *Escherichia coli* delta-pH dependent protein transporter)	Protein serving as an important component in the ΔpH-dependent protein transport system in chloro-plast development (channel protein) and having an essential role in thylakoid membrane formation	Motohashi et al., 2001
	albino or pale green 2 (*apg3*)[1]	*APG3/AtcpRF1* (encoding '*A. thaliana* chloroplast ribosome release factor (RF) 1', an orthologue of *E. coli* RF1)	Protein functioning as part of the chloroplast translation machinery and having an important role in chloroplast development and thylakoid biogenesis	Motohashi et al., 2007
	cloroplastos alterados1 (*cla1*)[1]	*CLA1*	Protein functioning as the first enzyme (1-deoxy-D-xylulose-5-phosphate (DXP) synthase) of the 2-*C*-methyl-D-erythritol-4-phosphate (MEP) pathway for isoprenoid biosynthesis; required for proper chloroplast/etioplast development	Mandel et al., 1996; Estévez et al., 2000

[1]*apg2* and *cla1* plastids do not contain thylakoid membranes, while those of *apg3* mutants have immature thylakoid membranes; as these chloroplast-deficient mutants are hampered in photosynthesis, they are also insensitive to elevated CO_2 levels and were therefore used to study the plant growth-promoting effects of *Serendipita* volatiles other than CO_2.

Table S2. Overview of the different media that were used to study the impact of nutrient availability in the fungal medium on VOC-mediated effects in *A. thaliana*.

Medium	Composition
Complex medium (CM; modified *Aspergillus* medium; Pham et al., 2004)	50 mL L^{-1} 20x salt solution**[1]**, 20 g L^{-1} glucose, 2 g L^{-1} peptone, 1 g L^{-1} yeast extract, 1 g L^{-1} casein hydrolysate, 1 mL L^{-1} microelements**[2]**, and 15 g L^{-1} agar; pH 6.4
Malt extract agar (MEA)	CM0059, Thermo Fisher Scientific-Oxoid Limited, Basingstoke, UK
Malt yeast peptone (MYP)	7.0 g L^{-1} malt extract, 1.0 g L^{-1} peptone, 0.5 g L^{-1} yeast extract and 15 g L^{-1} agar; pH 5.8
Murashige and Skoog (MS), half-strength (+1% sucrose)	2.2 g L^{-1} MS salts including vitamins, 0.5 g L^{-1} MES monohydrate, 0.1 g L^{-1} myo-inositol and 7.0 g L^{-1} Phyto agar (10 g L^{-1} sucrose); pH 5.7.
Plant nutrition medium, modified (PNM; Sherameti et al., 2008; Johnson et al., 2011)	0.50 g L^{-1} KNO_3, 0.50 g L^{-1} $MgSO_4.7H_2O$, 0.33 g L^{-1} $Ca(NO_3)_2$, 2.5 mL L^{-1} Fe-EDTA**[3]**, 1.0 mL L^{-1} microelements**[4]**, and 15 g L^{-1} agar; pH 5.6 (adjusted with 2.5 mL 1 M KH_2PO_4)
Potato dextrose agar (PDA)	CM0139, Thermo Fisher Scientific-Oxoid Limited, Basingstoke, UK

[1]*CM 20x salt solution*: 120 g L^{-1} $NaNO_3$, 10.4 g L^{-1} KCl, 10.4 g L^{-1} $MgSO_4.7H_2O$, 30.4 g L^{-1} KH_2PO_4

[2]*CM microelement solution*: 6.00 g L^{-1} $MnCl_2.4H_2O$, 1.50 g L^{-1} H_3BO_3, 2.65 g L^{-1} $ZnSO_4.7H_2O$, 0.75 g L^{-1} KI, 2.40 mg L^{-1} $Na_2MoO_4.2H_2O$, 0.13 g L^{-1} $CuSO_4.5H_2O$

[3]*PNM Fe-EDTA solution*: 5.5 g L^{-1} $FeSO_4.7H_2O$, 7.5 g L^{-1} $Na_2EDTA.2H_2O$ (heated in microwave and stirred for 30 min while cooling)

[4]*PNM microelement solution*: 2.77 g L^{-1} $MnCl_2.4H_2O$, 4.33 g L^{-1} H_3BO_3, 0.29 g L^{-1} $ZnSO_4.7H_2O$, 48.39 mg L^{-1} $Na_2MoO_4.2H_2O$, 0.12 g L^{-1} $CuSO_4.5H_2O$, 0.58 g L^{-1} NaCl, 2.38 mg L^{-1} $CoCl_2.6H_2O$

3 Supplementary Figures

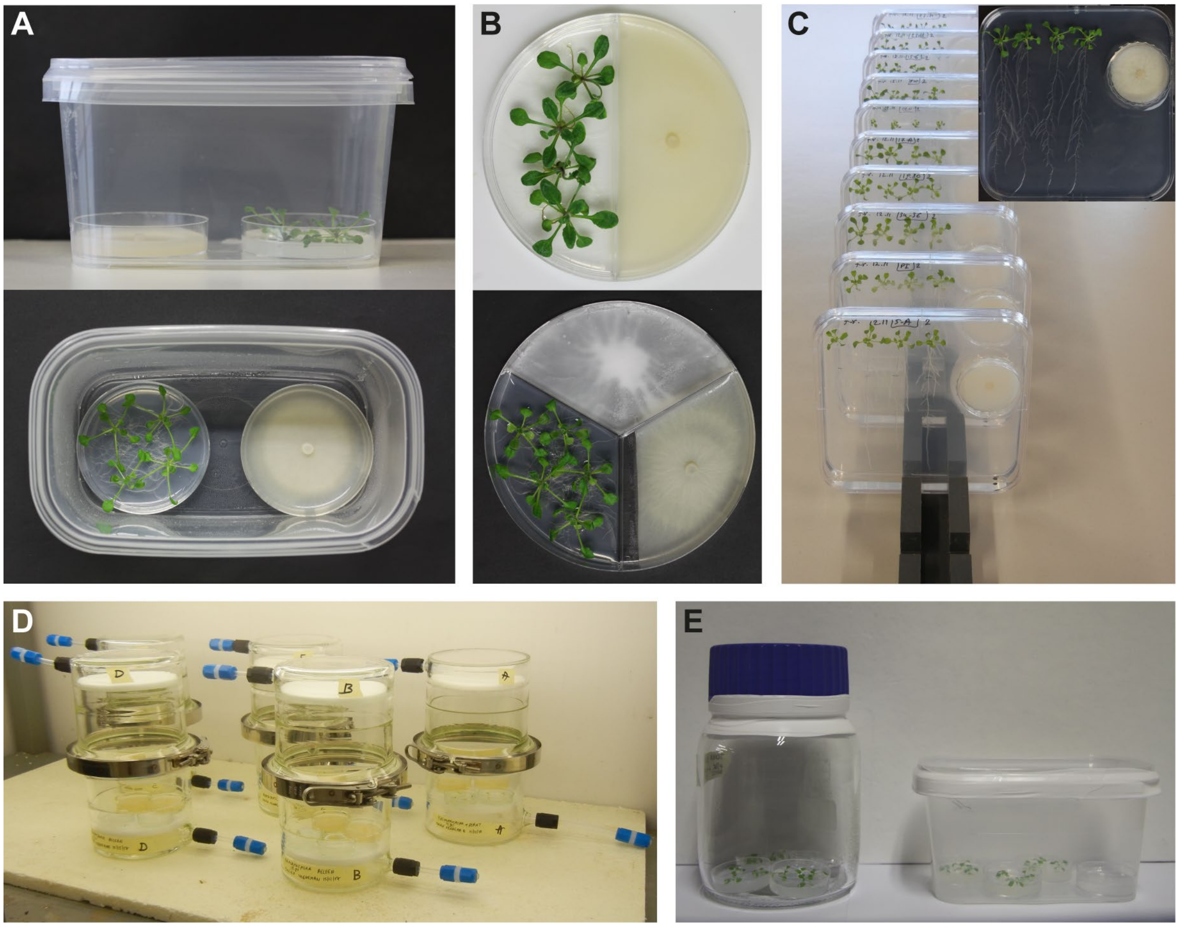

Figure S1. Overview of the different volatile test systems used in this study. **(A)** ***Petri-dish-in-box*** setup in side and top view. Plant and fungal cultures are physically separated but share the same headspace. **(B)** ***I- or Y-split-plate*** bioassays with compartmentalized petri dishes in which plant and fungus are separated by a plastic barrier inside the plate; top: regular volatile co-cultivation experiment; bottom: CO_2-tapping experiment with $Ba(OH)_2$ in the third compartment. **(C)** ***Vertical-plate*** assay with square petri dishes containing a small fungal plate fixed inside the plant medium. **(D)** ***Glass reaction vessels*** consisting of two joined units, each with an in- and outlet, used to measure *Serendipita* respiratory CO_2 production, to fertilize plants with external CO_2, to determine the fungal VOC profile via PTR-TOF-MS, and to treat plants with pure volatile compounds. The two units of the vessel were connected by 120-NW flat flanges at their open ends using an airtight seal consisting of a Bola silicone ring with FEP coating (Bohlender GmbH, Grünsfeld, Germany), fitting into the O-shaped grooves of the flanges, and a stainless steel quick release clamp (Duran, DWK Life Sciences, Mainz, Germany). Both vessel halves contained a sintered glass filter near their in-/outlet. The latter carried GL 14 threads and was connected to PFA tubing of 6.35 mm (1/4") outer diameter (Swagelok, Solon, OH, USA) via a Bola PPS-PTFE screw joint. **(E)** Two smaller setups that were initially tested in an attempt to volatilize pure compounds in an enclosed system with plants. Small quantities of the liquid form of the compound were mixed with water inside a separate petri dish. The containers were sealed with a fitting lid or screw cap and some additional layers of Teflon.

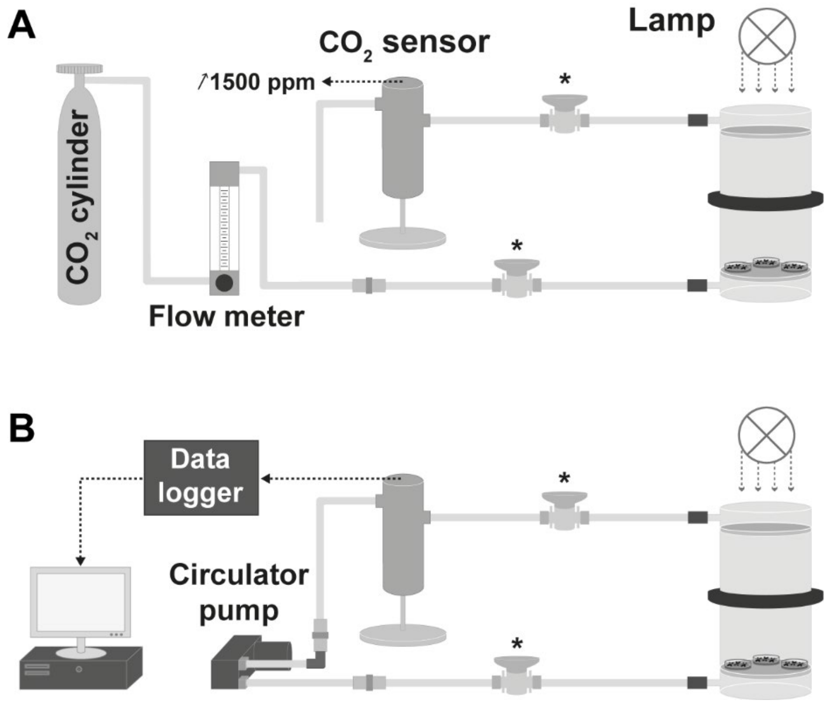

Figure S2. Setup of the CO_2 fertilization experiment. **(A)** An elevated CO_2 level of 1500 ppm was created inside a 4-L glass reaction vessel, containing petri dishes with *Arabidopsis* seedlings, by adding a 99.85/0.15% N_2/CO_2 gas mixture from a compressed gas cylinder. **(B)** Once the required CO_2 level was reached, as indicated by the CO_2 sensor, the two valves (one before the inlet and one behind the outlet of the vessel, marked with *) were temporarily closed in order to disconnect the CO_2 container and attach the tubing to a circulator pump, after which the valves were opened again. The pump ensured a constant gas circulation inside the closed circuit enabling an accurate real-time CO_2 measurement of the air passing through the sensor. Besides the CO_2 treatment, two controls without external gas supply were included: the first control consisted of a closed circuit comparable to the one illustrated in (B), and the second control was restricted to the glass vessel only, meaning no air circulation was present. Additionally, a VOC treatment with *Serendipita* isolate 30 was conducted without air circulation.

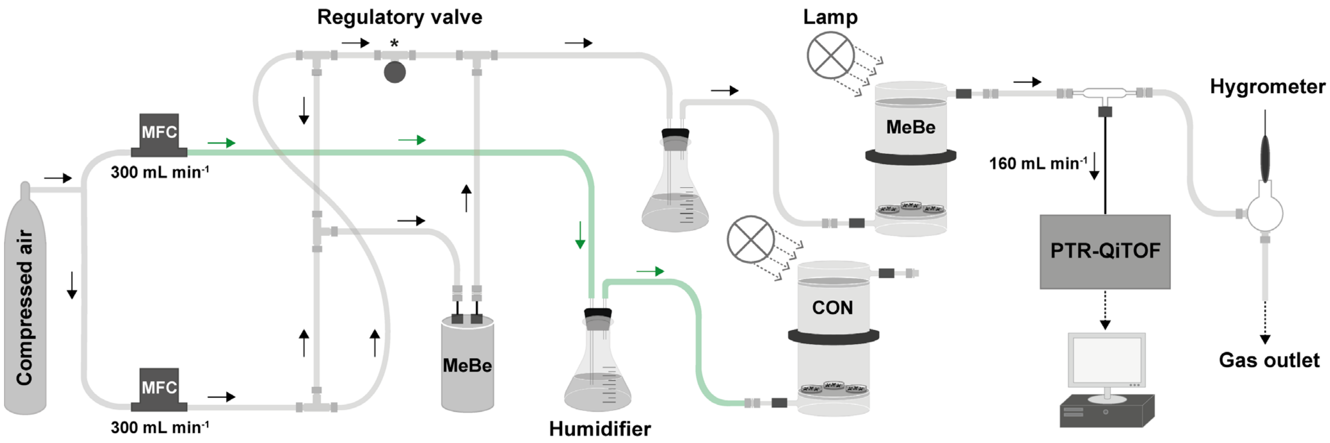

Figure S3. In-house developed methyl benzoate gas-generation system to examine the effects of this volatile compound on *Arabidopsis* growth. Dry air with a constant flow rate of 300 mL min^{-1}, as regulated by a mass flow controller (MFC), was passed through a light-protected container filled with methyl benzoate (MeBe), after which the methyl-benzoate-enriched air was sequentially guided through a humidifier, generating 100% humidity as confirmed by hygrometer measurements, and the 4-L glass vessel containing the plants. The regulatory valve (see asterisk) was used to control the gas-phase methyl benzoate concentration, which was constantly monitored by injecting a portion of the outlet flow (160 mL min^{-1}) into the PTR-QiTOF instrument. A similar trajectory was followed for the control vessel, but the airflow did not pass the MeBe container and was not monitored (indicated by green color).

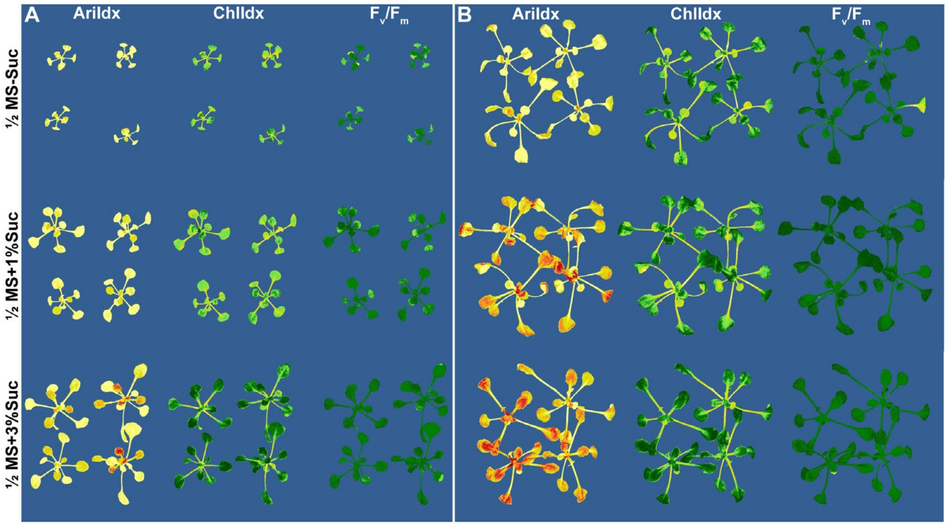

Figure S4. Effect of volatile production of *Serendipita* isolate 30 on spectral and chlorophyll fluorescence parameters in *A. thaliana* grown on ½ MS with varying concentrations of sucrose (0%, 1% and 3%) in a petri-dish-in-box-assay. The spatial distribution of the recorded pixels across the different classes of the parameters anthocyanin accumulation (AriIdx), chlorophyll content (ChlIdx) and F_v/F_m ratio is visualized in control (A) and VOC-treated (B) *Arabidopsis* plants. Images were obtained via the automated plant phenotyping platform and correspond to the RGB images in Figure 1A. For the color codes we refer to the legend of the different classes as shown in the stacked bar charts of the respective parameters (Figure 1C-E). MS, Murashige and Skoog medium; Suc, Sucrose.

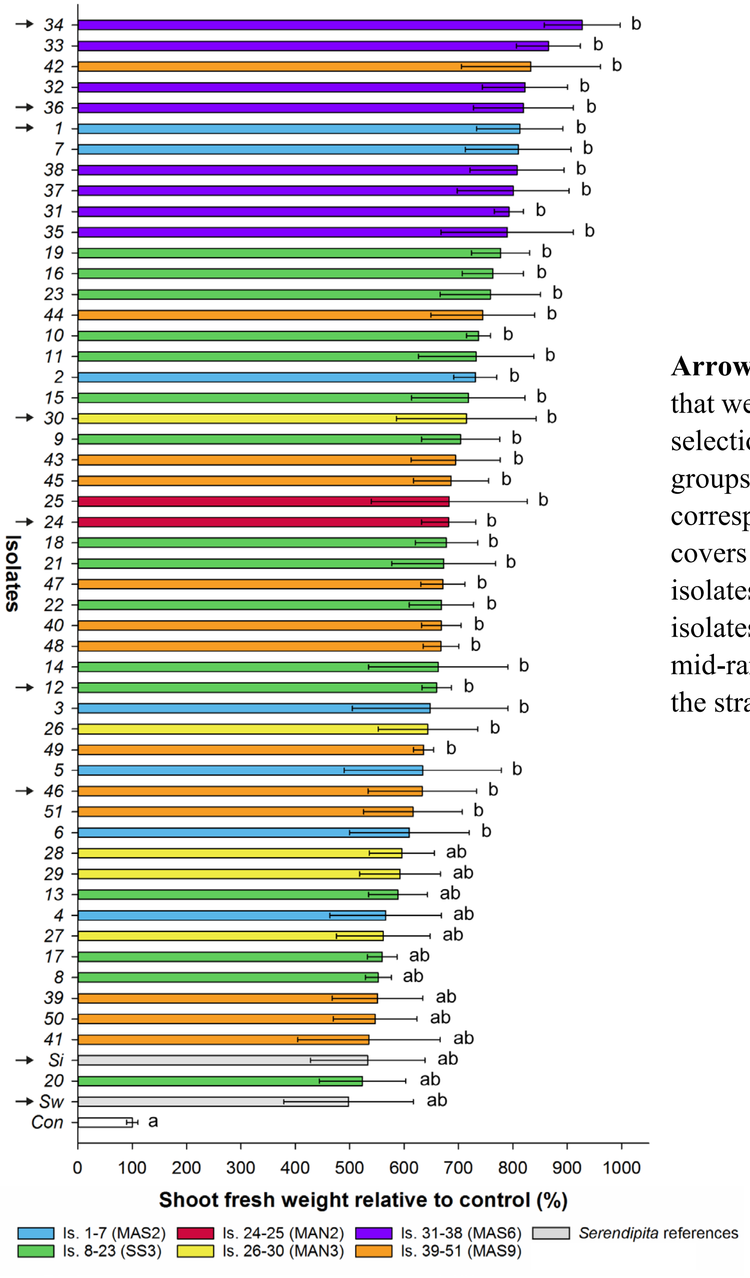

Arrows indicate the subset of *Serendipita* isolates that were chosen for subsequent experiments. This selection also represents the different 'genetic groups' present in the collection, which largely correspond to the original sampling locations, and covers the entire spectrum of growth promotion: isolates 34, 36 and 1 represent the most conducive isolates, isolates 30, 24 and 12 are situated in the mid-range, and isolates 46, *Si* and *Sw* rank among the strains with the smallest effects.

Sampling locations Congo (Venneman et al., 2017):

MAN2, MAN3 = Mandombe sites 2 and 3

MAS2, MAS6, MAS9 = Masako sites 2, 6 and 9

SS3 = Simi-Simi site 3

Figure S5. Mean shoot fresh weight, expressed relative to the control, of *A. thaliana* after ten days of co-cultivation with each of the 53 available *Serendipita* strains (51 from Congolese collection plus the two references *Si* and *Sw*) in a split-plate assay. Isolates originating from the same sampling location in Congo are indicated by the same color. Error bars show standard errors on the mean of four replicates (four independent repetitions with one biological replicate each). Treatments sharing the same letter are not significantly different according to a Tukey HSD post-hoc test ($\alpha = 0.05$). Based on a separate analysis with data grouped per location, the origin of the isolates did not seem to determine the extent of the observed plant effects, with the exception of the strains obtained from site 6 in Masako (isolates 31-38), which performed better than the isolates from any other sampling location in the DRC (Tukey HSD post-hoc test, $P < 0.05$ when comparing MAS6 with the other locations). Con, Control; Is., Isolate; *Si*, *S. indica*; *Sw*, *S. williamsii*.

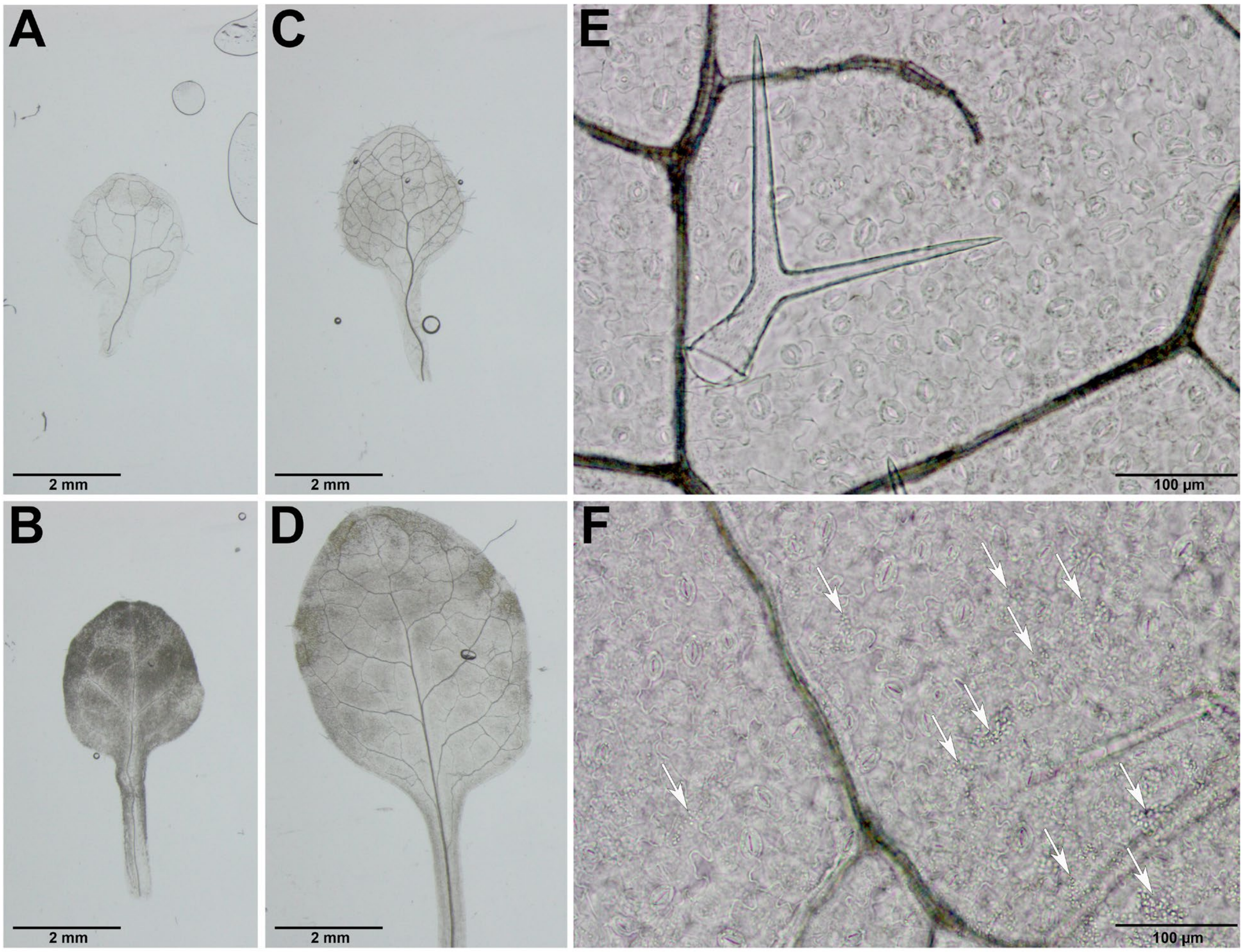

Figure S6. *Serendipita* VOC-mediated starch granule accumulation in *A. thaliana* plants grown in split-plate assays. Plants were grown on ½ MS without sucrose and *Serendipita* isolate 30 on PDA **(A,B)** After 4 days of volatile exposure, clearing of representative leaves revealed a darker color in VOC-treated (B) compared to control plants (A). **(C,D)** Same as (A,B) after 8 days of VOC exposure. **(E,F)** Microscopy images of the leaves in (C, D) show an accumulation of granules (indicated by arrows) upon VOC exposure (F), likely representing amyloplasts, that is not observed in the control leaves (E).

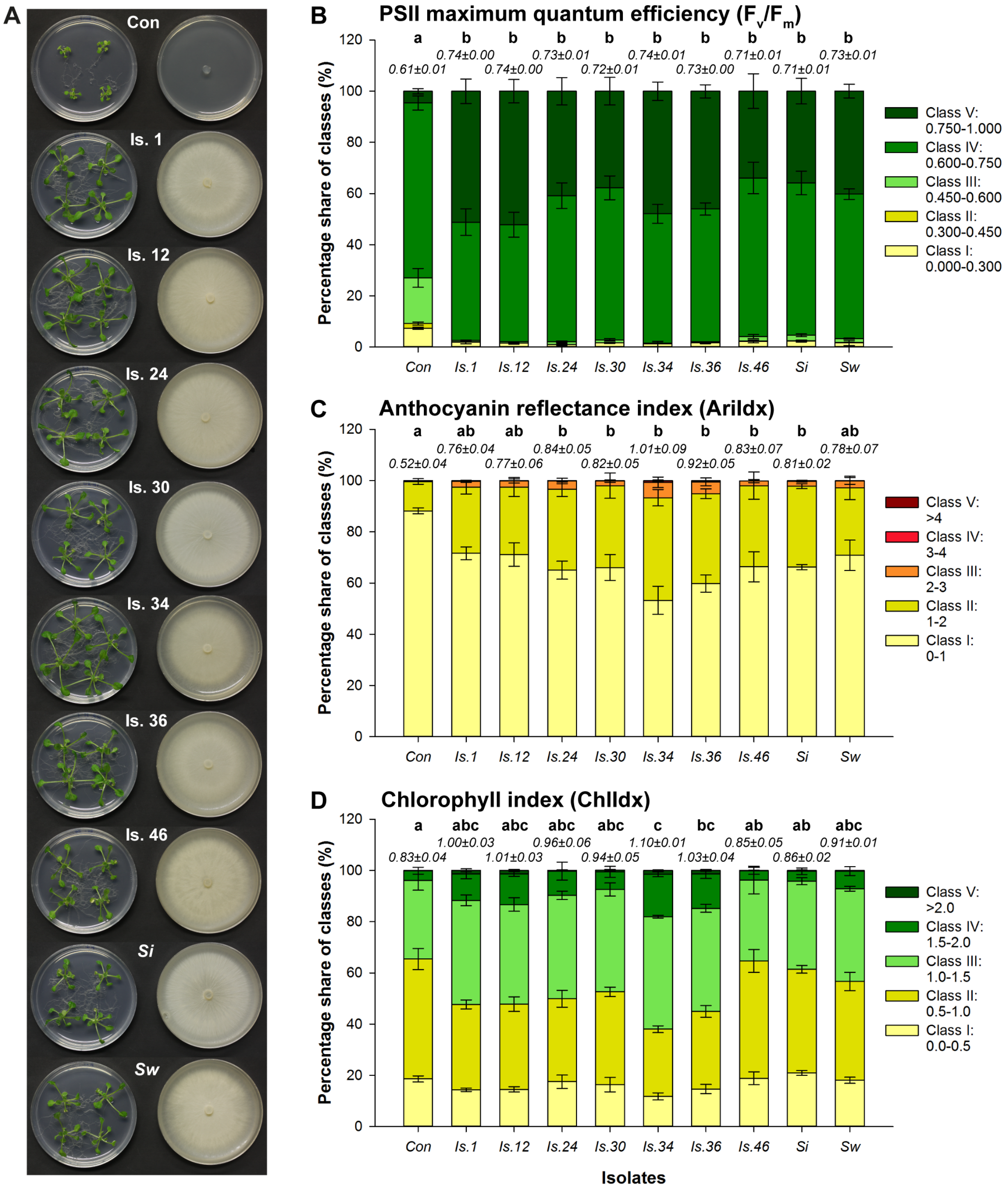

Figure S7. VOC-mediated effects in *A. thaliana* after co-cultivation with the selected *Serendipita* isolates in a petri-dish-in-box assay. **(A)** Observed plant growth responses after ten days of co-cultivation. **(B-D)** Distribution of the recorded pixels (obtained via plant phenotyping platform) across different classes for the chlorophyll fluorescence parameter F_v/F_m (B) and the spectral parameters anthocyanin accumulation (AriIdx, C) and chlorophyll content (ChlIdx, D). Average total parameter values (± standard errors) are shown for each treatment above the respective stacked bar. Error bars represent standard errors on the mean of three biological replicates. Treatments sharing the same letter are not significantly different according to a Tukey HSD post-hoc test (α = 0.05). Con, Control; Is., Isolate; *Si*, *S. indica*; *Sw*, *S. williamsii*.

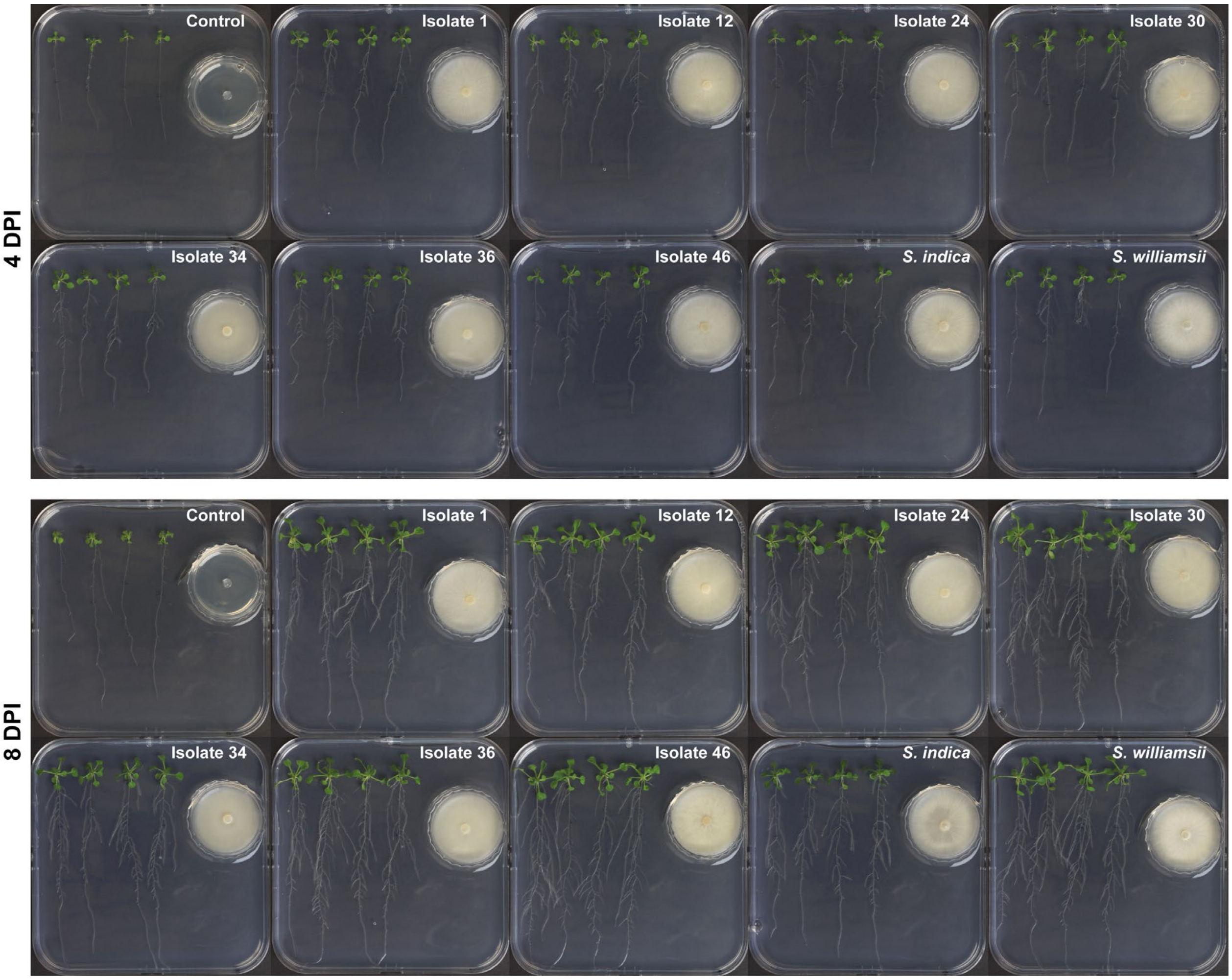

Figure S8. Vertical-plate assay to examine the VOC-mediated effects of a selection of *Serendipita* isolates (1, 12, 24, 30, 34, 36, 46, *S. indica* and *S. williamsii*) on root development in *Arabidopsis*. Root architecture and shoot growth were evaluated after four and eight days of co-cultivation. DPI, days post 'inoculation' (start of co-cultivation).

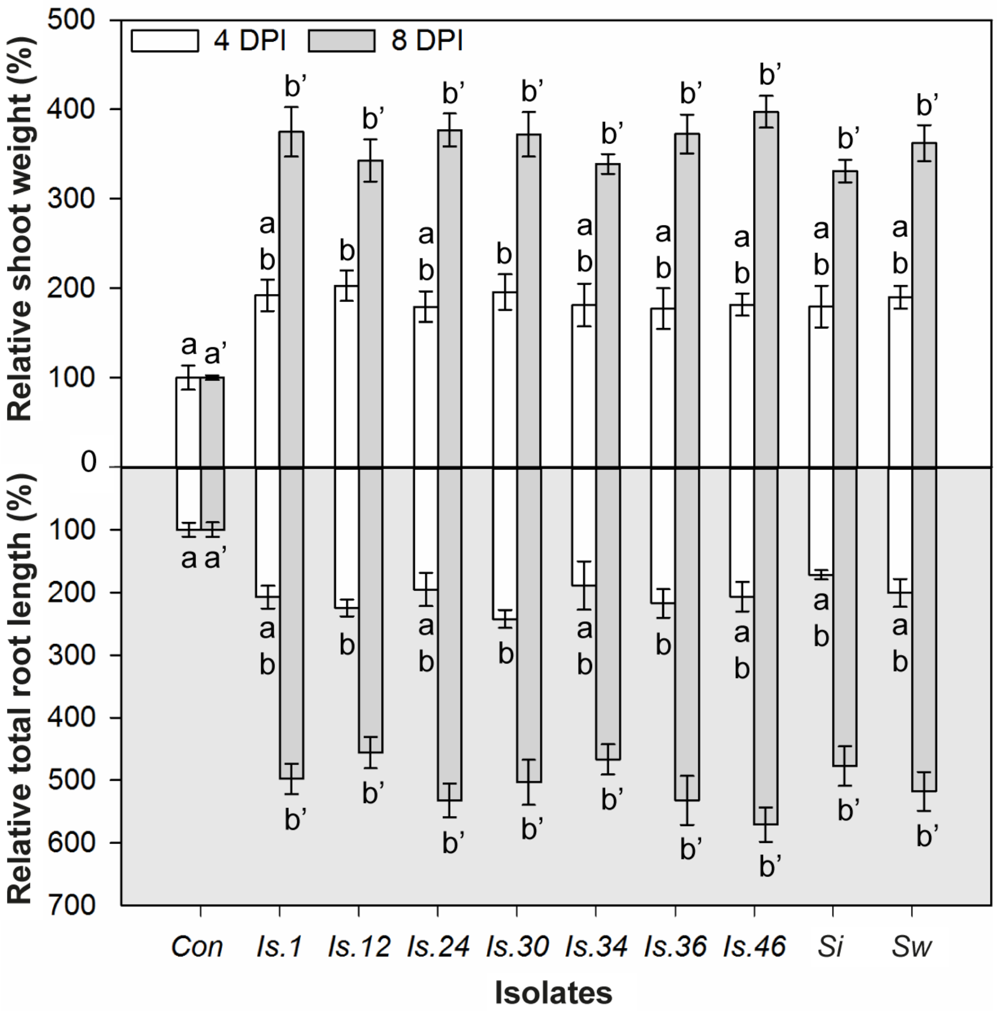

Figure S9. Average shoot fresh weight and total root length (relative to control) of the vertically grown *Arabidopsis* plants shown in Supplementary Figure S7, measured after four and eight days of *Serendipita* VOC exposure. Error bars indicate standard errors on the mean of the different treatments, based on three (4 DPI) or five (8 DPI) replicates. For both evaluation moments, shoot weight and root length were compared across the different treatments using Tukey HSD post-hoc tests ($\alpha = 0.05$), performed after a significant ANOVA test ($\alpha = 0.05$); treatments sharing the same letter above or below their respective bars do not exhibit statistically significant differences. Note that although no significant differences between the isolates can be detected, the trend in the extent of the PGP effect across isolates appears to be similar in shoots and roots, corroborating our previous findings from direct-contact experiments (see Venneman et al., 2017). The data on shoot fresh weight are also incorporated in Supplementary Figure S9 where they are compared with the data of the other experimental setups. Con, Control; DPI, days post 'inoculation' (start of co-cultivation); Is., Isolate; *Si*, *Serendipita indica*; *Sw*, *Serendipita williamsii*.

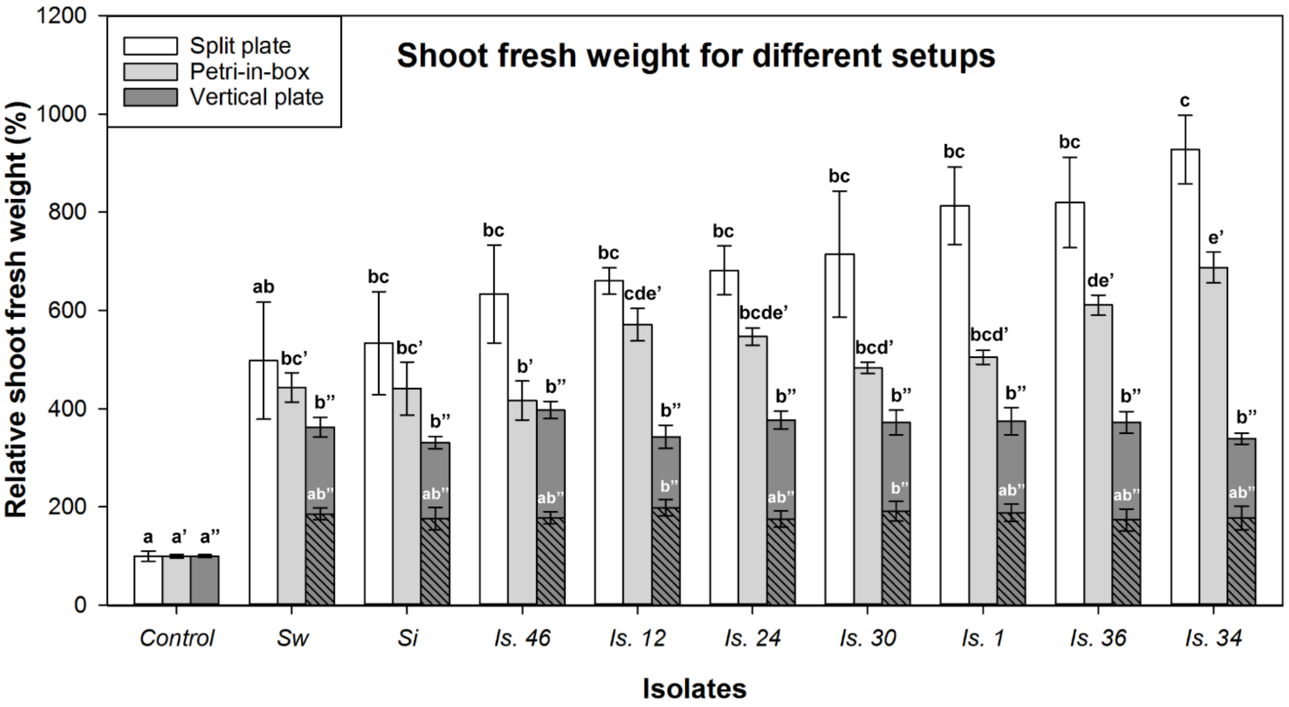

Figure S10. Comparison of the average shoot fresh weights, expressed relative to the control, recorded for *A. thaliana* after *Serendipita* VOC treatment in split plates (I-plates), boxes and vertical plates (10, 10 and 8 days of exposure, respectively). Isolates are ranked in ascending order of their plant growth effect as observed in the split-plate experiment. For the vertical-plate assay, the data obtained after four days of co-cultivation are shown as well (see hatched bars). The split-plate data were derived from four independent experiments with one biological replicate each, while the results of the assay in boxes were retrieved from a single experiment with three biological replicates. The vertical-plate assay was based on three (4 days of co-cultivation) or five (8 days) replicates coming from one and three independent experiments, respectively. Error bars on the graph represent standard errors on the mean of the different treatments. Treatments sharing the same letter above their respective bars are not significantly different according to Tukey HSD post-hoc tests ($\alpha = 0.05$), performed after a significant ANOVA test ($\alpha = 0.05$). When comparing the shoot fresh weight data from the three experimental setups, it is clear that robust growth responses to *Serendipita* VOCs are obtained independent of the system used, although the magnitude of this volatile-mediated response and the ranking of the isolates with respect to the extent of their VOC effect might slightly differ. Nevertheless, whereas no significant differences were recorded amongst the *Serendipita* strains in the vertical assay, isolate 46, *S. indica* and *S. williamsii* were consistently positioned among the least performing isolates in the split-plate and petri-in-box assays, as opposed to isolates 34 and 36, which always outperformed the other tested strains. Is., Isolate; *Si*, *Serendipita indica*; *Sw*, *Serendipita williamsii*.

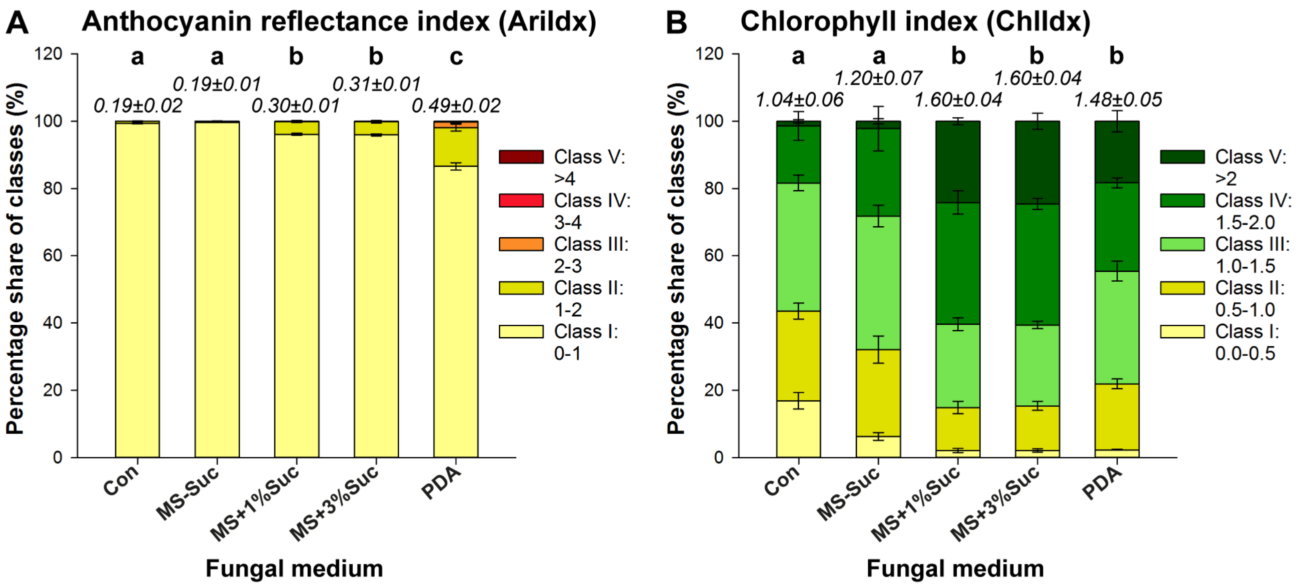

Figure S11. Pixel distribution across five AriIdx (A) and five ChlIdx (B) classes based on spectral imaging of *Arabidopsis* shoots from a petri-dish-in-box assay with *Serendipita* isolate 30 grown on different nutrient sources. Average total values of both AriIdx and ChlIdx for each of the tested conditions are shown above the respective stacked bars (± standard errors). Error bars indicate standard errors on the mean of four replicates; treatments marked with the same letter are not significantly different from each other according to Tukey HSD post-hoc tests ($\alpha = 0.05$). Con, Control; MS, ½ Murashige and Skoog medium; PDA, potato dextrose agar; Suc, Sucrose.

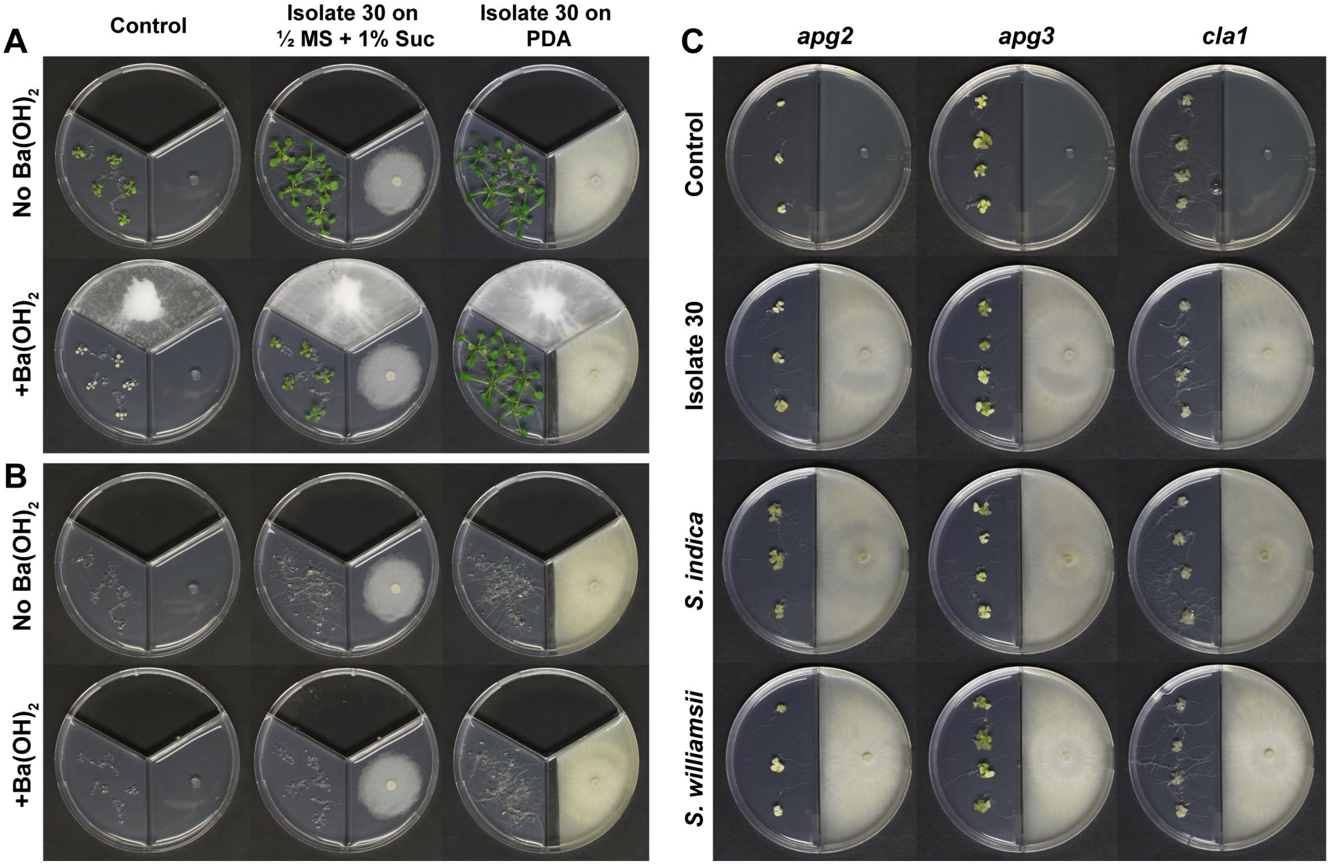

Figure S12. Volatile assays using $Ba(OH)_2$ (A-B) and mutants impaired in chloroplast development (C) to evaluate the impact of *Serendipita* respiratory CO_2 on *Arabidopsis* growth. **(A)** *Arabidopsis* plant growth (on ½ MS without sucrose) as observed after eight days of co-cultivation with *Serendipita* isolate 30 (cultured on PDA and on ½ MS + 1% sucrose) in Y-plates in the presence and absence of 0.1 M $Ba(OH)_2$ as a CO_2 trap. **(B)** Same as (A) but with a better view of the roots (after removal of shoots and $Ba(OH)_2$ solution). **(C)** Responses of the chloroplast mutants *apg2*, *apg3* and *cla1* (on ½ MS supplemented with 1% sucrose) after 14 days of exposure to volatiles from *Serendipita* isolate 30, *S. indica* and *S. williamsii* (on PDA) in I-plates. *apg2*, *albino or pale green 2*; *apg3*, *albino or pale green 3*; *cla1*, *cloroplastos alterados1*; MS, Murashige and Skoog medium; PDA, potato dextrose agar; Suc, Sucrose.

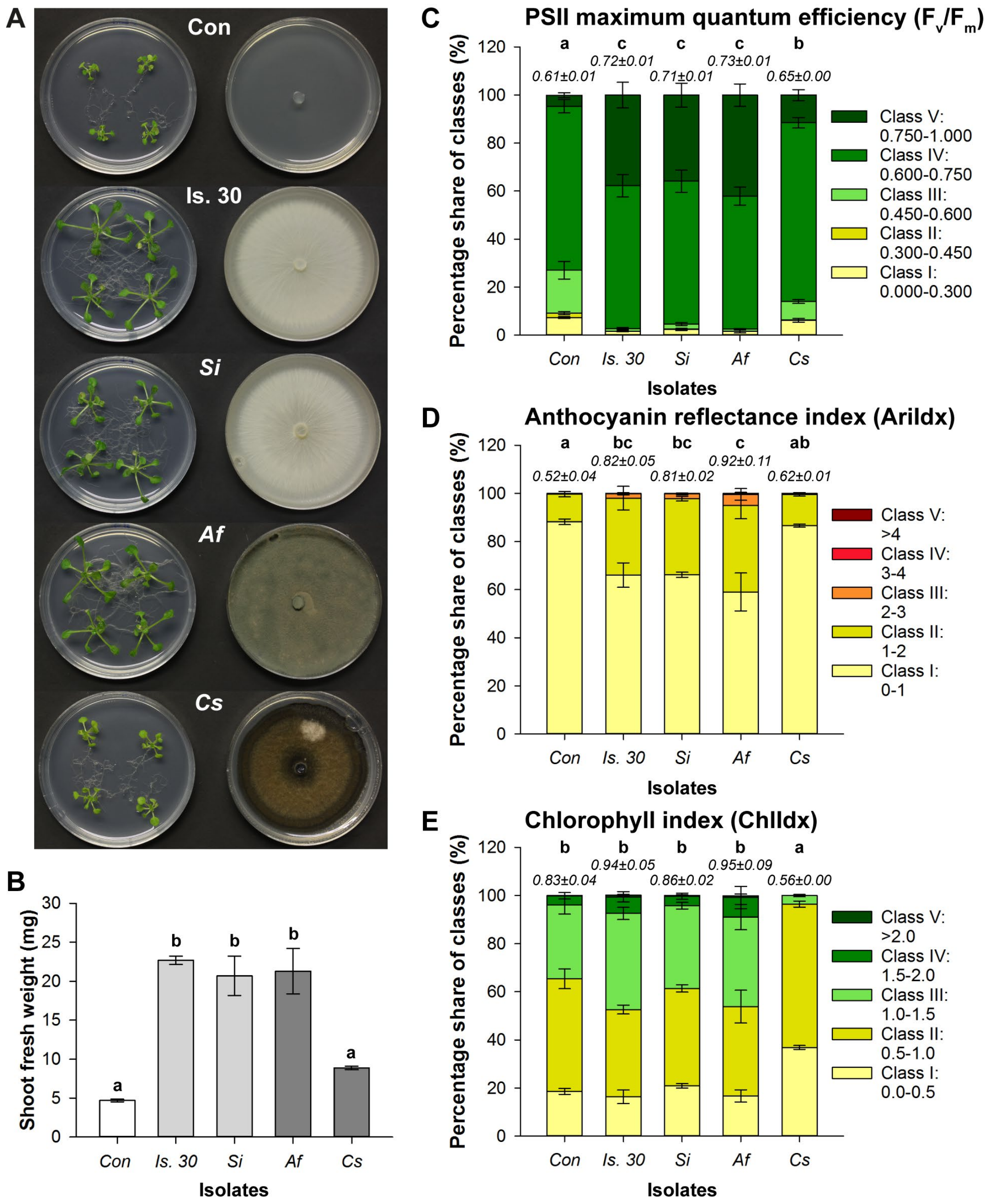

Figure S13. VOC-mediated effects in *A. thaliana* co-cultivated with *Serendipita* isolates compared with the effects caused by exposure to VOCs from saprophytic/pathogenic strains in a petri-dish-in-box assay. **(A)** Observed plant growth responses after ten days of co-cultivation with *Serendipita* isolate 30 (Is. 30), *S. indica* (*Si*), *Aspergillus fumigatus* (*Af*; saprophyte) and *Cochliobolus sativus* (*Cs*; pathogen). **(B)** Average shoot fresh weight of plants from (A) (light grey bar = *Serendipita* strain; dark grey bar = saprophyte/pathogen). **(C-E)** Distribution of the recorded pixels, obtained via chlorophyll fluorescence and multispectral imaging, across five F_v/F_m (C), AriIdx (D) and ChlIdx (E) classes. Average total parameter values (± standard errors) are shown for each treatment above the respective stacked bar. Error bars in all displayed graphs represent standard errors on the mean of three biological replicates. Treatments sharing the same letter are not significantly different according to a Tukey HSD post-hoc test ($\alpha = 0.05$).

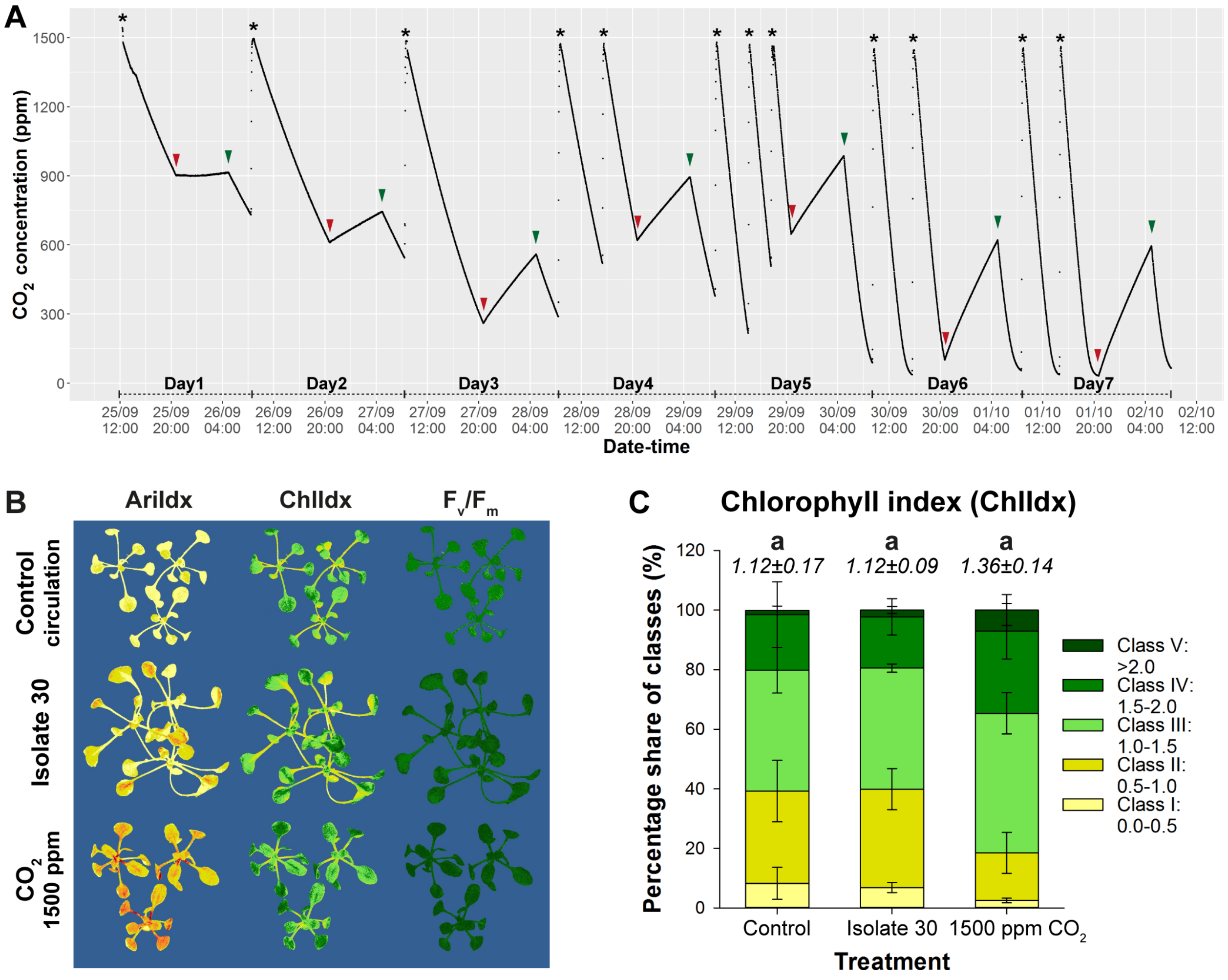

Figure S14. Comparison of the effects of exposure to 1500 ppm CO_2 and those caused by *Serendipita* VOCs on *Arabidopsis* in 4-L glass reaction vessels. **(A)** Daily fluctuations in CO_2 concentration measured in the '1500 ppm CO_2' treatment. The level of 1500 ppm CO_2 was restored one to three times a day (indicated by *). A 16/8 h light/dark photoperiod was maintained; the start of the day (04:30) and night (20:30) cycles are marked with green and red arrowheads, respectively. Note that CO_2 concentrations decreased rapidly during the light period due to photosynthesis, whereas they markedly increased during the dark period because of respiration. **(B)** Visualization of the distribution of the recorded pixels across different classes of the spectral parameters anthocyanin (AriIdx) and chlorophyll (ChlIdx) content, and of the chlorophyll fluorescence parameter F_v/F_m in control and VOC/CO_2-treated plants. For the color codes we refer to the legend of the different classes as shown in the stacked bar charts of the respective parameters (see, e.g., Figures 6C,D). **(C)** Graphical representation of the pixel distribution for chlorophyll content. Error bars indicate standard errors on the mean of three biological replicates. Average total values of the chlorophyll indices (± standard errors) for the evaluated treatments are displayed above the respective stacked bars; they are not statistically different according to ANOVA ($\alpha = 0.05$). When we refer to 'Control' without further specification, the data or images from the control vessel with air circulation are shown.

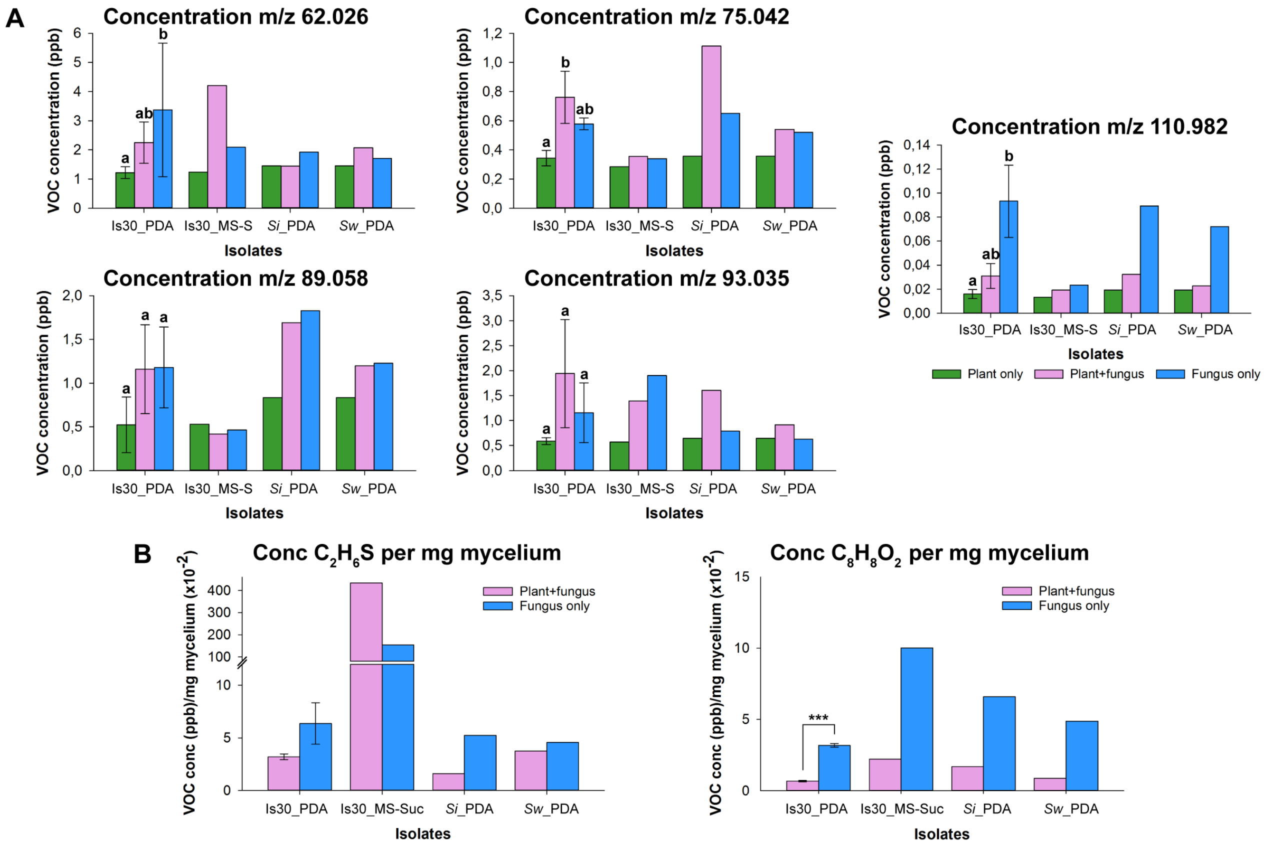

Figure S15. Concentration of volatile compounds present in *Serendipita* VOC mixtures, determined via direct headspace analysis using PTR-TOF-MS. **(A)** Concentrations of the minor VOCs, indicated by their m/z ratios, that were retained after PCA analysis. For each individual analysis, only the signals from 60 consecutive cycles at the beginning of the measurement were used to calculate an average concentration per compound. Color codes indicate whether the vessel contained either plant cultures (**green**), fungal cultures (**blue**) or both (**pink**). Error bars for the experiment with isolate 30 grown on PDA represent standard deviations; treatments sharing the same letter above their respective bars are not significantly different according to a Dunn's test adjusted for multiple comparisons ($\alpha = 0.05$). **(B)** Concentrations of DMS (C_2H_6S) and methyl benzoate ($C_8H_8O_2$) corrected for fungal biomass, corresponding to the data presented in Figures 7B,C. The total mycelial mass per glass vessel was quantified by separating the thin fungal layer from the solid medium, by re-dissolving the latter in a microwave, and weighing the mycelium after overnight drying at 60°C. The dry weight of the fungal mycelium was approximately 50 times higher on PDA than on ½ MS. Asterisks indicate significant differences between the data of isolate 30 (PDA) co-cultivated with plants and those of isolate 30 grown without plants (t-test, *** < 0.001). Is, Isolate; MS, ½ Murashige and Skoog; PDA, potato dextrose agar; S or Suc, Sucrose; *Si*, *Serendipita indica*; *Sw*, *Serendipita williamsii*.

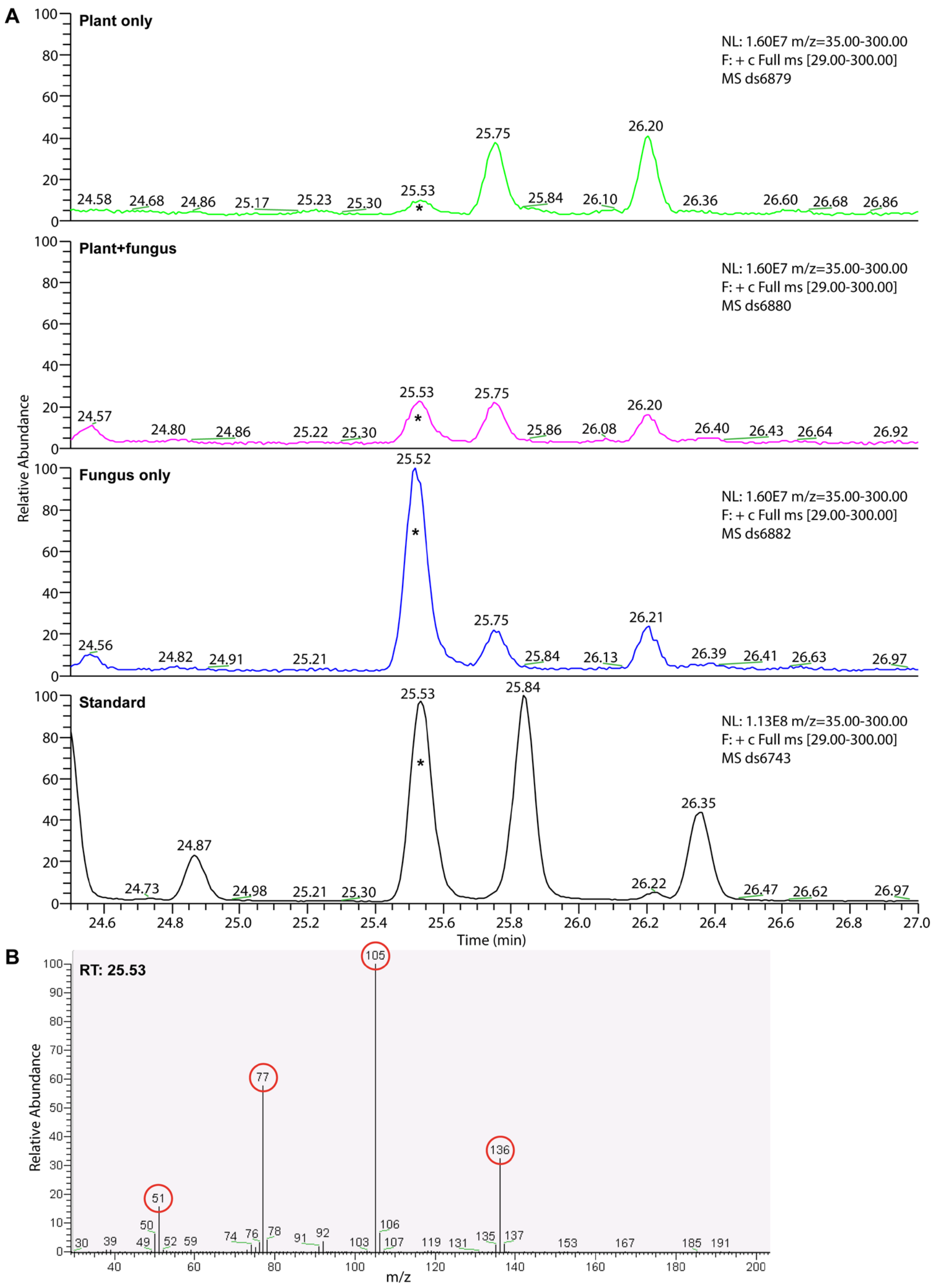

Figure S16. Identification of methyl benzoate in *Serendipita* headspace samples via TD-GC-MS. The identity of methyl benzoate (indicated by asterisks) in the samples was confirmed by comparing the compound's retention time with that of methyl benzoate added as a reference in a liquid standard (A), and by comparing its MS spectrum with the NIST mass spectral library and with the spectrum of the methyl benzoate reference (B). Color codes in (A) indicate whether the analysis was performed on samples from vessels containing either plant cultures (**green**), fungal cultures (**blue**) or both (**pink**), or on the liquid reference standard (**black**). Note that methyl benzoate is detected at much higher concentrations in the fungal treatments (data of isolate 30 grown on PDA) than in the plant control. The graph in (B) displays the characteristic mass spectrum of methyl benzoate (retention time = 25.53 min) obtained in this study, consisting of the main molecule at m/z 136 and the typical fragment ions at m/z 51, m/z 77 and m/z 105.

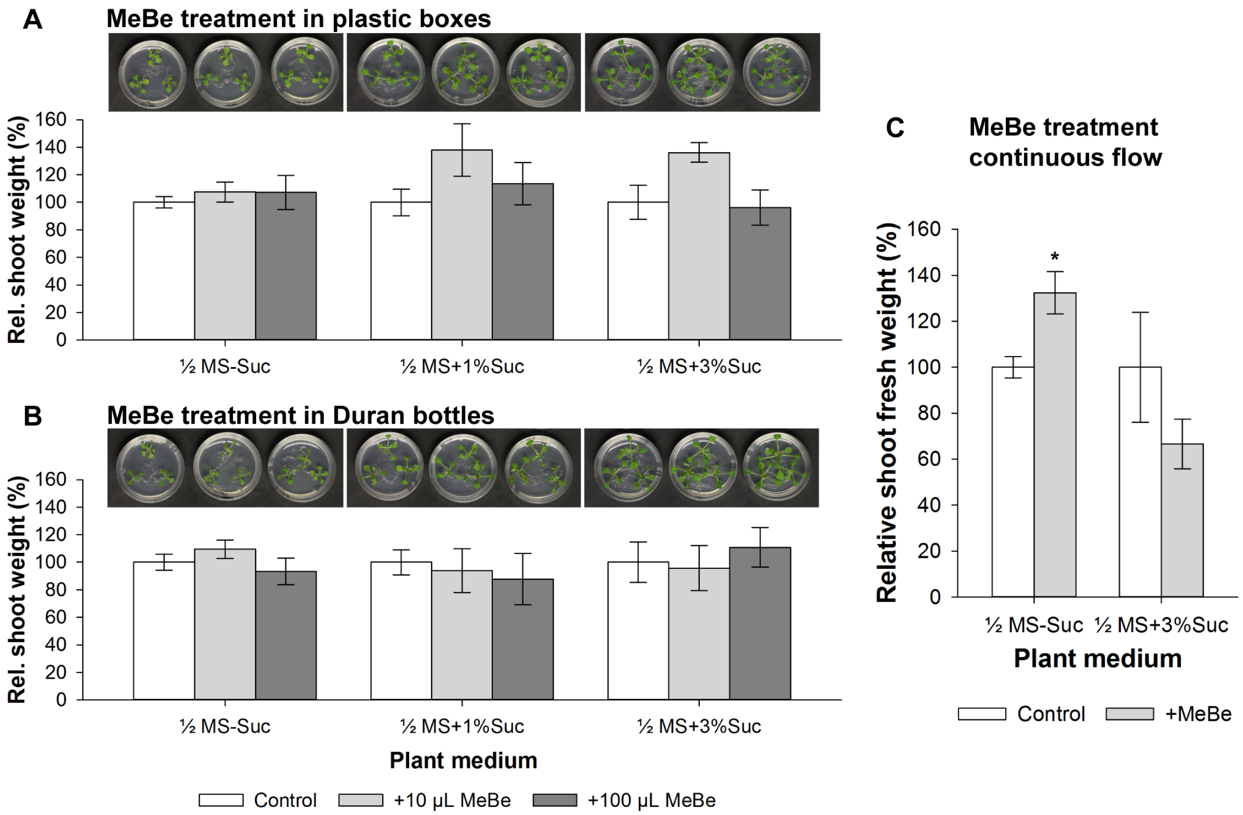

Figure S17. Response of *Arabidopsis* to methyl benzoate (MeBe) applied as an individual compound. **(A-B)** Plant growth and mean shoot fresh weight of *Arabidopsis* plants grown on ½ MS without and with (1% and 3%) sucrose, exposed to either a low or high MeBe application dose (petri dish filled with 5 mL of water spiked with either 10 µL or 100 µL, respectively, of a 0.4-mM MeBe stock solution) in ca. 500-mL plastic boxes (A) or Duran bottles (B), expressed relative to the control. The sucrose-containing media were included to compensate for the lack of CO_2 that is normally produced by the fungus. Photographs above the bars show the growth of the seedlings after seven days. **(C)** Mean relative shoot fresh weight of *Arabidopsis* plants grown on ½ MS without and with 3% sucrose in a 4-L glass reaction vessel and subjected for seven days to a constant flow of either untreated (control) or MeBe enriched carrier gas via a MeBe gas-generation system. Error bars in the displayed graphs indicate standard errors on the mean of three replicates with each replicate representing three plants (per tested plant medium, each container/vessel contained three *Arabidopsis* plates, which were considered as internal biological replicates). Asterisks indicate statistically significant differences compared to the control according to t-tests (two-sided; * < 0.05). MS, Murashige and Skoog medium; Suc, Sucrose.

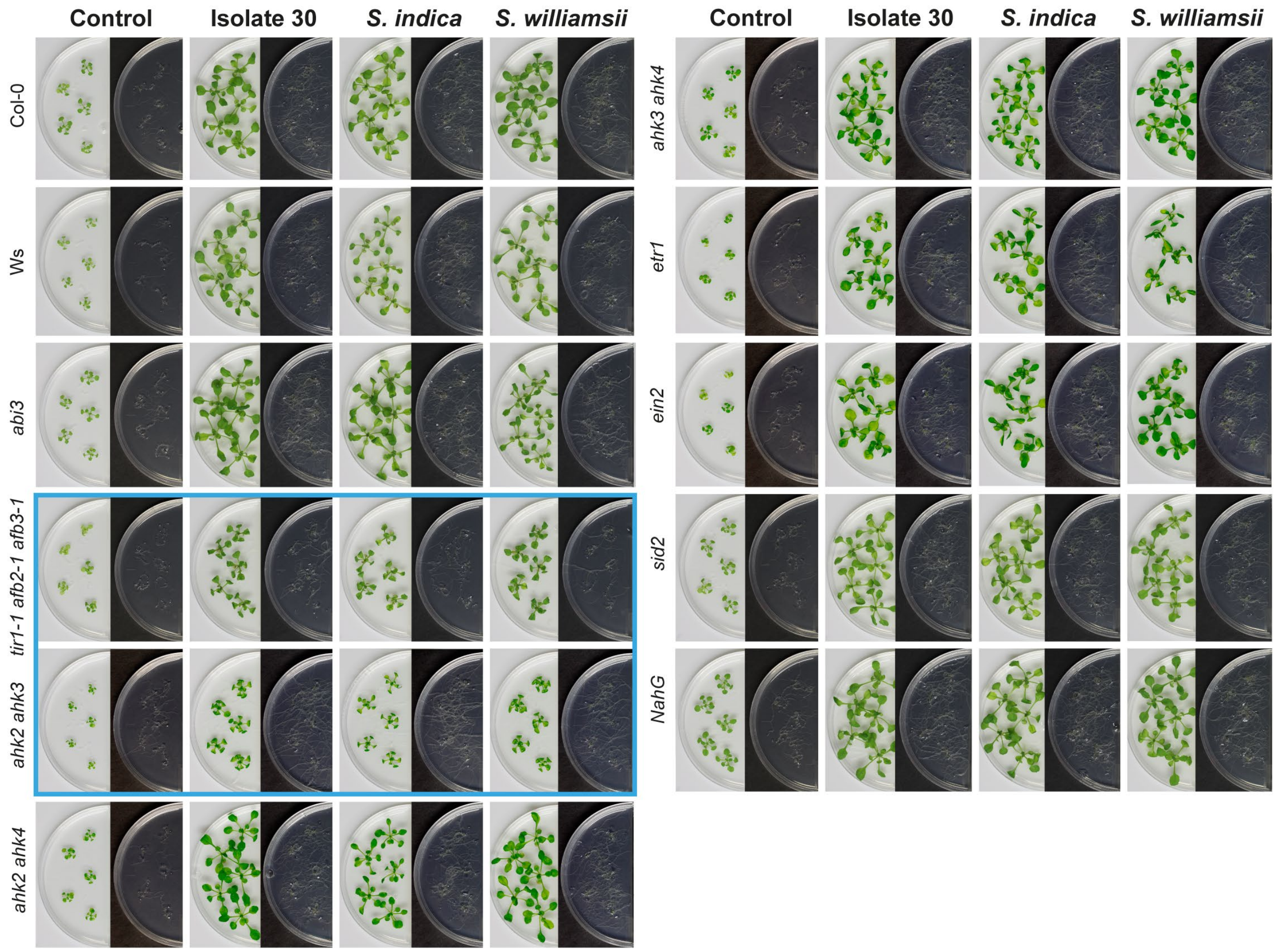

Figure S18. Growth responses of *Arabidopsis* wild-type plants (Col-0 and Ws) and hormone signaling and biosynthesis mutants to volatiles emitted by isolate 30, *S. indica* and *S. williamsii* after ten days of co-cultivation in a split-plate assay. Corresponding shoot fresh weights, expressed relative to the control, are shown in Figure 8A. The two least responsive mutant lines are marked with a blue rectangle. Plates were sealed with Breathe-Easy film.

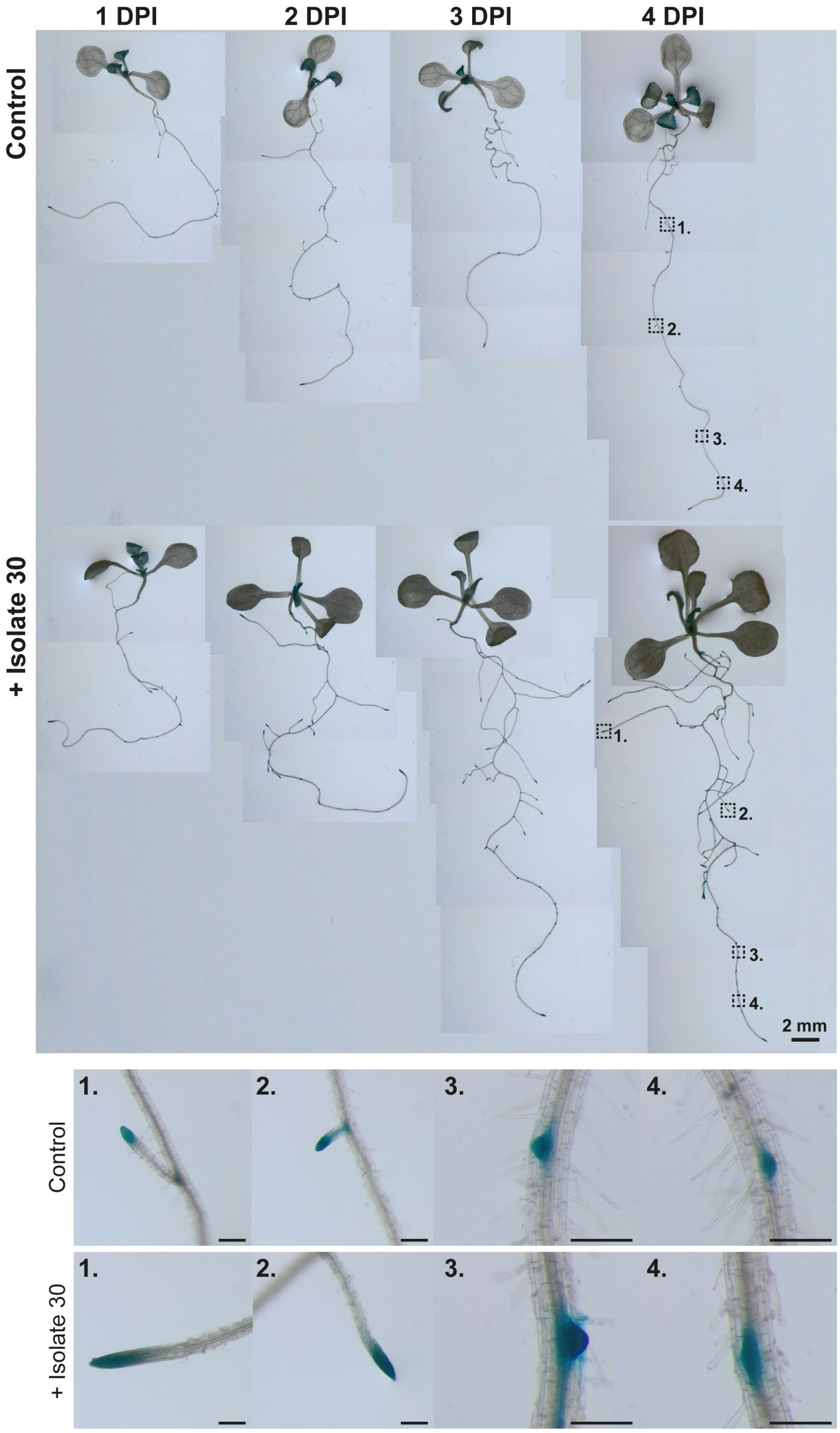

Figure S19. GUS expression patterns in control and VOC-exposed *CYCB1;1::GUS Arabidopsis* seedlings after one to four days of co-cultivation with *Serendipita* isolate 30 in a split-plate assay. The dark color of the leaves in the VOC-exposed plants is caused by massive accumulation of starch. The panels on the bottom show a stronger and more extensive GUS expression in the lateral root tips of the VOC-treated plant (insets 1 and 2), as well as in the newly established lateral roots/primordia arising from the pericycle (insets 3 and 4). DPI, days post 'inoculation' (start of co-cultivation). Scale bars in bottom panels = 200 µm.

4 Supplementary References

Alonso, J. M., Hirayama, T., Roman, G., Nourizadeh, S., and Ecker, J. R. (1999). EIN2, a bifunctional transducer of ethylene and stress responses in *Arabidopsis*. *Science* 284, 2148–2152. doi: 10.1126/science.284.5423.2148

Chang, C., Kwok, S. F., Bleecker, A. B., and Meyerowitz, E. M. (1993). *Arabidopsis* ethylene-response gene *ETR1*: similarity of product to two-component regulators. *Science* 262, 539–544. doi: 10.1126/science.8211181

Colón-Carmona, A., You, R., Haimovitch-Gal, T., and Doerner, P. (1999). Spatio-temporal analysis of mitotic activity with a labile cyclin-GUS fusion protein. *Plant J.* 20, 503–508. doi: 10.1046/j.1365-313x.1999.00620.x

D'Agostino, I. B., Deruère, J., and Kieber, J. J. (2000). Characterization of the *Arabidopsis* response regulator gene family to cytokinin. *Plant Physiol.* 124, 1706–1717. doi: 10.1104/pp.124.4.1706

Dharmasiri, N., Dharmasiri, S., Weijers, D., Lechner, E., Yamada, M., Hobbie, L., et al. (2005). Plant development is regulated by a family of auxin receptor F box proteins. *Dev. Cell* 9, 109–119. doi: 10.1016/j.devcel.2005.05.014

Estévez, J. M., Cantero, A., Romero, C., Kawaide, H., Jiménez, L. F., Kuzuyama, T., et al. (2000). Analysis of the expression of *CLA1*, a gene that encodes the 1-Deoxyxylulose 5-Phosphate Synthase of the 2-C-Methyl-D-Erythritol-4-phosphate pathway in *Arabidopsis*. *Plant Physiol.* 124, 95–104. doi: 10.1104/pp.124.1.95

Friedrich, L., Vernooij, B., Gaffney, T., Morse, A., and Ryals, J. (1995). Characterization of tobacco plants expressing a bacterial salicylate hydroxylase gene. *Plant Mol. Biol.* 29, 959–968. doi: 10.1007/BF00014969

Graus, M., Müller, M., and Hansel, A. (2010). High resolution PTR–TOF quantification and formula confirmation of VOC in real time. *J. Am. Soc. Mass Spectrom.* 21, 1037–1044. doi: 10.1016/j.jasms.2010.02.006

Johnson, J. M., Sherameti, I., Ludwig, A., Nongbri, P. L., Sun, C., Lou, B., et al. (2011). Protocols for *Arabidopsis thaliana* and *Piriformospora indica* co-cultivation - a model system to study plant beneficial traits. *Endocyt. Cell Res.* 21, 101–113.

Jordan, A., Haidacher, S., Hanel, G., Hartungen, E., Märk, L., Seehauser, H., et al. (2009). A high resolution and high sensitivity proton-transfer-reaction time-of-flight mass spectrometer (PTR-TOF-MS). *Int. J. Mass Spectrom.* 286, 122–128. doi: 10.1016/j.ijms.2009.07.005

Koornneef, M., Reuling, G., and Karssen, C. M. (1984). The isolation and characterization of abscisic acid-insensitive mutants of *Arabidopsis thaliana*. *Physiol. Plant.* 61, 377–383. doi: 10.1111/j.1399-3054.1984.tb06343.x

Lindinger, W., Hansel, A., and Jordan, A. (1998). On-line monitoring of volatile organic compounds at pptv levels by means of protontransfer-reaction mass spectrometry (PTR-MS) – Medical applications, food control and environmental research. *Int. J. Mass Spectrom. Ion Processes* 173, 191–241. doi: 10.1016/S0168-1176(97)00281-4

Mandel, M. A., Feldmann, K. A., Herrera-Estrella, L., Rocha-Sosa, M., and León, P. (1996). *CLA1*, a novel gene required for chloroplast development, is highly conserved in evolution. *Plant J.* 9, 649–658. doi: 10.1046/j.1365-313x.1996.9050649.x

Motohashi, R., Nagata, N., Ito, T., Takahashi, S., Hobo, T., Yoshida, S., et al. (2001). An essential role of a TatC homologue of a ΔpH-dependent protein transporter in thylakoid membrane formation during chloroplast development in *Arabidopsis thaliana*. *Proc. Natl. Acad. Sci. U.S.A.* 98, 10 499–10 504. doi: 10.1073/pnas.181304598

Motohashi, R., Yamazaki, T., Myouga, F., Ito, T., Ito, K., Satou, M., et al. (2007). Chloroplast ribosome release factor 1 (AtcpRF1) is essential for chloroplast development. *Plant Mol. Biol.* 64, 481–497. doi: 10.1007/s11103-007-9166-7

Parry, G., Calderon-Villalobos, L. I., Prigge, M., Peret, B., Dharmasiri, S., Itoh, H., et al. (2009). Complex regulation of the TIR1/AFB family of auxin receptors. *Proc. Natl. Acad. Sci. U.S.A.* 106, 22 540–22 545. doi: 10.1073/pnas.0911967106

Pham, G. H., Kumari, R., Singh, A., Malla, R., Prasad, R., Sachdev, M., et al. (2004). "Axenic cultures of *Piriformospora indica*," in Plant Surface Microbiology, eds. A. Varma, L. Abbott, D. Werner, and R. Hampp (Berlin, Heidelberg: Springer-Verlag), 593–616.

Riefler, M., Novak, O., Strnad, M., and Schmülling, T. (2006). *Arabidopsis* cytokinin receptor mutants reveal functions in shoot growth, leaf senescence, seed size, germination, root development, and cytokinin metabolism. *Plant Cell* 18, 40–54. doi: 10.1105/tpc.105.037796

Sherameti, I., Tripathi, S., Varma, A., and Oelmuller, R. (2008). The root-colonizing endophyte *Pirifomospora indica* confers drought tolerance in *Arabidopsis* by stimulating the expression of drought stress-related genes in leaves. *Mol. Plant-Microbe Interact.* 21, 799–807. doi: 10.1094/MPMI-21-6-0799

Ulmasov, T., Murfett, J., Hagen, G., and Guilfoyle, T. J. (1997). Aux/IAA proteins repress expression of reporter genes containing natural and highly active synthetic auxin response elements. *Plant Cell* 9, 1963–1971. doi: 10.1105/tpc.9.11.1963

Venneman, J., Audenaert, K., Verwaeren, J., Baert, G., Boeckx, P., Moango, A. M., et al. (2017). Rhizospheric Soils as a Rich Source of New Plant Growth-Promoting endophytic *Piriformospora* Isolates. *Front. Microbiol.* 8:212. doi: 10.3389/fmicb.2017.00212

Walgraeve, C., Demeestere, K., Dewulf, J., Van Huffel, K., and Van Langenhove, H. (2011). Uptake rate behavior of tube-type passive air samplers for volatile organic compounds under controlled atmospheric conditions. *Atmos. Environ.* 45, 5872–5879. doi: 10.1016/j.atmosenv.2011.06.069

Wildermuth, M. C., Dewdney, J., Wu, G., and Ausubel, F. M. (2001). Isochorismate synthase is required to synthesize salicylic acid for plant defence. *Nature* 414, 562–565. doi: 10.1038/35107108
